# Your height affects your health: genetic determinants and health-related outcomes in Taiwan

**DOI:** 10.1186/s12916-022-02450-w

**Published:** 2022-07-13

**Authors:** Jian-Shiun Chiou, Chi-Fung Cheng, Wen-Miin Liang, Chen-Hsing Chou, Chung-Hsing Wang, Wei-De Lin, Mu-Lin Chiu, Wei-Chung Cheng, Cheng-Wen Lin, Ting-Hsu Lin, Chiu-Chu Liao, Shao-Mei Huang, Chang-Hai Tsai, Ying-Ju Lin, Fuu-Jen Tsai

**Affiliations:** 1 PhD Program for Health Science and Industry, College of Health Care, China Medical University, Taichung, Taiwan; 2 Department of Health Services Administration, China Medical University, Taichung, Taiwan; 3 Big Data Center and Genetic Center, Department of Medical Research, China Medical University Hospital, Taichung, Taiwan; 4 Department of Pediatrics, China Medical University Children’s Hospital, Taichung, Taiwan; 5 School of Medicine, China Medical University, Taichung, Taiwan; 6 School of Post Baccalaureate Chinese Medicine, China Medical University, Taichung, Taiwan; 7 School of Chinese Medicine, China Medical University, Taichung, Taiwan; 8 PhD Program for Cancer Biology and Drug Discovery, China Medical University and Academia Sinica, Taichung, Taiwan; 9 Research Center for Cancer Biology, China Medical University, Taichung, Taiwan; 10 Department of Medical Laboratory Science and Biotechnology, China Medical University, Taichung, Taiwan; 11 Department of Biotechnology and Bioinformatics, Asia University, Taichung, Taiwan

**Keywords:** Height, Genome-wide association studies, Genetic single nucleotide polymorphisms, Polygenic risk score, Health-related outcomes

## Abstract

**Background:**

Height is an important anthropometric measurement and is associated with many health-related outcomes. Genome-wide association studies (GWASs) have identified hundreds of genetic loci associated with height, mainly in individuals of European ancestry.

**Methods:**

We performed genome-wide association analyses and replicated previously reported GWAS-determined single nucleotide polymorphisms (SNPs) in the Taiwanese Han population (Taiwan Biobank; *n* = 67,452). A genetic instrument composed of 251 SNPs was selected from our GWAS, based on height and replication results as the best-fit polygenic risk score (PRS), in accordance with the clumping and *p*-value threshold method. We also examined the association between genetically determined height (PRS_251_) and measured height (phenotype). We performed observational (phenotype) and genetic PRS_251_ association analyses of height and health-related outcomes.

**Results:**

GWAS identified 6843 SNPs in 89 genomic regions with genome-wide significance, including 18 novel loci. These were the most strongly associated genetic loci (*EFEMP1*, *DIS3L2*, *ZBTB38*, *LCORL*, *HMGA1*, *CS*, and *GDF5*) previously reported to play a role in height. There was a positive association between PRS_251_ and measured height (*p* < 0.001). Of the 14 traits and 49 diseases analyzed, we observed significant associations of measured and genetically determined height with only eight traits (*p* < 0.05/[14 + 49]). Height was positively associated with body weight, waist circumference, and hip circumference but negatively associated with body mass index, waist-hip ratio, body fat, total cholesterol, and low-density lipoprotein cholesterol (*p* < 0.05/[14 + 49]).

**Conclusions:**

This study contributes to the understanding of the genetic features of height and health-related outcomes in individuals of Han Chinese ancestry in Taiwan.

**Supplementary Information:**

The online version contains supplementary material available at 10.1186/s12916-022-02450-w.

## Background

Height is the growth phenotype during the entire developmental period from infancy to adulthood and becomes relatively stable in adulthood [1–3]. Previous studies have reported that social and environmental factors can influence height. Some of these factors include educational attainment, smoking, alcohol consumption, and regular exercise [3–9]. Higher levels of education [4, 5] and regular exercise [9] are associated with increased growth and height. In contrast, exposure to smoking or drinking may cause bone mass loss, reduced growth, and reduced height [10–12]. Height is determined by polygenic inheritance under complex and multi-locus genetic regulation [13–15]. Genome-wide association studies (GWAS) of height have identified hundreds of genetic loci (or single nucleotide polymorphisms [SNPs]) with genome-wide significance, especially in individuals of European ancestry [15–26]. These identified genetic loci are associated with proteins of the tyrosine phosphatase family, insulin-like growth factors, proteins involved in skeletal development and mitosis, fibroblast growth factors, the Wnt/β-catenin pathway, Hedgehog signaling, and cancer-associated pathways. These findings highlight the polygenic, complex, and multilocus genetic regulation of height.

Height is associated with several health-related outcomes later in life [27–34]. For instance, taller people tend to have a higher risk of cancer [28] and cancer-related mortality [27] but have a reduced risk of CVD [27, 29], CVD-related mortality [27, 29], type 2 diabetes [34], better retention of cognitive function [30–32], and healthy aging [33]. Height can be measured as a genetic component using a polygenic risk score (PRS). PRS is the sum of the weighted risk alleles from a combination of independent SNPs, usually with genome-wide significance, derived from GWAS results [13, 35, 36]. PRS serves as a genetic instrument variable and can be used to assess associations with health-related outcomes without confounding [37, 38]. Genetically determined taller height (in those with European ancestry) is also associated with an increased risk of cancers [39–44] and cancer-related mortality [45, 46] but a reduced risk of CVD [42, 47–50]. The precise shared genetic loci between height and health-related outcomes are yet to be elucidated, especially in individuals of Han Chinese ancestry. In addition, the mechanisms on how shared genetic loci contribute to both height and health-related outcomes remain unclear.

Therefore, this study aimed to identify the genetic architecture for height in individuals from the Taiwan Biobank—a community-based biobank in Taiwan. We also performed observational and genetic PRS analyses of height and health-related outcomes.

## Methods

### Taiwan Biobank

The Taiwan Biobank is a database for phenotypic and genomic measurements of the Taiwanese population that was established in 2012. The study recruited volunteers aged 30–70 years with no history of malignancy at enrollment (Twbiobank; https://www.twbiobank.org.tw/new_web/) [51, 52]. All volunteers were residents of Taiwan and provided informed consent. Participants completed questionnaires and underwent interviews, anthropometric measurements, and blood and urine tests to collect demographic, lifestyle, and genomic data.

### Taiwan Biobank phenotypes

Anthropometric measurements, including height, body weight, waist circumference, hip circumference, and body fat percentage, were obtained from participants in the Taiwan Biobank (Additional file 1: Table S1). Body mass index (BMI) was calculated as BMI = body weight/body height^2^. The waist-hip ratio (WHR) was calculated as WHR = waist circumference/ hip circumference. Anthropometric measurements were stratified by sex and analyzed using the mean and standard deviation (SD), where data were normalized to one SD before further analysis.

Blood pressure and lipid and glucose levels were quantitatively measured in participants in the Taiwan Biobank. Systolic blood pressure (SBP), diastolic blood pressure (DBP), total cholesterol (TC), triglyceride (TG), low-density lipoprotein cholesterol (LDL-C), high-density lipoprotein cholesterol (HDL-C), fasting glucose, and hemoglobin (Hb) A1c levels were obtained (Additional file 1: Table S1).

Participants were asked to report their health status using questionnaires and interviews. According to participants’ self-reported health status (comorbidities) in the Taiwan Biobank, 10 broad categories of 49 diseases were investigated in our study as follows (Additional file 1: Table S1): (1) orthopedic or joint disorders: osteoporosis, arthritis, rheumatoid arthritis, osteoarthritis, and gout; (2) lung and respiratory diseases: asthma and emphysema or chronic bronchitis; (3) cardiovascular diseases: valvular heart disease, coronary artery disease, heart arrhythmia, cardiomyopathy, congenital heart defect, other type of heart disease, hyperlipidemia, hypertension, and stroke; (4) diabetes: type 1 diabetes and type 2 diabetes; (5) digestive diseases: peptic ulcer disease, gastroesophageal reflux disease, and irritable bowel syndrome; (6) Mental or emotional disorders: depression, bipolar disorder, postpartum depression, obsessive-compulsive disorder, alcohol addiction or drug abuse, and schizophrenia; (7) nervous system disorders: epilepsy, migraine, multiple sclerosis, Parkinson’s disorder, and dementia; (8) other types of disease: gallstones, kidney stones, kidney failure, and vertigo; (9) eye diseases: cataract, glaucoma, dry eye syndrome, retinal detachment, floaters, blindness, color blindness, and others; and (10) female diseases: severe menstrual cramps, uterine fibroids, ovarian cysts, endometriosis, and uterine/cervical polyps.

### Study population

**Fig. 1:**
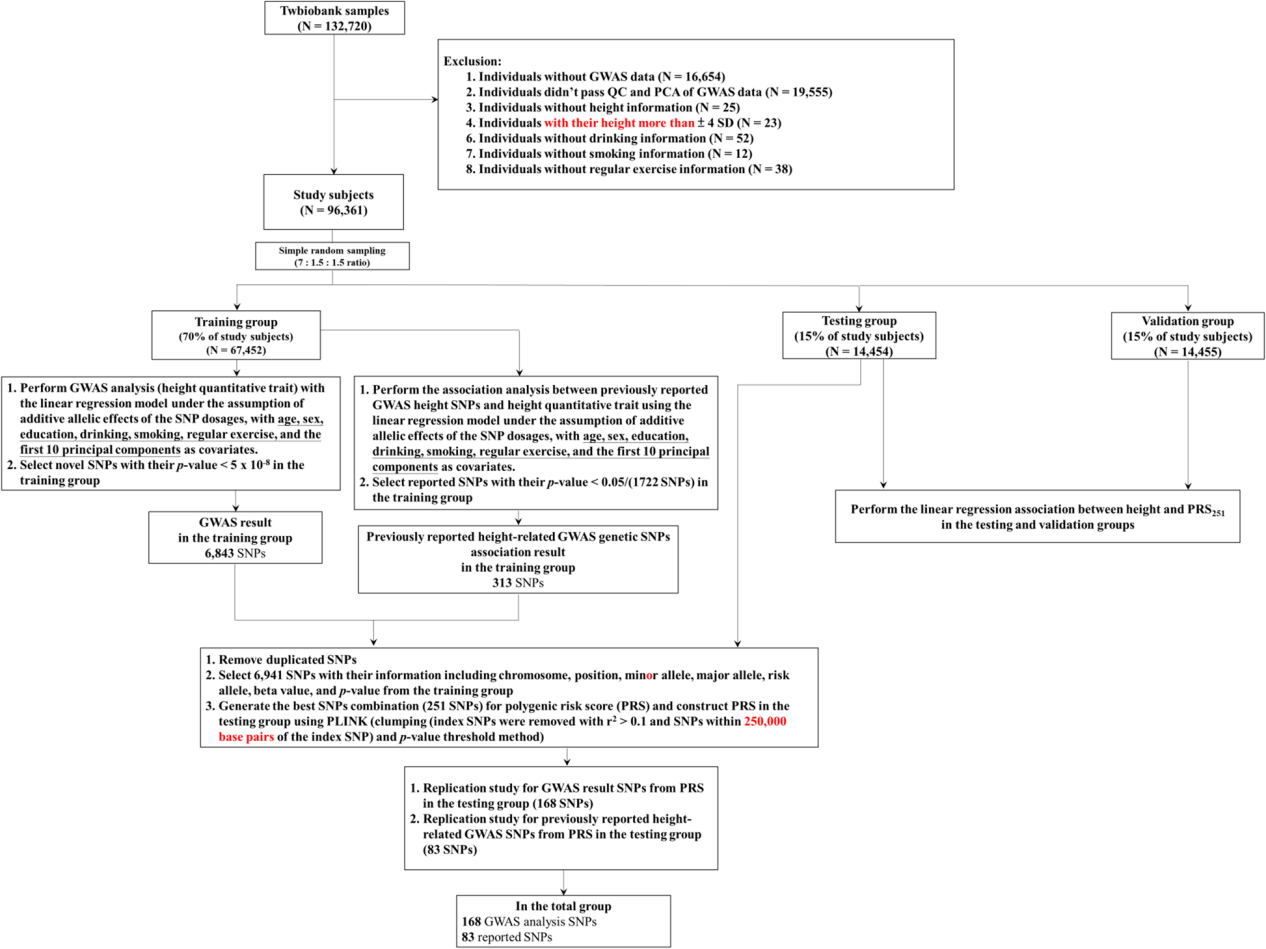
Flowchart of the study design and analysis process

A total of 132,720 participants were selected from the Taiwan Biobank (Fig. 1). The exclusion criteria were as follows: (1) individuals who did not have GWAS data (*N* = 16,654), (2) individuals who did not pass the quality control (QC) and principal component analysis (PCA) of GWAS data (*N* = 19,555), (3) individuals who did not have height information (*N* = 25), (4) individuals with their height more than ±4 SD (*N* = 23), (5) individuals without drinking information (*N* = 52), (6) individuals without smoking information (*N* = 12), and (7) individuals without regular exercise information (*N* = 38). The criteria for drinking included current drinkers for at least 6 months; smoking criteria included current smokers for at least 6 months; finally, regular exercise criteria included participants performing regular exercise currently, for at least 6 months.

**Fig. 2:**
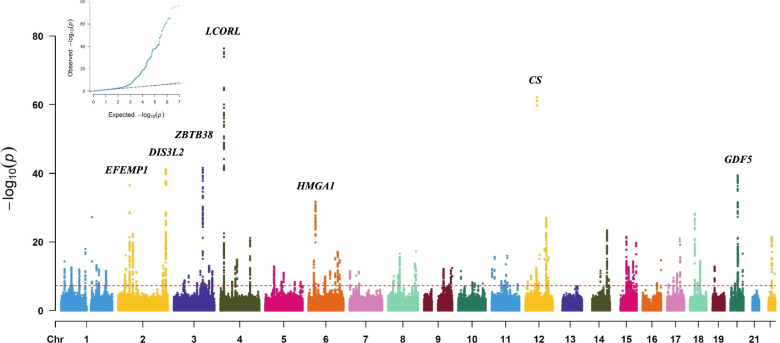
Manhattan plot for height with Han Chinese ancestry

**Fig. 3:**
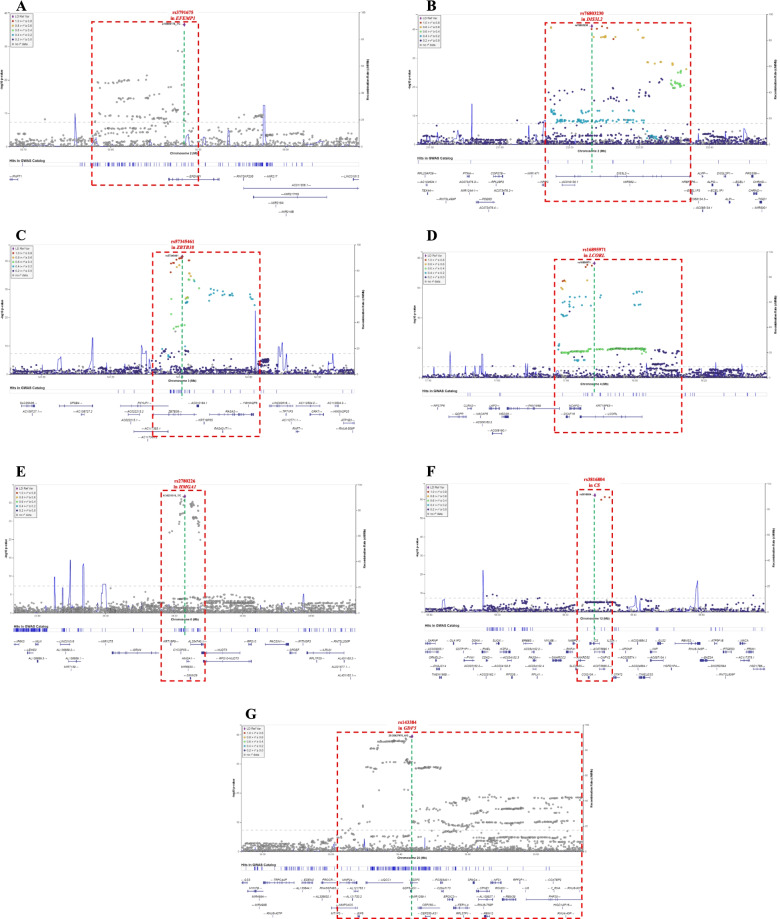
Regional plots for the independent signals at seven genetic loci for height in individuals with Han Chinese ancestry. **A** rs3791675 in *EGF containing fibulin extracellular matrix protein 1* (*EFEMP1*). **B** rs76803230 in *DIS3 like 3′-5′ exoribonuclease 2* (*DIS3L2*). **C** rs57345461 in *zinc finger and BTB domain containing 38* (*ZBTB38*). **D** rs16895971 in *ligand dependent nuclear receptor corepressor like* (*LCORL*). **E** rs2780226 in *high mobility group AT-hook 1* (*HMGA1*). **F** rs3816804 in *citrate synthase* (*CS*). **G** rs143384 in *growth differentiation factor 5* (*GDF5*). Each plot shows the –log10 *p*-value on the *y*-axis for each SNP and the SNP position in the genome region on the *x*-axis. The top significant SNP is shown by a purple diamond; genes in its proximity are shown below each plot. LD with nearby SNPs is measured using *R*^2^ values, according to the 1000 Genomes Project Phase 3 East Asia Summit data, and is indicated by the color of each circle

Finally, 96,361 participants of Han Chinese ancestry were included in this study (Fig. 1) and assigned to the training, testing, and validation groups using a simple random sampling method (7:1.5:1.5 ratio). The training group (*N* = 67,452 participants) comprised 70% of the total study population and underwent GWAS based on height (Additional file 2: Table S2; Figs. 2 and 3). Before the height GWAS analysis, the measured height (phenotype) was stratified by sex and subsequently mean-centered and normalized to one SD. GWAS for height was then performed using a linear regression model with the assumption of additive allelic effects of SNP dosages, with adjusted covariates including age, sex, education, drinking, smoking, regular exercise, and the first 10 PCAs (Additional file 4: Fig. S2), using the PLINK software (version 1.9, 2.0) [3–9, 20, 53–56]. A genome-wide significance value was used (*p* < 5.00E−8 for the additive test).

We also ensured that our GWAS findings of the training group replicated previously identified height-associated genetic variants (https://www.ebi.ac.uk/gwas/efotraits/EFO_0004339). The reported GWAS height-associated genetic variants were mainly from individuals of European ancestry (excluding the genetic variants of infant or child height traits) and were downloaded from the GWAS catalog website. After removal of the repeated SNPs, 1722 reported GWAS body height-related SNPs were obtained from the GWAS catalog (Additional file 3: Table S3). These SNPs were replicated in our cohort, and we further identified 313 GWAS SNPs associated with height in our cohort (*p* < 0.05/1722 SNPs) (Additional file 3: Table S3).

**Fig. 4:**
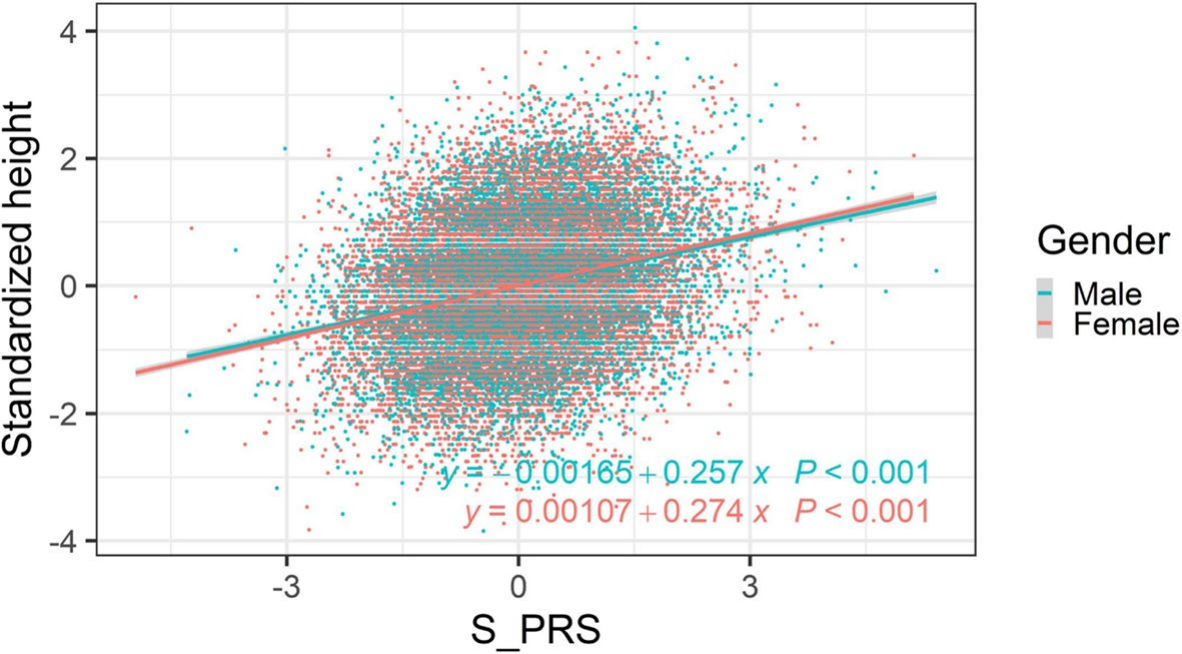
Association between genetically determined height (PRS_251_) and measured height (phenotype). Measured height (cm) and calculated polygenic risk score (PRS) for height in the testing and validation groups were stratified by sex, mean-centered, and normalized to one standard deviation (SD), respectively (males, *N* = 10,919; females, *N* = 17,990). The normalized measured height is shown on the *y*-axis and normalized genetically determined height (PRS_251_) is shown on the *x*-axis

The testing group (*N* = 14,454 participants) comprised 15% of the total study population and was used to select the best-fit PRS, to investigate the association between genetically determined height (PRS_251_) and measured height (phenotype) using linear regression analysis (Fig. 4). The validation group (*n* = 14,455 participants) comprised 15% of the total population and was used to determine the association between genetically determined height (PRS_251_) and measured height (phenotype) using linear regression analysis (Fig. 4). This study was approved by the Human Studies Committee of the China Medical University Hospital, Taichung, Taiwan (approval number: CMUH107-REC3-074).

### QC of the original data

Genomic DNA from the Taiwan Biobank was genotyped using Axiom genome-wide TWB1 (653,291 SNPs) or TWB2 (752,921 SNPs) array plates based on the Axiom genome-wide array plate system, according to the manufacturer’s instructions (Affymetrix Inc., Santa Clara, CA, USA). Genotyping was performed at the National Genotyping Center of Academia Sinica, Taipei, Taiwan (http://ncgm.sinica.edu.tw/affymetrix_tech_01.html) (https://www.biobank.org.tw/fd.php).

Genotypic data were then subjected to QC procedures (individual QC and SNP QC) in the Taiwan Biobank (https://www.biobank.org.tw/fd.php). The exclusion criteria for individual QC were as follows: (1) individuals with a missing call rate of > 5%, (2) a heterozygosity rate of >±5 SD, (3) individual identity by descent (IBD) score of ≥ 0.125, and (4) individuals who did not fit the East Asia Summit ancestry PCA. Similar to the results of a previous study [51], most individuals from the Taiwan Biobank were of Han Chinese ancestry. The exclusion criteria for SNP QC were as follows: (1) SNPs with a missing call rate of >5%, (2) SNPs with Hardy–Weinberg equilibrium (HWE) *p*-value of <1 × 10^−5^, and (3) SNPs with a minor allele frequency (MAF) of <5%.

### Imputation

Qualified genotype data were then subjected to an imputation procedure to maximize the number of SNPs in the Taiwan Biobank (https://www.biobank.org.tw/fd.php). First, the SNPs of the qualified genotype data were excluded based on the following criteria: (1) SNPs with MAF of <1%, (2) SNPs with a HWE *p*-value of <1 × 10^−5^, and (3) SNP with a missing call rate > 5% using the PLINK software (versions 1.9 and 2.0, http://zzz.bwh.harvard.edu/plink/). SHAPEIT2 (v2.r790) was used to phase the genotypes into full haplotypes (https://mathgen.stats.ox.ac.uk/genetics_software/shapeit/shapeit.html). Third, imputation was performed using IMPUTE2 (v2.3.1, https://mathgen.stats.ox.ac.uk/impute/impute_v2.html), according to the pooled reference panel [Taiwan Biobank (TWB) + East Asian (EAS)]. The pooled reference panel comprised 973 phased individuals with the TWB panel from the Taiwan Biobank [57] and 504 phased individuals from the EAS panel [57, 58] (The Phase 3 1000 Genomes Project reference panel; The 1000 Genomes Project Consortium, 2010). The pooled reference panel with TWB and EAS ancestry groups was used to improve imputation accuracy [57, 58]. The following imputed SNPs were excluded: (1) SNP with a missing call rate of > 5%, (2) SNPs with MAF < 0.01%, and (3) SNPs with IMPUTE2 information score of < 0.3.

### QC for this study

Imputed GWAS data were obtained from the Taiwan Biobank. In our study, SNP QC and individual QC procedures were applied before GWAS of height (Fig. 1). SNPs were excluded from the SNP QC based on the following criteria: (1) SNP with a missing call rate of > 5%, (2) SNPs with HWE *p*-value of < 1 × 10^−6^; and (3) SNPs with MAF < 0.01%. After SNP QC, the remaining SNPs were used to perform ancestry PCA for the population structure analysis. The exclusion criteria for individual QC were as follows: (1) individual with a missing call rate > 5%, (2) heterozygosity rate > ±5 SD, (3) individual IBD score ≥0.125, and (4) individuals who did not fit the East Asia Summit ancestry PCA. Participants of non-Chinese ancestry, with evidence of relatedness, or with DNA contamination were excluded.

### PRS calculation

The PRS was calculated in the testing group using PLINK software (versions 1.9 and 2.0) [35, 53, 59], based on the statistical results of the 6,941 SNPs in the training group (Fig. 1). The 6941 SNPs comprised SNPs with genome-wide significance (*p* < 5 × 10^−8^) and SNPs that were replicated from previously reported body-height GWAS SNPs (*p* < 0.05/1722 SNPs; Fig. 1).

The 6941 SNPs were then subjected to the clumping procedure (within the range of 250,000 base pairs of the index SNP, where SNPs were removed when *r*^2^ > 0.1), according to the estimated linkage disequilibrium (LD) among the SNPs in the testing group (Additional file 4: Fig. S1A). After clumping, 251 SNPs were obtained. These 251 SNPs were used to select the “best-fit” PRS according to a series of cutoff values for height-associated *p*-value thresholds (including 5 × 10^−15^, 5 × 10^−14^, 5 × 10^−13^, 5 × 10^−12^, 5 × 10^−11^, 5 × 10^−10^, 5 × 10^−9^, 5 × 10^−8^, 5 × 10^−7^, 5 × 10^−6^, and 5 × 10^−5^) in the testing group. The *p*-value cutoff (5 × 10^−5^) was adopted by the “best-fit” PRS with the largest explicable phenotype *r*^2^ using only the PRS (PRS *r*^2^ = 0.0712, SNP number = 251; Additional file 4: Fig. S1A). In total, 251 SNPs were obtained for the best-fit PRS calculations for all participants. For each participant, the genetically determined height (PRS value) was calculated [35, 53, 59] using 251 SNPs obtained after the clumping protocol. Data centering and standardization were also performed for the PRS height data.

### Statistical analyses

Genotype and imputed genotype data were used for GWAS analysis, as previously described. The HWE for the SNPs in the controls was evaluated using chi-square (*χ*^2^) tests. Lewontin’s D and *R*^2^ values were used to evaluate the inter-marker coefficient of LD for haplotype block analysis [60]. The confidence interval (CI) for LD was used to construct haplotype blocks by resampling [61, 62]. LocusZoom was used to plot the resulting significant locus [63].

Measured height (phenotype) served as the exposure variables. Sixty-three health-related outcomes, including 14 traits and 49 diseases, were used as outcome variables. A multivariate linear regression model was made for continuous outcome variables (14 traits), with adjustments for age, sex, education, drinking, smoking, regular exercise, and 10 PCAs [3–9, 20]. Multivariate logistic regression analysis was performed for binary outcome variables (49 diseases), with adjustments for age, sex, education, drinking, smoking, regular exercise, and 10 PCAs [3–9, 20].

The genetically determined height (PRS_251_) also served as the exposure variable. Sixty-three health-related outcomes, including 14 traits and 49 diseases, were used as outcome variables. A multivariate linear regression model was performed for continuous outcome variables (14 traits), with adjustments for age, sex, education, drinking, smoking, regular exercise, and 10 PCAs [3–9, 20]. Multivariate logistic regression analysis was performed for binary outcome variables (49 diseases), with adjustments for age, sex, education, drinking, smoking, regular exercise, and 10 PCAs [3–9, 20]. PLINK software (versions 1.9 and 2.0) and R packages for Windows were used for all statistical analyses.

## Results

### GWAS of the quantitative trait of height in Han Chinese in Taiwan

The Manhattan and QQ plots for the adult-height GWAS results are shown in Fig. 2. GWAS association analysis identified 6843 SNPs in 89 genomic regions with genome-wide significance (*p* < 5.00E−08 [5 × 10^−8^], not shown). The top lead SNPs were selected in 89 genomic regions with significant associations (*p* < 5 × 10^−8^) using an LD of < 0.2 (Additional file 2: Table S2). Among these, 18 novel lead SNPs within 18 novel regions, 48 novel lead SNPs within 48 reported regions, and 23 lead SNPs within 23 reported regions were found (Additional file 2: Table S2). Moreover, among these 89 genomic regions, the seven lead SNPs were located within seven genetic loci (Fig. 2). These seven genetic loci were located near the following genes: *EGF-containing fibulin extracellular matrix protein 1* (*EFEMP1*), *DIS3 like 3′-5′ exoribonuclease 2* (*DIS3L2*), *zinc finger and BTB domain containing 38* (*ZBTB38*), *ligand-dependent nuclear receptor corepressor like* (*LCORL*), *high-mobility group AT-hook 1* (*HMGA1*), *citrate synthase* (*CS*), and *growth differentiation factor 5* (*GDF5*). The seven lead SNPs from these seven genetic loci are shown in Additional file 2: Table S2.

Regional plots of the lead SNPs and their neighboring SNPs from these seven genetic loci are shown in Fig. 3. Among them, two lead SNPs were novel (Fig. 3B, C; chromosome 2, rs76803230 in *DIS3L2*; chromosome 3, rs57345461 in *ZBTB38*), whereas the remaining five lead SNPs were previously reported (Fig. 3A, D–G). On chromosome 2, the lead SNP rs76803230 was located in the intronic region of *DIS3L2* (risk allele: T, beta = 0.0681, [95% CI:0.0583–0.0780], *p* = 7.47E−42 [7.47 × 10^−42^]; Additional file 2: Table S2; Fig. 3B). On chromosome 3, the lead SNP rs57345461 was located in the intronic region of *ZBTB38* (risk allele: T, beta = 0.0723, [95% CI: 0.0619–0.0827], *p* = 2.54E−42 [2.54 × 10^−42^]; Additional file 2: Table S2; Fig. 3C).

In the previously reported SNPs on chromosome 2, only a handful reached genome-wide significance associated with height, where the lead SNP rs3791675 was located in the intronic region of *EFEMP1* (risk allele: C; training group: beta = 0.0753, [95% CI: 0.0638–0.0869], *p* = 2.78E-37 [2.78 × 10^−37^]; Additional file 2: Table S2; Fig. 3A). This SNP has been associated with body height, BMI-adjusted waist circumference, pelvic organ prolapse, and BMI-adjusted WHR [23, 64–66]. On chromosome 4, the lead SNP rs16895971 was located in the 3-untranslated region (3UTR) of *LCORL* (risk allele: T, beta = 0.1018, [95% CI: 0.0911–0.1125], *p* = 3.69E−77 [3.69 × 10^−77^]; Additional file 2: Table S2; Fig. 3D). This SNP has been associated with body height in East Asians [67]. On chromosome 6, the lead SNP rs2780226 was located in the 5 untranslated region (UTR) of *HMGA1* (risk allele: C, beta = 0.0948, [95% CI: 0.0791–0.1104], *p* = 1.75E−32 [1.75 × 10^−32^]; Additional file 2: Table S2; Fig. 3E). This SNP has been associated with body height, BMI-adjusted waist circumference, and birth weight [15, 68, 69]. On chromosome 12, the lead SNP rs3816804 was located in the intronic region of *CS* (risk allele: C, beta = 0.1124, [95% CI: 0.0993–0.1255], *p* = 6.35E−63 [6.35 × 10^−63^]; Additional file 2: Table S2; Fig. 3F). This SNP has also been associated with body height in East Asians [70]. On chromosome 20, the lead SNP rs143384 was located in the 5-UTR of *GDF5* (risk allele: G, beta = 0.0738, [95% CI: 0.0629–0.0847], *p* = 3.61E−40 [3.61 × 10^−40^]; Additional file 2: Table S2; Fig. 3G). This SNP has been associated with body height, BMI-adjusted hip circumference, BMI-adjusted WHR, and body fat [15, 64, 71, 72].

### Replication of previously reported GWAS-determined SNPs in the Taiwan Han population

The previously reported GWAS-determined SNPs for height were obtained from the GWAS catalog (https://www.ebi.ac.uk/gwas/efotraits/EFO_0004339) and used to replicate the reported SNPs in the training group using the linear regression model, as described previously. In this study, an association analysis identified 313 SNPs that were significantly associated with height (*p* < 0.05/1722 SNPs; Additional file 3: Table S3).

In this study, GWAS-identified 6843 SNPs, and 313 of the reported SNPs were combined. After removing duplicate SNPs, 6941 SNPs were associated with height (Fig. 1). These 6941 SNPs were then applied to exclude SNPs with strong LD and to select the best SNP combination for the best-fit PRS calculation in the testing group, using PLINK software (versions 1.9 and 2.0) [53]. This resulted in the identification of independent genetic signals for the best-fit PRS with 251 SNPs. These 251 SNPs included 168 GWAS-identified SNPs (Table 1) and 83 previously reported GWAS-determined SNPs (Table 2). These results show that 168 novel GWAS-identified SNPs and 83 reported SNPs were associated with height in individuals of Han Chinese ancestry in Taiwan.

**Table 1:** Newly identified SNPs associated with height in Taiwan

No.	rs ID	Nearest Gene	Chr.	Position	Minor allele	Major allele	Risk allele	Training group (***N*** = 67,452) (***p*** < 5 ×10^**−8**^)				Testing group (***N*** = 14,454)			
								Beta	95% CI		***P***-value	Beta	95% CI		***P***-value
1	rs56265117	*MFAP2*	1	16980428	C	T	C	0.040	0.030	0.050	4.66E−15	0.042	0.021	0.064	1.31E−04
2	rs76910682		1	50408548	A	G	A	0.037	0.025	0.050	7.45E−09	0.010	−0.018	0.037	4.97E−01
3	rs12098132	*FAF1*	1	50661852	A	C	A	0.060	0.044	0.077	8.05E−13	0.049	0.013	0.086	8.04E−03
4	rs61115731	*FAF1*	1	50932429	C	G	C	0.061	0.044	0.077	2.48E−13	0.054	0.018	0.090	2.96E−03
5	rs140830175	*LINC01562*	1	51197120	T	C	T	0.058	0.041	0.075	3.85E−11	0.054	0.016	0.092	5.26E−03
6	rs4926705		1	55953858	A	G	A	0.039	0.025	0.053	4.55E−08	0.045	0.015	0.075	3.12E−03
7	rs3806340	*PKN2-AS1*	1	88683110	G	T	T	0.036	0.026	0.045	9.75E−13	0.015	−0.006	0.037	1.59E−01
8	rs7530513	*KYAT3*	1	88944228	A	G	G	0.033	0.023	0.044	7.58E−11	0.027	0.005	0.049	1.64E−02
9	rs7513580		1	118307286	A	G	G	0.046	0.036	0.057	1.01E−18	0.041	0.019	0.064	2.98E−04
10	rs10489289	*DNM3*	1	172254949	C	T	C	0.043	0.032	0.054	6.19E−14	0.028	0.003	0.052	2.68E−02
11	rs12047271		1	184044357	C	T	C	0.036	0.026	0.046	8.73E−13	0.052	0.030	0.073	2.52E−06
12	rs1046017	*TGFB2, TGFB2-OT1*	1	218443793	C	G	G	0.039	0.028	0.050	2.75E−12	0.023	−0.001	0.047	5.71E−02
13	rs7538503		1	219615188	G	A	G	0.037	0.025	0.049	1.13E−09	0.029	0.003	0.054	2.92E−02
14	rs2367623	*LTBP1*	2	33202983	A	C	A	0.035	0.024	0.045	2.80E−11	0.021	−0.001	0.043	6.47E−02
15	rs143098957	*LTBP1*	2	33263857	T	G	G	0.043	0.030	0.056	1.02E−10	0.039	0.011	0.068	6.66E−03
16	rs17019115	*FEZ2*	2	36575874	C	G	G	0.029	0.019	0.039	2.49E−08	0.009	−0.013	0.031	4.11E−01
17	rs4670703		2	37385957	C	A	C	0.036	0.026	0.046	6.39E−13	0.012	−0.010	0.033	2.84E−01
18	rs79121675		2	55724807	A	C	A	0.085	0.056	0.114	6.55E−09	0.077	0.016	0.139	1.41E−02
19	rs146446706	*LOC112268416, EFEMP1*	2	55870880	T	C	T	0.151	0.111	0.191	2.34E−13	0.030	−0.059	0.119	5.04E−01
20	rs1824305		2	71179325	T	C	C	0.051	0.041	0.061	4.96E−23	0.047	0.025	0.068	2.55E−05
21	rs57092473		2	71440419	A	G	G	0.044	0.034	0.054	4.47E−18	0.045	0.024	0.067	4.20E−05
22	rs1913671	*EIF2AK3*	2	88600365	T	C	C	0.035	0.025	0.044	5.04E−12	0.027	0.006	0.048	1.22E−02
23	rs1118150	*DIRC3*	2	217415545	A	C	A	0.034	0.022	0.045	5.54E−09	0.013	−0.011	0.038	2.90E−01
24	rs484085	*USP37*	2	218531961	C	T	T	0.036	0.024	0.047	1.19E−09	0.020	−0.005	0.045	1.10E−01
25	rs422702	*CFAP65*	2	219037931	C	T	C	0.036	0.026	0.046	5.30E−13	0.045	0.023	0.066	4.45E−05
26	rs374935766		2	231850100	C	G	G	0.175	0.117	0.234	4.07E−09	0.102	−0.019	0.222	9.82E−02
27	rs33994242		2	231915948	G	A	A	0.043	0.028	0.058	2.01E−08	0.059	0.027	0.091	3.61E−04
28	rs76803230	*DIS3L2*	2	232063990	G	T	T	0.068	0.058	0.078	7.47E−42	0.078	0.057	0.099	7.90E−13
29	rs146229392	*DIS3L2*	2	232064573	C	G	G	0.092	0.061	0.123	6.79E−09	0.079	0.013	0.144	1.84E−02
30	rs3748967	*DIS3L2*	2	232333663	A	G	G	0.055	0.045	0.065	6.35E−27	0.061	0.040	0.083	2.85E−08
31	rs894857163	*GIGYF2*	2	232726985	T	C	C	0.280	0.191	0.369	6.79E−10	0.248	0.056	0.440	1.15E−02
32	rs11130111	*CCDC12*	3	46968315	C	T	C	0.028	0.018	0.038	2.58E−08	0.019	−0.002	0.040	7.92E−02
33	rs12495173	*KIF9-AS1, KIF9*	3	47241409	T	C	T	0.040	0.026	0.054	1.12E−08	0.042	0.012	0.072	6.08E−03
34	rs1209842003		3	52186981	T	G	G	0.263	0.177	0.350	2.53E−09	0.387	0.194	0.580	8.58E−05
35	rs754871503	*NT5DC2*	3	52525012	C	T	T	0.268	0.181	0.355	1.38E−09	0.464	0.269	0.660	3.22E−06
36	rs1328122506	*SFMBT1*	3	52913981	A	C	C	0.260	0.173	0.346	3.59E−09	0.471	0.280	0.662	1.34E−06
37	rs13086339	*RYBP*	3	72428668	C	A	C	0.033	0.023	0.043	6.55E−11	0.013	−0.008	0.035	2.22E−01
38	rs11710894	*BOC*	3	113273112	T	C	C	0.029	0.019	0.039	9.04E−09	0.012	−0.010	0.033	2.98E−01
39	rs4073154	*H1FX-AS1*	3	129316642	A	G	G	0.038	0.027	0.049	2.71E−12	0.028	0.005	0.051	1.56E−02
40	rs7632556		3	134087102	A	G	G	0.030	0.020	0.041	1.37E−08	0.047	0.024	0.070	5.71E−05
41	rs57345461	*ZBTB38*	3	141407983	T	A	T	0.072	0.062	0.083	2.54E−42	0.070	0.047	0.092	1.04E−09
42	rs1104288	*RSRC1*	3	158249730	A	C	C	0.029	0.019	0.039	4.04E−08	0.008	−0.014	0.031	4.67E−01
43	rs12639337	*FNDC3B*	3	172279149	G	C	C	0.038	0.028	0.048	8.28E−14	0.038	0.016	0.060	5.45E−04
44	rs9790124	*RTP2, LOC100131635*	3	187712899	G	A	G	0.041	0.029	0.052	3.34E−12	0.023	−0.002	0.048	7.31E−02
45	rs116972792	*FAM184B*	4	17648794	A	G	G	0.080	0.052	0.108	1.53E−08	0.036	−0.027	0.099	2.62E−01
46	rs16895971	*LCORL*	4	17883363	C	T	T	0.102	0.091	0.113	3.69E−77	0.080	0.057	0.104	1.42E−11
47	rs16896140	*LCORL*	4	17957655	C	T	C	0.085	0.060	0.111	6.25E−11	0.063	0.008	0.118	2.41E−02
48	rs2724485	*LCORL*	4	17968075	C	T	T	0.028	0.018	0.038	3.41E−08	0.035	0.013	0.056	1.64E−03
49	rs148309730		4	18085973	G	A	A	0.093	0.064	0.122	3.64E−10	0.047	−0.014	0.108	1.31E−01
50	rs76924442		4	18118006	A	G	G	0.072	0.053	0.090	2.18E−14	0.031	−0.009	0.071	1.32E−01
51	rs4698216		4	18128100	T	C	T	0.045	0.035	0.055	2.43E−18	0.034	0.012	0.055	2.62E−03
52	rs56281640		4	56899650	A	G	A	0.030	0.020	0.040	3.41E−09	0.036	0.014	0.058	1.20E−03
53	rs10027494	*ADAMTS3*	4	72541929	A	T	A	0.033	0.022	0.045	1.94E−08	0.009	−0.016	0.035	4.72E−01
54	rs1662840		4	81235255	T	C	T	0.052	0.039	0.065	1.40E−15	0.060	0.032	0.088	2.06E−05
55	rs117072351	*HHIP*	4	144676679	T	C	T	0.091	0.060	0.121	4.51E−09	0.028	−0.038	0.095	4.04E−01
56	rs12654242		5	42365278	G	A	G	0.051	0.035	0.067	4.54E−10	0.062	0.027	0.097	4.64E−04
57	rs4273617	*GHR*	5	42695369	G	A	G	0.051	0.038	0.065	1.23E−13	0.035	0.006	0.064	1.80E−02
58	rs6453386	*SCAMP1*	5	78408512	C	G	C	0.033	0.022	0.044	3.80E−09	0.004	−0.020	0.028	7.24E−01
59	rs985296	*MEF2C-AS1*	5	89081827	A	G	G	0.034	0.024	0.044	8.81E−12	0.036	0.014	0.057	1.09E−03
60	rs184923695	*ARHGAP26*	5	143097754	A	G	G	0.054	0.036	0.072	4.62E−09	0.071	0.033	0.110	2.97E−04
61	rs186405009	*SLC17A1*	6	25823049	A	G	A	0.180	0.124	0.236	3.59E−10	0.099	−0.030	0.228	1.31E−01
62	rs811041		6	26225804	C	G	C	0.047	0.035	0.059	2.28E−15	0.050	0.025	0.075	1.05E−04
63	rs181680390	*BTN3A3*	6	26452046	A	T	A	0.126	0.081	0.171	4.06E−08	0.099	−0.003	0.201	5.67E−02
64	rs185780403		6	26745946	A	C	A	0.132	0.086	0.177	1.26E−08	0.100	−0.003	0.202	5.62E−02
65	rs192632187		6	27113326	C	T	C	0.137	0.091	0.182	3.75E−09	0.116	0.015	0.217	2.50E−02
66	rs183108303	*ZNF204P*	6	27370053	T	G	T	0.126	0.082	0.170	2.53E−08	0.117	0.017	0.218	2.15E−02
67	rs182819650		6	27823957	C	T	C	0.129	0.085	0.174	1.31E−08	0.108	0.008	0.208	3.48E−02
68	rs182706663		6	28116420	T	C	T	0.127	0.083	0.172	2.34E−08	0.094	−0.006	0.193	6.47E−02
69	rs2299870	*PPARD*	6	35417160	G	C	G	0.068	0.044	0.091	1.66E−08	0.033	−0.019	0.084	2.17E−01
70	rs1564926	*CD2AP*	6	47502410	C	T	C	0.030	0.020	0.041	2.70E−08	0.034	0.011	0.058	4.20E−03
71	rs145101575	*BCKDHB*	6	80344040	A	G	A	0.062	0.042	0.082	1.18E−09	0.053	0.009	0.096	1.79E−02
72	rs62424499		6	80678133	T	A	A	0.035	0.024	0.046	3.66E−10	0.042	0.018	0.065	5.78E−04
73	rs1145861		6	80940193	C	G	G	0.037	0.026	0.048	1.15E−10	0.041	0.016	0.065	1.15E−03
74	rs13197753		6	104924810	G	C	G	0.037	0.026	0.047	3.82E−12	0.047	0.024	0.069	4.91E−05
75	rs78638402		6	129995451	G	C	C	0.069	0.048	0.089	6.24E−11	0.029	−0.016	0.074	2.09E−01
76	rs6926186	*L3MBTL3*	6	130029149	G	A	G	0.053	0.034	0.072	2.11E−08	0.029	−0.011	0.070	1.51E−01
77	rs1040525	*ADGRG6*	6	142382532	T	C	C	0.044	0.034	0.054	7.17E−18	0.046	0.024	0.067	4.12E−05
78	rs73780873	*ESR1*	6	151829789	A	G	A	0.045	0.034	0.056	1.87E−15	0.018	−0.006	0.042	1.36E−01
79	rs1182176	*GNA12*	7	2834967	G	A	A	0.046	0.033	0.059	3.16E−12	0.063	0.035	0.090	1.18E−05
80	rs185053690	*KBTBD2*	7	32868716	T	G	G	0.158	0.111	0.206	6.20E−11	0.205	0.104	0.307	7.61E−05
81	rs2960429	*LOC102723446, LOC105375264*	7	46008459	G	C	C	0.035	0.025	0.045	4.87E−12	0.020	−0.001	0.042	6.40E−02
82	rs62452707		7	46530573	T	A	T	0.029	0.019	0.038	1.25E−08	0.027	0.006	0.049	1.20E−02
83	rs6557667	*LOXL2*	8	23390501	C	T	C	0.035	0.023	0.046	5.71E−09	0.013	−0.012	0.039	3.14E−01
84	rs74476179	*EXTL3*	8	28740152	A	G	G	0.111	0.078	0.145	9.56E−11	0.134	0.063	0.205	2.17E−04
85	rs10957084	*LOC105375821*	8	48444248	G	A	G	0.038	0.027	0.049	3.92E−11	0.022	−0.002	0.046	7.47E−02
86	rs181231559	*PLAG1*	8	56188663	C	T	T	0.185	0.123	0.246	3.55E−09	0.152	0.023	0.282	2.10E−02
87	rs6984782		8	56223330	C	T	T	0.081	0.062	0.099	2.42E−17	0.071	0.030	0.111	6.13E−04
88	rs112083368		8	56438778	G	C	C	0.061	0.042	0.081	7.16E−10	0.024	−0.018	0.066	2.68E−01
89	rs3886938	*GSDMC*	8	129725300	T	G	T	0.043	0.032	0.054	2.01E−14	0.046	0.022	0.070	1.33E−04
90	rs566313810		8	134043298	T	C	T	0.272	0.191	0.352	4.62E−11	0.305	0.114	0.496	1.73E−03
91	rs1213791479		8	134354346	C	T	C	0.283	0.206	0.359	5.41E−13	0.245	0.063	0.427	8.42E−03
92	rs368372931	*ZFAT*	8	134609000	G	A	G	0.139	0.094	0.184	1.51E−09	0.146	0.043	0.250	5.36E−03
93	rs1246647183	*ZFAT*	8	134627377	A	T	A	0.345	0.267	0.423	6.19E−18	0.307	0.119	0.494	1.38E−03
94	rs12541381	*ZFAT*	8	134637605	A	G	G	0.034	0.023	0.045	4.95E−10	0.059	0.036	0.082	6.14E−07
95	rs56119276		9	95518374	C	T	C	0.037	0.027	0.047	1.05E−12	0.023	0.001	0.045	4.50E−02
96	rs4743291		9	95758524	T	A	A	0.036	0.024	0.048	6.27E−09	0.024	−0.003	0.050	7.86E−02
97	rs10985794		9	122844338	C	T	T	0.035	0.023	0.048	2.68E−08	0.024	−0.003	0.051	8.51E−02
98	rs10901208	*FUBP3*	9	130587253	T	C	T	0.035	0.025	0.045	1.91E−12	0.031	0.010	0.052	4.40E−03
99	rs35859988	*CCDC3*	10	12902646	T	C	C	0.046	0.033	0.059	2.66E−12	0.033	0.005	0.061	2.28E−02
100	rs10998375	*TET1*	10	68665362	A	G	G	0.044	0.029	0.058	5.85E−09	0.042	0.010	0.073	9.99E−03
101	rs4979861		10	79371839	A	G	A	0.030	0.020	0.040	1.18E−08	0.011	−0.011	0.034	3.33E−01
102	rs291979	*GRK5*	10	119370285	A	G	A	0.035	0.023	0.047	1.43E−08	0.012	−0.014	0.039	3.66E−01
103	rs1003484	*IGF2, INS-IGF2*	11	2146388	G	A	G	0.036	0.026	0.046	7.41E−13	0.058	0.037	0.080	1.00E−07
104	rs78899385	*PSMA1*	11	14538479	T	C	C	0.081	0.062	0.101	2.17E−16	0.067	0.025	0.109	1.60E−03
105	rs76778262	*PDE3B*	11	14815707	C	A	A	0.083	0.057	0.108	1.67E−10	0.074	0.019	0.130	8.79E−03
106	rs4752839	*CELF1*	11	47473650	A	G	A	0.033	0.022	0.044	2.43E−09	0.058	0.034	0.081	1.72E−06
107	rs763648441	*LTBP3*	11	65546523	C	A	C	0.365	0.245	0.484	2.16E−09	0.299	0.051	0.548	1.82E−02
108	rs594318	*FOXRED1*	11	126277818	C	G	C	0.030	0.019	0.040	2.13E−08	0.028	0.006	0.051	1.37E−02
109	rs4763719	*ETV6*	12	11724486	G	A	A	0.030	0.020	0.040	3.28E−09	0.021	−0.001	0.042	5.78E−02
110	rs57454081		12	27950661	T	C	T	0.048	0.033	0.062	6.68E−11	0.027	−0.005	0.058	9.50E−02
111	rs76467375		12	46655632	T	C	T	0.040	0.026	0.054	1.43E−08	0.024	−0.006	0.054	1.20E−01
112	rs10444558		12	53661701	T	A	T	0.042	0.031	0.054	3.36E−13	0.054	0.029	0.078	1.84E−05
113	rs139121417	*RAB5B*	12	55986276	T	C	C	0.045	0.031	0.059	5.68E−10	0.055	0.024	0.086	4.71E−04
114	rs11834895	*HMGA2, HMGA2-AS1*	12	65853230	G	C	C	0.033	0.021	0.044	4.84E−08	0.018	−0.007	0.044	1.58E−01
115	rs151174669		12	65980466	T	C	C	0.069	0.046	0.092	7.72E−09	−0.004	−0.056	0.048	8.76E−01
116	rs7971647	*SOCS2*	12	93590078	C	T	C	0.046	0.036	0.056	1.10E−18	0.041	0.019	0.063	3.33E−04
117	rs80328976	*WASHC3*	12	102022590	C	G	G	0.058	0.047	0.069	3.78E−26	0.075	0.052	0.098	1.98E−10
118	rs1986854	*WASHC3*	12	102023697	C	T	C	0.043	0.027	0.058	3.00E−08	0.050	0.018	0.082	2.45E−03
119	rs12424129	*LINC02456*	12	102281461	C	T	T	0.060	0.049	0.070	9.80E−28	0.072	0.049	0.095	8.79E−10
120	rs12228148	*LINC02456*	12	102313697	A	G	A	0.050	0.037	0.063	5.00E−14	0.059	0.030	0.087	4.74E−05
121	rs17032833	*LOC105369944*	12	102561342	C	T	T	0.039	0.029	0.049	1.40E−14	0.025	0.003	0.046	2.42E−02
122	rs117988169	*HVCN1*	12	110672222	T	G	G	0.059	0.041	0.078	2.96E−10	0.011	−0.029	0.051	5.90E−01
123	rs3782886	*BRAP*	12	111672685	C	T	T	0.045	0.034	0.056	4.75E−16	0.019	−0.005	0.043	1.21E−01
124	rs116873087	*NAA25*	12	112074109	C	G	G	0.046	0.035	0.057	2.94E−16	0.016	−0.008	0.040	2.00E−01
125	rs11066359	*RPH3A*	12	112607850	T	C	C	0.029	0.019	0.039	1.39E−08	0.015	−0.007	0.037	1.70E−01
126	rs2072134	*OAS3*	12	112971371	A	G	G	0.039	0.026	0.052	2.44E−09	0.012	−0.016	0.040	4.01E−01
127	rs12590263	*TC2N*	14	91852154	A	G	A	0.033	0.023	0.042	7.92E−11	0.012	−0.010	0.033	2.80E−01
128	rs7143616	*ATXN3*	14	92065114	C	A	A	0.053	0.043	0.063	4.03E−24	0.041	0.019	0.063	3.09E−04
129	rs3759556		14	100725962	G	A	A	0.071	0.049	0.094	6.89E−10	0.073	0.024	0.123	3.66E−03
130	rs35443927		14	103383378	T	C	C	0.029	0.019	0.039	4.28E−08	0.026	0.004	0.049	2.07E−02
131	rs2663534		15	50883261	C	T	T	0.030	0.020	0.040	4.54E−09	0.027	0.005	0.049	1.41E−02
132	rs8041967	*MIR4713HG, CYP19A1*	15	51252127	A	G	A	0.039	0.029	0.049	1.75E−14	0.040	0.019	0.062	2.43E−04
133	rs28723025	*CYP19A1*	15	51304114	A	C	C	0.054	0.043	0.064	3.03E−22	0.054	0.031	0.078	5.60E−06
134	rs2162062	*VPS13C*	15	61987738	A	G	G	0.038	0.028	0.049	1.95E−13	0.014	−0.008	0.037	2.09E−01
135	rs2415130	*MYO9A*	15	71950213	A	G	A	0.028	0.018	0.038	2.08E−08	0.020	−0.002	0.041	7.45E−02
136	rs55763892	*PARP6*	15	72247839	T	C	C	0.032	0.021	0.043	3.00E−09	0.029	0.006	0.052	1.40E−02
137	rs6495171	*SIN3A*	15	75373282	A	G	A	0.031	0.020	0.042	2.70E−08	0.024	0.000	0.048	4.67E−02
138	rs1526080	*ADAMTSL3*	15	83921168	G	A	A	0.045	0.033	0.056	4.54E−15	0.024	−0.001	0.048	5.58E−02
139	rs938608	*ACAN*	15	88855374	T	G	G	0.041	0.029	0.053	5.26E−11	0.064	0.038	0.091	1.83E−06
140	rs138351276	*ACAN*	15	88859943	G	A	A	0.102	0.069	0.134	6.62E−10	0.169	0.096	0.241	5.54E−06
141	rs28456063		15	98637993	T	C	C	0.078	0.062	0.095	2.07E−20	0.102	0.066	0.138	3.09E−08
142	rs897377828	*IGF1R, IRAIN*	15	98649099	A	G	G	0.260	0.185	0.335	1.20E−11	0.157	0.001	0.313	4.80E−02
143	rs2573650	*ADAMTS17*	15	99973892	G	A	A	0.037	0.027	0.047	1.38E−13	0.047	0.025	0.068	1.87E−05
144	rs4619391	*WWP2*	16	69780860	T	A	T	0.030	0.019	0.040	9.78E−09	0.023	0.001	0.045	3.75E−02
145	rs114509338	*SF3B3*	16	70574733	C	T	T	0.033	0.021	0.044	1.72E−08	0.000	−0.024	0.025	9.74E−01
146	rs116560331	*POLR2A*	17	7488366	A	G	G	0.062	0.040	0.084	2.80E−08	0.049	0.002	0.095	4.04E−02
147	rs113934718	*ATAD5*	17	30887862	A	C	C	0.048	0.033	0.063	2.75E−10	0.024	−0.008	0.057	1.44E−01
148	rs67474242	*LRRC37A2, WNT3*	17	46777685	A	G	G	0.031	0.021	0.041	1.36E−09	0.000	−0.022	0.021	9.86E−01
149	rs2411374		17	48945636	C	T	C	0.037	0.026	0.047	1.49E−11	0.021	−0.002	0.044	7.40E−02
150	rs6504608	*ZNF652*	17	49347319	A	C	C	0.030	0.020	0.041	1.41E−08	0.014	−0.009	0.037	2.30E−01
151	rs9905385		17	61420889	A	G	A	0.053	0.042	0.064	1.08E−21	0.046	0.022	0.069	1.67E−04
152	rs2320125	*CD79B*	17	63930958	C	T	C	0.046	0.036	0.056	4.63E−20	0.040	0.019	0.061	2.11E−04
153	rs11651289		17	63936114	T	C	T	0.070	0.047	0.094	3.45E−09	0.073	0.024	0.121	3.41E−03
154	rs4239437	*CABLES1*	18	23152260	T	C	C	0.074	0.061	0.087	5.92E−29	0.053	0.025	0.082	2.24E−04
155	rs9807648	*TMEM241*	18	23344958	G	A	G	0.033	0.022	0.045	3.28E−08	0.030	0.004	0.056	2.16E−02
156	rs4349223	*FHOD3*	18	36541412	A	C	C	0.029	0.019	0.039	6.93E−09	0.021	−0.001	0.042	5.91E−02
157	rs12606199	*DYM, LOC100129878*	18	49045546	A	G	G	0.046	0.034	0.057	3.49E−15	0.023	−0.002	0.048	7.04E−02
158	rs201707253	*DYM*	18	49342419	T	G	G	0.042	0.031	0.052	6.17E−14	0.017	−0.006	0.041	1.53E−01
159	rs3843750	*SLC44A2*	19	10637397	C	G	C	0.039	0.029	0.050	1.55E−13	0.039	0.016	0.061	8.68E−04
160	rs754332	*KIZ, KIZ-AS1*	20	21197259	A	G	A	0.029	0.019	0.039	1.28E−08	0.018	−0.004	0.040	1.04E−01
161	rs61016611		20	35624229	A	G	A	0.062	0.043	0.081	1.23E−10	0.058	0.017	0.099	5.31E−03
162	rs3827030	*PHF20*	20	35887025	G	A	G	0.060	0.047	0.074	1.87E−19	0.042	0.014	0.071	3.47E−03
163	rs8183892		20	36980155	T	C	T	0.041	0.031	0.051	2.79E−16	0.028	0.006	0.049	1.18E−02
164	rs4608	*RPN2*	20	37236651	T	C	T	0.044	0.034	0.054	2.23E−18	0.026	0.005	0.048	1.61E−02
165	rs8121252	*GNAS*	20	58901754	T	C	C	0.048	0.037	0.059	2.30E−17	0.053	0.029	0.077	1.42E−05
166	rs5754190	*SYN3, LOC105373002*	22	32654480	C	T	C	0.052	0.042	0.063	4.32E−22	0.050	0.027	0.073	1.90E−05
167	rs4821086	*SYN3*	22	32687867	A	C	A	0.077	0.052	0.102	2.10E−09	0.039	−0.015	0.093	1.59E−01
168	rs7290267	*MIRLET7BHG*	22	46088855	G	A	G	0.052	0.037	0.068	1.75E−11	0.062	0.029	0.095	2.30E−04

*SNP*, single nucleotide polymorphism; *No.*, number; *Chr.*, chromosome; *95% CI*, 95% confidence interval; *GWAS*, genome-wide association study

This analysis was performed under the additive inheritance model. These SNPs were ordered by chromosome and position. Positions were based on the NCBI GRCh38 version. Genes were identified based on the gene containing the SNP or the nearest gene (within 100 kb up- or downstream) to the SNP

The measured (phenotypic) heights (cm) were stratified by sex, mean-centered, and normalized to one standard deviation (SD) before height GWAS analysis

Beta-value calculation was conducted according to the defined risk alleles

**Table 2:** Association of previously reported GWAS height SNPs with height in Taiwan

No.	rs ID	Gene	Chr.	Position	Minor allele	Major allele	Risk allele	Training group (***N*** = 67,452) (***p*** < 0.05/1722)				Testing group (***N*** = 14,454)			
								Beta	95% CI		***P***-value	Beta	95% CI		***P***-value
1	rs2300092	*MTOR*	1	11206407	T	C	T	0.027	0.014	0.039	2.62E−05	0.024	−0.003	0.050	8.51E−02
2	rs3014240	*CCDC17*	1	45623553	C	G	C	0.025	0.015	0.036	9.06E−07	0.032	0.010	0.054	4.17E−03
3	rs2666504		1	62169409	C	T	C	0.024	0.013	0.034	5.80E−06	0.015	−0.007	0.037	1.84E−01
4	rs11205303	*MTMR11*	1	149934520	C	T	C	0.057	0.047	0.067	5.69E−28	0.033	0.011	0.055	3.26E−03
5	rs6587515		1	150636412	A	G	A	0.026	0.014	0.037	2.27E−05	0.018	−0.008	0.043	1.71E−01
6	rs1325596	*PAPPA2*	1	176824930	G	A	A	0.032	0.021	0.044	6.10E−08	0.043	0.018	0.068	9.05E−04
7	rs10911212	*LAMC1*	1	183055334	C	T	C	0.024	0.014	0.034	2.83E−06	0.007	−0.015	0.029	5.30E−01
8	rs4472734	*PTPN14*	1	214444842	C	T	C	0.028	0.018	0.037	4.96E−08	0.013	−0.009	0.034	2.48E−01
9	rs10165255	*CYS1*	2	10059474	A	G	A	0.030	0.016	0.044	1.70E−05	0.037	0.008	0.066	1.30E−02
10	rs6735681		2	15983051	T	C	T	0.023	0.013	0.033	5.34E−06	0.014	−0.007	0.036	1.98E−01
11	rs780094	*GCKR*	2	27518370	T	C	C	0.022	0.012	0.031	1.69E−05	0.025	0.004	0.046	2.22E−02
12	rs3755206	*CRIM1*	2	36456285	G	T	T	0.054	0.042	0.067	7.94E−17	0.054	0.026	0.081	1.35E−04
13	rs6544743	*LOC102723904*	2	44163230	T	G	T	0.024	0.013	0.035	1.52E−05	0.005	−0.018	0.029	6.55E−01
14	rs3791675	*EFEMP1*	2	55884174	C	T	C	0.075	0.064	0.087	2.78E−37	0.072	0.047	0.097	2.06E−08
15	rs1432559	*LOC105374690*	2	55962483	G	T	G	0.055	0.030	0.080	1.32E−05	0.073	0.019	0.126	8.21E−03
16	rs4241349	*ANTXR1*	2	69103152	G	A	G	0.026	0.014	0.037	9.55E−06	0.039	0.015	0.064	1.78E−03
17	rs76709099	*IHH*	2	219055182	A	C	C	0.064	0.047	0.080	8.72E−14	0.061	0.025	0.097	9.16E−04
18	rs2564923		3	53069246	A	G	A	0.027	0.016	0.038	1.49E−06	0.034	0.011	0.058	4.25E−03
19	rs9841212		3	134473096	C	T	T	0.024	0.013	0.036	2.79E−05	0.003	−0.021	0.027	8.10E−01
20	rs1055153	*WWTR1*	3	149657086	T	G	G	0.046	0.030	0.062	1.33E−08	0.013	−0.022	0.047	4.69E−01
21	rs6774762	*GHSR*	3	172447200	G	A	A	0.037	0.024	0.050	1.80E−08	0.032	0.005	0.060	2.16E−02
22	rs7697556		4	72649596	C	T	T	0.037	0.027	0.047	1.77E−13	0.030	0.009	0.051	5.37E−03
23	rs17017911	*GUSBP5*	4	143559481	G	A	A	0.022	0.012	0.032	2.19E−05	0.016	−0.005	0.038	1.40E−01
24	rs6845999	*HHIP-AS1*	4	144644674	T	C	T	0.058	0.046	0.070	7.29E−22	0.062	0.037	0.088	1.52E−06
25	rs4240326		4	144918112	A	G	A	0.028	0.017	0.039	7.17E−07	0.018	−0.006	0.042	1.50E−01
26	rs301901	*NIPBL*	5	37046524	A	G	A	0.024	0.014	0.034	2.04E−06	0.016	−0.005	0.038	1.32E−01
27	rs4865956		5	55586677	T	A	T	0.024	0.014	0.035	5.07E−06	0.018	−0.005	0.040	1.22E−01
28	rs7706662	*CEP120*	5	123419868	T	C	C	0.023	0.013	0.032	7.16E−06	−0.014	−0.035	0.008	2.09E−01
29	rs2908532		5	142242319	A	C	A	0.027	0.016	0.037	1.23E−06	0.010	−0.013	0.033	3.93E−01
30	rs2974438	*SLIT3*	5	168823898	A	G	G	0.030	0.017	0.043	3.53E−06	0.020	−0.008	0.047	1.61E−01
31	rs12153391	*SMIM23*	5	171776434	A	C	C	0.029	0.019	0.039	1.22E−08	0.016	−0.006	0.038	1.46E−01
32	rs4868126		5	171856465	T	G	G	0.031	0.020	0.041	4.13E−09	0.041	0.019	0.063	2.39E−04
33	rs722585	*GMDS, HCG17*	6	1775629	A	G	G	0.023	0.013	0.034	1.61E−05	0.060	0.037	0.083	3.26E−07
34	rs78566116		6	32428369	T	G	G	0.040	0.022	0.057	5.70E−06	0.039	0.002	0.075	3.87E−02
35	rs2780226		6	34231315	C	T	C	0.095	0.079	0.110	1.75E−32	0.072	0.038	0.106	2.87E−05
36	rs12209223	*FILIP1, LOC101928540*	6	75454873	A	C	A	0.050	0.033	0.067	1.02E−08	0.057	0.020	0.095	2.76E−03
37	rs648831	*BCKDHB*	6	80246491	C	T	T	0.024	0.014	0.034	1.53E−06	0.017	−0.004	0.038	1.22E−01
38	rs3805859	*BCKDHB*	6	80339229	C	A	C	0.025	0.015	0.035	5.62E−07	0.017	−0.005	0.038	1.23E−01
39	rs113898003	*L3MBTL3*	6	130020090	C	T	T	0.042	0.032	0.052	4.72E−16	0.045	0.023	0.068	5.45E−05
40	rs7765757	*EPB41L2*	6	131050608	C	T	T	0.038	0.022	0.053	1.29E−06	0.038	0.006	0.070	2.12E−02
41	rs3020359	*ESR1*	6	152044128	T	C	C	0.023	0.013	0.033	1.11E−05	0.035	0.013	0.057	1.78E−03
42	rs73029259	*LOC107986666*	6	163690316	A	T	A	0.037	0.020	0.054	1.99E−05	0.033	−0.004	0.069	7.95E−02
43	rs57246313		7	25850077	A	G	A	0.024	0.014	0.034	3.01E−06	0.026	0.004	0.048	1.93E−02
44	rs1007358		7	46161757	G	A	G	0.036	0.023	0.049	1.12E−07	0.041	0.012	0.069	5.43E−03
45	rs42377	*CDK6*	7	92614358	A	G	A	0.035	0.019	0.052	2.44E−05	0.007	−0.029	0.042	7.15E−01
46	rs445	*CDK6*	7	92779056	T	C	C	0.025	0.015	0.035	1.86E−06	0.029	0.007	0.050	1.08E−02
47	rs76364830	*DLC1*	8	13514611	A	G	G	0.041	0.023	0.059	9.41E−06	0.059	0.019	0.098	3.93E−03
48	rs10958476	*PLAG1*	8	56183249	C	T	C	0.030	0.017	0.042	2.60E−06	0.014	−0.013	0.041	3.04E−01
49	rs7842996		8	77194904	A	T	A	0.031	0.019	0.043	6.50E−07	0.033	0.007	0.060	1.47E−02
50	rs7817087		8	116552698	A	G	G	0.026	0.016	0.035	4.15E−07	0.010	−0.012	0.031	3.81E−01
51	rs6992491		8	128185657	G	C	G	0.022	0.012	0.032	1.68E−05	0.011	−0.011	0.033	3.22E−01
52	rs10120219	*LOC105376158*	9	95602265	C	T	T	0.036	0.026	0.046	7.29E−13	0.040	0.018	0.061	2.77E−04
53	rs34575265		9	106181520	T	C	C	0.022	0.012	0.032	2.66E−05	0.011	−0.011	0.033	3.16E−01
54	rs7858562	*ZNF483, PTGR1*	9	111562668	G	A	A	0.025	0.014	0.037	2.33E−05	0.035	0.009	0.060	7.19E−03
55	rs12344818		9	115728289	T	C	C	0.031	0.019	0.043	1.73E−07	−0.006	−0.031	0.020	6.65E−01
56	rs3789280	*PAPPA*	9	116191093	A	T	A	0.036	0.020	0.053	1.23E−05	0.018	−0.018	0.053	3.30E−01
57	rs12338076	*QSOX2*	9	136229894	C	A	C	0.040	0.029	0.050	3.63E−13	0.045	0.022	0.068	1.50E−04
58	rs779933	*ZMIZ1*	10	79158760	A	G	G	0.028	0.017	0.039	8.37E−07	0.038	0.014	0.062	2.22E−03
59	rs2648725	*PCGF5*	10	91255322	A	T	A	0.044	0.024	0.064	1.61E−05	0.052	0.009	0.095	1.77E−02
60	rs1938679		11	69457328	T	C	C	0.038	0.029	0.048	2.98E−14	0.032	0.010	0.053	3.84E−03
61	rs645935	*SERPINH1*	11	75568245	C	T	T	0.042	0.032	0.051	9.90E−17	0.034	0.012	0.055	2.03E−03
62	rs59917308		12	56264924	T	C	T	0.069	0.039	0.099	5.37E−06	−0.005	−0.069	0.059	8.81E−01
63	rs3816804	*CS*	12	56286961	T	C	C	0.112	0.099	0.126	6.35E−63	0.114	0.085	0.142	3.14E−15
64	rs2277339	*PRIM1*	12	56752285	G	T	T	0.037	0.025	0.049	1.81E−09	0.040	0.014	0.066	2.91E−03
65	rs10747784		12	57857579	G	A	G	0.027	0.015	0.038	4.71E−06	0.019	−0.005	0.044	1.21E−01
66	rs10878984		12	69434754	C	T	T	0.044	0.034	0.054	5.99E−17	0.025	0.003	0.047	2.84E−02
67	rs3847787	*CRADD*	12	93813756	G	A	G	0.022	0.012	0.033	1.91E−05	0.031	0.009	0.053	6.20E−03
68	rs2093210	*C14orf39*	14	60490561	T	C	C	0.043	0.031	0.055	2.54E−12	0.027	0.001	0.053	4.52E−02
69	rs910316	*TMED10*	14	75159339	A	C	A	0.029	0.016	0.042	1.20E−05	0.030	0.002	0.058	3.67E−02
70	rs7156335	*ITPK1*	14	92939887	C	T	C	0.049	0.028	0.070	4.46E−06	0.033	−0.013	0.080	1.56E−01
71	rs12592845		15	48392761	T	C	C	0.028	0.015	0.041	1.99E−05	0.065	0.037	0.093	5.11E−06
72	rs975210	*TLE3*	15	70072013	A	G	A	0.033	0.019	0.048	7.70E−06	0.004	−0.028	0.036	8.25E−01
73	rs750460	*LOXL1*	15	73949165	A	G	G	0.047	0.031	0.063	1.16E−08	0.048	0.012	0.083	8.87E−03
74	rs8025068	*ARID3B*	15	74577704	G	T	G	0.023	0.013	0.032	6.66E−06	0.028	0.006	0.049	1.10E−02
75	rs4467054	*ADAMTS17*	15	100255167	G	T	G	0.029	0.018	0.039	9.87E−08	0.038	0.014	0.061	1.45E−03
76	rs258324	*CDK10*	16	89687847	T	G	T	0.043	0.032	0.054	1.97E−15	0.046	0.023	0.069	1.13E−04
77	rs74494415	*GALR1*	18	77260182	T	C	C	0.040	0.022	0.058	8.97E−06	0.026	−0.013	0.064	1.88E−01
78	rs1741344		20	4121153	C	T	C	0.032	0.019	0.044	3.37E−07	0.040	0.014	0.067	2.99E−03
79	rs967417		20	6640246	G	A	G	0.033	0.020	0.046	1.10E−06	0.040	0.011	0.068	5.95E−03
80	rs3213180	*E2F1*	20	33675818	C	G	G	0.032	0.022	0.043	1.83E−09	0.043	0.020	0.066	2.71E−04
81	rs143384	*GDF5*	20	35437976	G	A	G	0.074	0.063	0.085	3.61E−40	0.056	0.032	0.079	4.19E−06
82	rs2235363	*ZHX3*	20	41179129	G	A	G	0.023	0.013	0.032	7.22E−06	0.011	−0.010	0.032	3.10E−01
83	rs11537645	*UBE2C*	20	45812764	G	C	C	0.044	0.026	0.061	1.45E−06	0.008	−0.031	0.046	7.01E−01

*SNP*, single nucleotide polymorphism; *GWAS*, genome-wide association study; *No*., number; *Chr*., chromosome; *95% CI*, 95% confidence interval

The measured (phenotypic) heights (cm) were stratified by sex, mean-centered, and normalized to one standard deviation (SD) before height GWAS analysis

Beta-value calculation was performed in agreement with the defined risk alleles

### Association between the genetically determined height (PRS_251_) and the measured height (phenotype)

The association between genetically determined height (PRS_251_) and measured height (phenotype) was investigated in the testing and validation groups, where height was stratified by sex (male: *N* = 10,919; female: *N* = 17,990; Fig. 4). For males, the regression line indicated that a 1-SD increase in PRS_251_ was associated with a 0.257-SD increase in normalized measured height (slope = 0.257; *p* < 0.001; green line). For females, the regression line indicated that a 1-SD increase in PRS_251_ was associated with a 0.274-SD increase in normalized measured height (slope = 0.274; *p* < 0.001; red line).

Furthermore, to assess the validity of our findings, we replicated the association between genetically determined height (PRS_237_) and measured heights (phenotype) in another cohort, kindly provided by the Big Data Center in China Medical University Hospital (CMUH), Taichung, Taiwan (Additional file 4: Fig. S3). As shown, only 237 of the 251 SNPs were available from the independent cohort of the Big Data Center at CMUH (Additional file 5: Table S4). The measured height (phenotype) and genetically determined height (PRS_237_) were normalized (standardized) by sex. For males, the regression line indicated that a 1-SD increase in PRS_237_ was associated with a 0.0972-SD increase in normalized measured height (slope = 0.0972; *p* < 0.001; green line; Additional file 4: Fig. S3). For females, the regression line indicated that a 1-SD increase in (PRS_237_) was associated with a 0.104-SD increase in normalized measured height (slope = 0.104; *p* < 0.001; red line; Additional file 4: Fig. S3).

### Associations of height with health-related outcomes

**Fig. 5:**
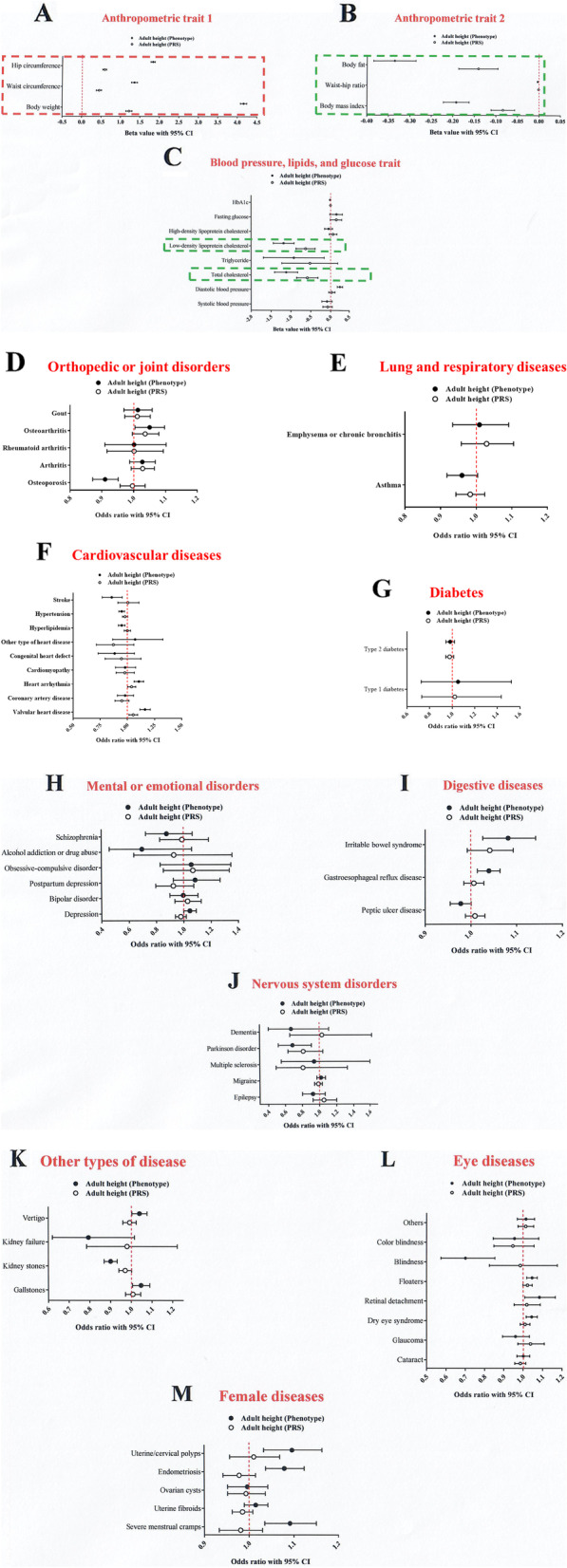
Observational (phenotype) and genetic PRS_251_ associations of height with 63 health-related outcomes. Beta value and 95% confidence interval (CI) per standard deviation (SD) increase in height are shown for **A** anthropometric trait 1 (hip circumference, waist circumference, and body weight), **B** anthropometric trait 2 (body fat, waist-hip ratio, and body mass index), and **C** blood pressure, blood lipid level, and blood glucose level (including systolic blood pressure (SBP), diastolic blood pressure (DBP), total cholesterol (TC), triglyceride (TG), low-density lipoprotein cholesterol (LDL-C), high-density lipoprotein cholesterol (HDL-C), fasting glucose, and HbA1c). Odds ratio and 95% CI per SD increase in height are shown for **D** orthopedic or joint disorders (osteoporosis, arthritis, rheumatoid arthritis, osteoarthritis, and gout), **E** lung and respiratory diseases (asthma and emphysema or chronic bronchitis), **F** cardiovascular diseases (valvular heart disease, coronary artery disease, heart arrhythmia, cardiomyopathy, congenital heart defect, other type of heart disease, hyperlipidemia, hypertension, and stroke), **G** diabetes (type 1 diabetes and type 2 diabetes), **H** mental or emotional disorders (depression, bipolar disorder, postpartum depression, obsessive-compulsive disorder, alcohol addiction or drug abuse, and schizophrenia), **I** digestive diseases (peptic ulcer disease, gastroesophageal reflux disease, and irritable bowel syndrome), **J** nervous system disorders (epilepsy, migraine, multiple sclerosis, Parkinson’s disorder, and dementia), **K** other types of disease (gallstones, kidney stones, kidney failure, and vertigo), **L** eye diseases (cataract, glaucoma, dry eye syndrome, retinal detachment, floaters, blindness, color blindness, and others), and **M** female diseases (severe menstrual cramps, uterine fibroids, ovarian cysts, endometriosis, and Uterine/cervical polyps)

In this study, we performed both observational (phenotype) and genetic PRS association analyses of height with 63 health-related outcomes using the Taiwan Biobank (Fig. 5). We examined the association between observational (phenotype) height with 63 health-related outcomes, including 14 traits and 49 diseases, in 67,452 individuals of Han Chinese ancestry (Fig. 5). Similar analyses of the association between genetic PRS of height and height were performed (Fig. 5). The genetically determined height of PRS_251_ (251 SNPs) applied in this analysis was calculated from our GWAS results, consisting of 168 GWAS-identified SNPs (Table 1) and 83 previously reported GWAS-determined SNPs (Table 2). The estimated beta values (95% CI) for the 14 traits are shown in Fig. 5A–C. The estimated odds ratios (95% CI) for the 49 diseases are also shown in Fig. 5D–M. After adjusting for age, sex, education, drinking, smoking, regular exercise, and 10 PCA results, our analyses showed that observational (phenotype) height was associated with eight of the 14 traits (*p* < 0.05/[14 + 49]; Table 3). Further analyses confirmed that genetic (PRS_251_) height was also associated with these eight traits (Table 3). No significant associations were observed between the measured and genetic PRS height with the 49 diseases (*p* > 0.05/[14 + 49]; Fig. 5D–M). Among anthropometric traits, observational height was positively associated with body weight, waist circumference, and hip circumference but negatively associated with BMI, WHR, and body fat (Table 3). Genetic PRS height was associated with increased body weight (beta = 1.2182, 95% CI = 1.1405–1.2959), waist circumference (beta = 0.4462, 95% CI = 0.3754–0.5171), and hip circumference (beta = 0.6006, 95% CI = 0.5488–0.6523), and a decreased BMI (beta = −0.0837, 95% CI = (−0.1110)–(−0.0563)), WHR (beta = −0.0008, 95% CI = (−0.0012)–(−0.0003)), and body fat (beta = −0.1401, 95% CI = (−0.1856)–(−0.0946)).

**Table 3:** Significant association between phenotypic and genetically determined height with eight traits

Height (exposure)	8 traits (outcome)	Beta	95% confidence interval		***P***-value
	**Anthropometric trait 1**				
Height (Phenotype)	Body weight (Phenotype)	4.162	4.083	4.241	***0.00E+00***
Height (PRS)	Body weight (Phenotype)	1.218	1.141	1.296	***7.81E−206***
Height (Phenotype)	Waist circumference (Phenotype)	1.363	1.287	1.440	***1.56E−264***
Height (PRS)	Waist circumference (Phenotype)	0.446	0.375	0.517	***5.81E−35***
Height (Phenotype)	Hip circumference (Phenotype)	1.845	1.790	1.900	***0.00E+00***
Height (PRS)	Hip circumference (Phenotype)	0.601	0.549	0.652	***4.53E−114***
	**Anthropometric trait 2**				
Height (Phenotype)	Body mass index (Phenotype)	−0.192	−0.222	−0.162	***1.36E−36***
Height (PRS)	Body mass index (Phenotype)	−0.084	−0.111	-0.056	***2.01E−09***
Height (Phenotype)	Waist-hip ratio (Phenotype)	−0.003	−0.003	−0.002	***1.75E−24***
Height (PRS)	Waist-hip ratio (Phenotype)	−0.001	−0.001	0.000	***7.18E−04***
Height (Phenotype)	Body fat (Phenotype)	−0.335	−0.385	−0.286	***3.82E−40***
Height (PRS)	Body fat (Phenotype)	−0.140	−0.186	−0.095	***1.58E−09***
	**Blood pressure, lipids, and glucose trait**				
Height (Phenotype)	Total cholesterol (Phenotype)	−1.117	−1.407	−0.828	***4.13E−14***
Height (PRS)	Total cholesterol (Phenotype)	−0.587	−0.853	−0.321	***1.55E−05***
Height (Phenotype)	Low-density lipoprotein cholesterol (Phenotype)	−1.180	−1.439	−0.921	***4.68E−19***
Height (PRS)	Low-density lipoprotein cholesterol (Phenotype)	−0.629	−0.867	−0.391	***2.25E−07***

*PRS* polygenic risk score

Height phenotype indicates the measured height (normalized using *Z*-scores)

Height polygenic risk score (PRS) indicates the height calculated from 251 SNPs (Tables 1 and 2)

Multivariate linear regression analysis was used with adjustment factors (age, sex, educational attainment, drinking, smoking, regular exercise, and 10 principal components analysis data). *P*-value (*p* < 0.05/(14+49)) was highlighted in bold italic. *P*-value less than *2.23E−308* were expressed as *0.00E+00*

Regarding blood pressure, and lipid and glucose levels, observational height was negatively associated with TC and LDL-C (Table 3). Genetic PRS height was associated with decreased TC (beta = −0.5869, 95% CI = (−0.8530)–(−0.3207)) and LDL-C levels (beta = −0.6291, 95% CI = (−0.8672)–(−0.3910)).

## Discussion

We reported a genetic profile for height in the Han Chinese population using genome-wide SNP analysis and a replication study in the Taiwan Biobank—a community-based database in Taiwan. This is the first large-scale finding on the genetic basis for height and health-related outcomes in individuals of Han Chinese ancestry in Taiwan. Our study results are consistent with the genetic profile of height observed mainly in individuals of European ancestry [15–26]. Accordingly, our findings support the validity of height in observational (phenotype) studies and are consistent with the health-related outcomes of this phenotype [73–81].

In this study, we identified 6843 SNPs with genome-wide significance in 89 genomic regions, including 18 novel loci. Among these, we identified seven independent lead SNPs at seven genetic loci (two of these lead SNPs were novel: chromosome 2, rs76803230 in *DIS3L2*; chromosome 3, rs57345461 in *ZBTB38*) with genome-wide significance. *DIS3L2* encodes one of the subunits of the RNA exosome, and its genetic variants are associated with height in individuals of European ancestry [21, 82] and East Asian ancestry [83, 84]. *ZBTB38* is a zinc finger transcriptional activator that binds methylated DNA and is associated with apoptosis. *ZBTB38* genetic variants are associated with height in individuals of European ancestry [17, 18] and East Asian ancestry [20, 23]. The remaining five lead SNPs were reported previously [15, 23, 64–72]. The lead SNP rs3791675 in *EFEMP1* encodes an extracellular matrix glycoprotein of the fibulin family and has been associated with body height, BMI-adjusted waist circumference, pelvic organ prolapse, and BMI-adjusted WHR [23, 64–66]. The lead SNP rs16895971 in *LCORL*, a transcription factor involved in spermatogenesis, has been associated with height in East Asians [67]. The lead SNP rs2780226 in *HMGA1* encodes a chromatin-associated protein that regulates gene transcription and metastatic progression of cancer cells and has been associated with body height, BMI-adjusted waist circumference, and birth weight [15, 68, 69]. The lead SNP rs3816804 in *CS* has also been associated with height in East Asians [70]. The lead SNP rs143384 in *GDF5*, which encodes a secreted ligand of the transforming growth factor-beta superfamily, regulates the development of numerous tissue and cell types and has been associated with body height, BMI-adjusted hip circumference, BMI-adjusted WHR, and body fat [15, 64, 71, 72]. Our observations report novel lead SNPs in the Han Chinese population that share an overlapping genetic architecture for height, mainly discovered in individuals of European ancestry.

Our observational (phenotype) analyses showed that height was associated with eight traits. Furthermore, our PRS_251_ analyses confirmed that genetic height was also associated with these eight traits. Taller height was associated with decreased BMI, WHR, body fat, TC, and LDL-C, but with increased body weight, waist circumference, and hip circumference. For anthropometric traits, we observed that both observational (phenotype) and genetically determined height (PRS_251_) were associated with BMI, WHR, body fat, body weight, waist circumference, and hip circumference. Taller height was associated with decreased BMI, WHR, and body fat, but increased body weight, waist circumference, and hip circumference. Our findings are consistent with previous observational (phenotype) studies that reported an inverse association between height and obesity-related traits, including BMI, WHR, and body fat [73, 74]. As expected, taller adults had lower rates of obesity [73, 74]. Our findings also support previous observational (phenotype) studies that reported positive associations between height and body weight [73–75], waist circumference [74, 75], and hip circumference [75]. Furthermore, taller adults had increased body weight and waist and hip circumferences. The results of our genetic PRS of height association were also in agreement with previous genetic correlation studies, mainly conducted in individuals of European ancestry [78–81]. There was a negative correlation between genetically determined height and BMI [78–80], and positive correlations between genetically determined height with waist and hip circumference [80, 81]. Our findings may reflect a partial genetic overlap between height and anthropometric traits including BMI, WHR, body fat, body weight, waist circumference, and hip circumference. However, genetic correlations in individuals of Han Chinese ancestry remain to be elucidated.

Regarding blood pressure and lipids and glucose levels, both observational (phenotype) and genetically determined height (PRS_251_) were inversely associated with TC and LDL-C. Our findings are consistent with those of previous observational (phenotype) studies that reported an inverse association between height and blood lipid levels [76, 77, 85]. Taller adults have lower levels of TC and LDL-C [76, 77, 85]. The results of our genetic PRS of height are also in agreement with previous studies, mainly conducted in individuals of European ancestry [50, 80, 81]. Negative genetic correlations between height and TC were found [80, 81]. A taller genetic PRS was associated with lower LDL-C levels in individuals of European ancestry [50]. Our results also suggest that genetically taller individuals of Han Chinese ancestry have lower levels of TC and LDL-C.

## Conclusions

This large-scale assessment of the genetic architecture of height in the Han Chinese population of Taiwan quantified the extent of the shared genetic basis with individuals of European ancestry. Our observational and genetic study supports the relevance of height to the etiology of various health-related outcomes, especially those regarding anthropometric traits and blood lipids.

## Supplementary Information

**Additional file 1: Table S1**. Basic characteristics of the study participants at enrollment.

**Additional file 2: Table S2**. The top lead SNPs in 89 genomic regions that were significantly associated with height (*p* < 5 × 10^−8^), using LD (r^2^ < 0.2), in individuals of Han Chinese ancestry.

**Additional file 3: Table S3**. Replication of a previous GWAS of body height in the SNPs of the training group (313 of 1722 SNPs).

**Additional file 4: Figure S1**. The clumping and *p*-value threshold method identifies the “best-fit” SNP number for the polygenic risk score (PRS) calculation, according to the largest explainable phenotype correlation r^2^ using only PRS (PRS r^2^ and SNP number). The x-axis shows the *p*-value thresholds from the height of GWAS results. The y-axis represents the explainable phenotypic correlation r^2^ using only the PRS (PRS r^2^). The *p*-values above the bars show the statistical significance of the associations between genetically determined height (PRS) and measured height (phenotype). (A) 251 SNPs were obtained from 6,941 SNPs (novel and reported SNPs; PRS r^2^ = 0.0712, SNP number = 251). (B) 194 SNPs were obtained from 6,843 SNPs (novel SNPs; PRS r^2^ = 0.0622, SNP number = 194). (C) 154 SNPs were obtained from 313 SNPs (reported SNPs; PRS r^2^ = 0.0706, SNP number = 154). **Figure S2**. Scree plot identifying the number of principal component analyses (PCA) needed for the correction of population structure in the height GWAS study, using pcadapt (an R package used to determine the number of principal components). **Figure S3**. Association between genetically determined height (PRS_237_) and measured height (phenotype) in an independent cohort of the Big Data Center in China Medical University Hospital in Taiwan. The measured height (cm) and calculated polygenic risk score (PRS) for height were stratified by sex, mean-centered, and normalized to one standard deviation (SD; males, *N* = 46,310; females, *N* = 54,728). The normalized measured height is represented on the y-axis, and normalized genetically determined height (PRS_237_) is represented on the x-axis.

**Additional file 5: Table S4**. Characteristics of 237 out of the 251 SNPs associated with height in the independent cohort of the Big Data Center at China Medical University Hospital in Taiwan.

## Data Availability

Individual-level Taiwan Biobank data are available upon application to the Taiwan Biobank (https://www.twbiobank.org.tw/new_web/).

## Abbreviations

BMI: Body mass index
CI: Confidence interval
CS: Citrate synthase
CVD: Cardiovascular disease
DBP: Diastolic blood pressure
DIS3L2: DIS3 like 3′-5′ exoribonuclease 2
EFEMP1: EGF-containing fibulin extracellular matrix protein 1
GDF5: Growth differentiation factor 5
GWAS: Genome-wide association study
HDL-C: High-density lipoprotein cholesterol
HMGA1: High-mobility group AT-hook 1
IBD: Individual identity by descent
LCORL: Ligand-dependent nuclear receptor corepressor like
LD: Linkage disequilibrium
LDL-C: Low-density lipoprotein cholesterol
LM: Linear regression model
MAF: Minor allele frequency
PCA: Principal component analysis
PRS: Polygenic risk score
QC: Quality control
SBP: Systolic blood pressure
SD: Standard deviation
SNP: Single nucleotide polymorphism
TC: Total cholesterol
TG: Triglyceride
WHR: Waist-hip ratio
ZBTB38: Zinc finger and BTB domain containing 38
