# Supplementary material for: Your height affects your health: genetic determinants and health-related outcomes in Taiwan
Source: BMC Med. 2022 Jul 13;20:250. doi: 10.1186/s12916-022-02450-w (PMC9281111; doi:10.1186/s12916-022-02450-w)
Supplement: Supplementary file 1 — Additional file 1: Table S1. Basic characteristics of the study participants at enrollment. [file 12916_2022_2450_MOESM1_ESM.docx]

| **Additional file 1: Table S1** Basic characteristics of the study participants at enrollment | | | | |
| --- | --- | --- | --- | --- |
|  | **Training group** |  | **Testing group** | ***P* value** |
|  | **N = 67,452** |  | **N = 14,454** |  |
|  | **N (%)** |  | **N (%)** |  |
| **Age (year), mean (SD)** | 50.07 (10.73) |  | 49.98 (10.79) | 0.391 |
| **Gender (Female / male ratio)** | 1.63 (0.48) |  | 1.63 (0.48) | 0.950 |
| **Anthropometric measurements** |  |  |  |  |
| Height (cm), mean (SD) | 162.10 (8.29) |  | 161.98 (8.25) | 0.118 |
| Body weight (kilogram), mean (SD) | 63.98 (12.67) |  | 63.99 (12.91) | 0.954 |
| Waist circumference (cm), mean (SD) | 83.40 (10.16) |  | 83.40 (10.35) | 0.989 |
| Hip circumference (cm), mean (SD) | 96.04 (7.06) |  | 96.03 (7.19) | 0.890 |
| Body mass index, mean (SD) | 24.24 (3.75) |  | 24.27 (3.83) | 0.356 |
| Waist-hip ratio, mean (SD) | 0.87 (0.07) |  | 0.87 (0.07) | 0.978 |
| Body fat (%), mean (SD) | 28.53 (7.42) |  | 28.54 (7.42) | 0.950 |
| **Drinking, number (%)** | 4083 (6.1) |  | 838 (5.8) | 0.249 |
| **Smoking, number (%)** | 6450 (9.6) |  | 1377 (9.5) | 0.907 |
| **Regular exercise, number (%)** | 27640 (41.0) |  | 5890 (40.7) | 0.621 |
| **Diet for vegetables, number (%)** | 5.44 (0.87) |  | 5.44 (0.86) | 0.947 |
| **Educational attainment, mean (SD)** | 5.51 (0.96) |  | 5.51 (0.97) | 0.610 |
| **MMSE, mean (SD)** | 27.44 (3.95) |  | 27.39 (2.47) | 0.503 |
| **Personal income/month, mean (SD)** | 5.86 (3.46) |  | 5.85 (3.48) | 0.790 |
| **Household income/month, mean (SD)** | 10.47 (5.16) |  | 10.37 (5.21) | 0.175 |
| **Comorbidities** |  |  |  |  |
| Orthopedic or joint disorders, number (%) | 8121 (12.1) |  | 1703 (11.8) | 0.381 |
| Lung and respiratory diseases, number (%) | 3004 (4.5) |  | 645 (4.5) | 0.986 |
| Cardiovascular diseases, number (%) | 16004 (23.9) |  | 3358 (23.4) | 0.214 |
| Diabetes, number (%) | 3505 (5.2) |  | 744 (5.2) | 0.821 |
| Digestive diseases, number (%) | 16712 (24.9) |  | 3571 (24.8) | 0.830 |
| Mental or emotional disorders, number (%) | 3049 (4.5) |  | 624 (4.3) | 0.298 |
| Nervous system disorders, number (%) | 2171 (3.2) |  | 484 (3.4) | 0.434 |
| Other types of disease, number (%) | 10816 (16.1) |  | 2285 (15.9) | 0.492 |
| Eye diseases , number (%) | 19477 (28.9) |  | 4105 (28.4) | 0.249 |
| Female diseases , number (%) | 14006 (33.8) |  | 3040 (34.2) | 0.385 |
| **Blood pressure and blood test at assessment visit** |  |  |  |  |
| **Cardiovascular related trait** |  |  |  |  |
| Systolic blood pressure (mmHg), mean (SD) | 119.63 (17.93) |  | 119.44 (17.72) | 0.230 |
| Diastolic blood pressure (mmHg), mean (SD) | 73.51 (11.01) |  | 73.36 (11.00) | 0.138 |
| Total cholesterol (mg/dL), mean (SD) | 195.69 (35.84) |  | 195.61 (36.01) | 0.805 |
| Triglyceride (mg/dL), mean (SD) | 116.77 (95.43) |  | 116.35 (96.45) | 0.638 |
| Low-density lipoprotein cholesterol (mg/dL), mean (SD) | 121.07 (31.77) |  | 121.06 (31.74) | 0.960 |
| High-density lipoprotein cholesterol (mg/dL), mean (SD) | 54.34 (13.43) |  | 54.37 (13.52) | 0.794 |
| **Glucose-related trait** |  |  |  |  |
| Fasting glucose (mg/dL), mean (SD) | 95.97 (20.56) |  | 95.99 (20.81) | 0.920 |
| HbA1c (%), mean (SD) | 5.77 (0.80) |  | 5.77 (0.80) | 0.836 |
| N, number; SD, standard deviation. | | | | |
| Age was recorded as a value in years old; educational attainment was recorded as a value ranging from 1 to 7 (1 indicates the lowest level and 7 is the highest level); diet for vegetables was recorded as a value ranging from 1 to 6 (1 indicates the highest level and 7 is the lowest level). | | | | |
| Drinking means current drinking at least 6 months; smoking means current smoking at least 6 months; regular exercise means current regular exercise at least 6 months; diet for vegetables means current diet for vegetables at least for 6 months. | | | | |
