# Supplementary material for: Your height affects your health: genetic determinants and health-related outcomes in Taiwan
Source: BMC Med. 2022 Jul 13;20:250. doi: 10.1186/s12916-022-02450-w (PMC9281111; doi:10.1186/s12916-022-02450-w)
Supplement: Supplementary file 2 — Additional file 2: Table S2. The top lead SNPs in 89 genomic regions that were significantly associated with height (p < 5 × 10^−8^), using LD (r2 < 0.2), in individuals of Han Chinese ancestry. [file 12916_2022_2450_MOESM2_ESM.docx]

| **Additional file 2: Table S2** The top lead SNPs in 89 genomic regions that were significantly associated with height (*p* < 5 × 10^-8^), using LD (r^2^ < 0.2), in individuals of Han Chinese ancestry | | | | | | |
| --- | --- | --- | --- | --- | --- | --- |
| **No.** | **rs ID** | **Gene** | **Chr., position, and allele** | **Novel or Reported lead SNPs** | **Novel or Reported genetic loci** | **Training group (N = 67,452) (*p* < 5 x10^-8^)** |
|  |  |  |  |  |  | [-log10(p)] |
| 1 | rs56265117 | *MFAP2* | 1: 16,980,428 T/C | Novel | Reported | 14.332 |
| 2 | rs61115731 | *FAF1* | 1: 50,932,429 G/C | Novel | Reported | 12.606 |
| 3 | rs3806340 | *PKN2-AS1* | 1: 88,683,110 T/G | Novel | Reported | 12.011 |
| 4 | rs7513580 | *RNA5SP56* | 1: 118,307,286 G/A | Novel | Reported | 17.996 |
| 5 | rs11205303 | *MTMR11* | 1: 149,934,520 T/C | Reported | Reported | 27.245 |
| 6 | rs10489289 | *DNM3* | 1: 172,254,949 T/C | Novel | Reported | 13.209 |
| 7 | rs12047271 | *COLGALT2* | 1: 184,044,357 T/C | Novel | Reported | 12.059 |
| 8 | rs1046017 | *TGFB2* | 1: 218,443,793 G/C | Reported | Reported | 11.56 |
| 9 | rs7538503 | *ZC3H11B* | 1: 219,615,188 A/G | Novel | Reported | 8.948 |
| 10 | rs2367623 | *LTBP1* | 2: 33,202,983 C/A | Novel | Reported | 10.553 |
| 11 | rs3755206 | *CRIM1* | 2: 36,456,285 T/G | Reported | Reported | 16.1 |
| 12 | rs4670703 | *QPCT* | 2: 37,385,957 A/C | Novel | Reported | 12.195 |
| ***13*** | ***rs3791675*** | ***EFEMP1*** | ***2: 55,884,174 T/C*** | ***Reported*** | ***Reported*** | ***36.555*** |
| 14 | rs1824305 | *AC007881.1* | 2: 71,179,325 C/T | Novel | Novel | 22.304 |
| 15 | rs1913671 | *EIF2AK3* | 2: 88,600,365 C/T | Novel | Reported | 11.298 |
| 16 | rs484085 | *USP37* | 2: 218,531,961 T/C | Novel | Reported | 8.924 |
| 17 | rs76709099 | *IHH* | 2: 219,055,182 C/A | Reported | Reported | 13.06 |
| ***18*** | ***rs76803230*** | ***DIS3L2*** | ***2: 232,063,990 T/G*** | ***Novel*** | ***Reported*** | ***41.126*** |
| 19 | rs894857163 | *GIGYF2* | 2: 232,726,985 C/T | Novel | Reported | 9.168 |
| 20 | rs754871503 | *NT5DC2* | 3: 52,525,012 T/C | Novel | Novel | 8.859 |
| 21 | rs13086339 | *RYBP* | 3: 72,428,668 A/C | Novel | Reported | 10.184 |
| 22 | rs4073154 | *H1FX-AS1* | 3: 129,316,642 G/A | Novel | Novel | 11.567 |
| ***23*** | ***rs57345461*** | ***ZBTB38*** | ***3: 141,407,983 A/T*** | ***Novel*** | ***Reported*** | ***41.596*** |
| 24 | rs12639337 | *FNDC3B* | 3: 172,279,149 C/G | Novel | Reported | 13.082 |
| 25 | rs9790124 | *RTP2* | 3: 187,712,899 A/G | Novel | Novel | 11.477 |
| ***26*** | ***rs16895971*** | ***LCORL*** | ***4: 17,883,363 T/C*** | ***Reported*** | ***Reported*** | ***76.433*** |
| 27 | rs7697556 | *ADAMTS3* | 4: 72,649,596 T/C | Reported | Reported | 12.752 |
| 28 | rs1662840 | *PRKG2* | 4: 81,235,255 C/T | Novel | Reported | 14.853 |
| 29 | rs6845999 | *HHIP-AS1* | 4: 144,644,674 C/T | Reported | Reported | 21.137 |
| 30 | rs4273617 | *GHR* | 5: 42,695,369 A/G | Novel | Reported | 12.91 |
| 31 | rs985296 | *MEF2C-AS1* | 5: 89,081,827 G/A | Novel | Reported | 11.055 |
| 32 | rs186405009 | *SLC17A1* | 6: 25,823,049 G/A | Novel | Novel | 9.444 |
| 33 | rs811041 | *HIST1H3E* | 6: 26,225,804 G/C | Novel | Novel | 14.641 |
| ***34*** | ***rs2780226*** | ***HMGA1*** | ***6: 34,231,315 T/C*** | ***Reported*** | ***Reported*** | ***31.757*** |
| 35 | rs1145861 | *AL590824.1* | 6: 80,940,193 G/C | Novel | Novel | 9.939 |
| 36 | rs13197753 | *LIN28B-AS1* | 6: 104,924,810 C/G | Novel | Reported | 11.418 |
| 37 | rs113898003 | *L3MBTL3* | 6: 130,020,090 T/C | Reported | Reported | 15.326 |
| 38 | rs1040525 | *ADGRG6* | 6: 142,382,532 C/T | Novel | Reported | 17.144 |
| 39 | rs73780873 | *ESR1* | 6: 151,829,789 G/A | Novel | Reported | 14.728 |
| 40 | rs1182176 | *GNA12* | 7: 2,834,967 A/G | Novel | Reported | 11.501 |
| 41 | rs185053690 | *KBTBD2* | 7: 32,868,716 G/T | Novel | Reported | 10.207 |
| 42 | rs2960429 | *LOC102723446* | 7: 46,008,459 C/G | Novel | Novel | 11.313 |
| 43 | rs74476179 | *EXTL3* | 8: 28,740,152 G/A | Novel | Reported | 10.019 |
| 44 | rs10957084 | *LOC105375821* | 8: 48,444,248 A/G | Novel | Novel | 10.406 |
| 45 | rs6984782 | *PLAG1* | 8: 56,223,330 T/C | Novel | Reported | 16.616 |
| 46 | rs3886938 | *GSDMC* | 8: 129,725,300 G/T | Novel | Novel | 13.698 |
| 47 | rs1213791479 | *AC105180.2* | 8: 134,354,346 T/C | Novel | Novel | 12.267 |
| 48 | rs1246647183 | *ZFAT* | 8: 134,627,377 T/A | Novel | Reported | 17.209 |
| 49 | rs10120219 | *PTCH1* | 9: 95,602,265 T/C | Reported | Reported | 12.137 |
| 50 | rs10901208 | *FUBP3* | 9: 130,587,253 C/T | Novel | Reported | 11.72 |
| 51 | rs12338076 | *QSOX2* | 9: 136,229,894 A/C | Reported | Reported | 12.44 |
| 52 | rs35859988 | *CCDC3* | 10: 12,902,646 C/T | Reported | Reported | 11.574 |
| 53 | rs1003484 | *IGF2* | 11: 2,146,388 A/G | Novel | Reported | 12.13 |
| 54 | rs78899385 | *PSMA1* | 11: 14,538,479 C/T | Novel | Reported | 15.664 |
| 55 | rs1938679 | *LINC02747* | 11: 69,457,328 C/T | Reported | Reported | 13.525 |
| 56 | rs645935 | *SERPINH1* | 11: 75,568,245 T/C | Reported | Reported | 16.004 |
| 57 | rs57454081 | *PTHLH* | 12: 27,950,661 C/T | Novel | Reported | 10.176 |
| 58 | rs10444558 | *ATP5MC2* | 12: 53,661,701 A/T | Novel | Reported | 12.474 |
| ***59*** | ***rs3816804*** | ***CS*** | ***12: 56,286,961 C/T*** | ***Reported*** | ***Reported*** | ***62.197*** |
| 60 | rs2277339 | *PRIM1* | 12: 56,752,285 T/G | Reported | Reported | 8.742 |
| 61 | rs10878984 | *RPS26P45* | 12: 69,434,754 T/C | Reported | Reported | 16.222 |
| 62 | rs7971647 | *SOCS2* | 12: 93,590,078 T/C | Novel | Reported | 17.959 |
| 63 | rs12424129 | *HELLPAR* | 12: 102,281,461 T/C | Novel | Reported | 27.009 |
| 64 | rs117988169 | *HVCN1* | 12: 110,672,222 G/T | Novel | Novel | 9.528 |
| 65 | rs116873087 | *NAA25* | 12: 112,074,109 G/C | Novel | Novel | 15.532 |
| 66 | rs2093210 | *C14orf39* | 14: 60,490,561 C/T | Reported | Reported | 11.596 |
| 67 | rs7143616 | *ATXN3* | 14: 92,065,114 A/C | Novel | Novel | 23.395 |
| 68 | rs3759556 | *DLK1* | 14: 100,725,962 A/G | Novel | Reported | 9.162 |
| 69 | rs28559787 | *CYP19A1* | 15: 51,304,099 G/T | Novel | Reported | 21.518 |
| 70 | rs2162062 | *VPS13C* | 15: 61,987,738 G/A | Novel | Novel | 12.71 |
| 71 | rs1526080 | *ADAMTSL3* | 15: 83,921,168 A/G | Novel | Reported | 14.343 |
| 72 | rs938608 | *ACAN* | 15: 88,855,374 G/T | Novel | Reported | 10.279 |
| 73 | rs28456063 | *IRAIN* | 15: 98,637,993 C/T | Novel | Reported | 19.684 |
| 74 | rs2573650 | *ADAMTS17* | 15: 99,973,892 A/G | Novel | Reported | 12.859 |
| 75 | rs258324 | *CDK10* | 16: 89,687,847 G/T | Reported | Reported | 14.705 |
| 76 | rs113934718 | *ATAD5* | 17: 30,887,862 C/A | Reported | Reported | 9.56 |
| 77 | rs67474242 | *WNT3* | 17: 46,777,685 G/A | Novel | Novel | 8.865 |
| 78 | rs2411374 | *SNF8* | 17: 48,945,636 T/C | Novel | Novel | 10.827 |
| 79 | rs9905385 | *C17orf82* | 17: 61,420,889 G/A | Novel | Novel | 20.965 |
| 80 | rs2320125 | *CD79B* | 17: 63,930,958 T/C | Novel | Reported | 19.334 |
| 81 | rs4239437 | *CABLES1* | 18: 23,152,260 C/T | Novel | Reported | 28.228 |
| 82 | rs12606199 | *DYM* | 18: 49,045,546 G/A | Novel | Reported | 14.457 |
| 83 | rs3843750 | *SLC44A2* | 19: 10,637,397 G/C | Novel | Reported | 12.81 |
| 84 | rs3213180 | *E2F1* | 20: 33,675,818 G/C | Reported | Reported | 8.737 |
| ***85*** | ***rs143384*** | ***GDF5*** | ***20: 35,437,976 A/G*** | ***Reported*** | ***Reported*** | ***39.442*** |
| 86 | rs4608 | *RPN2* | 20: 37,236,651 C/T | Novel | Reported | 17.652 |
| 87 | rs8121252 | *GNAS* | 20: 58,901,754 C/T | Novel | Reported | 16.639 |
| 88 | rs5754190 | *SYN3* | 22: 32,654,480 T/C | Novel | Reported | 21.365 |
| 89 | rs7290267 | *MIRLET7BHG* | 22: 46,088,855 A/G | Novel | Reported | 10.758 |
| NHGRI, National Human Genome Research Institute (https://www.genome.gov/). | | | | | | |
| Top 7 lead SNPs were highlighted in bold italic with red marks in 89 genomic regions with significant associations (*p* < 5 x 10^-8^) with height using LD (r^2^ < 0.2). | | | | | | |
| Abbreviations: SNP, single nucleotide polymorphism; GWAS, genome-wide association study; No., number; Chr., chromosome; 95% CI, 95% confidence interval; LD, linkage disequilibrium. | | | | | | |
