# Supplementary material for: Your height affects your health: genetic determinants and health-related outcomes in Taiwan
Source: BMC Med. 2022 Jul 13;20:250. doi: 10.1186/s12916-022-02450-w (PMC9281111; doi:10.1186/s12916-022-02450-w)
Supplement: Supplementary file 3 — Additional file 3: Table S3. Replication of a previous GWAS of body height in the SNPs of the training group (313 of 1722 SNPs). [file 12916_2022_2450_MOESM3_ESM.docx]

| **Additional file 3: Table S3** Replication of a previous GWAS of body height in the SNPs of the training group (313 of 1722 SNPs) | | | | | | | | | | | | | | | | | | |
| --- | --- | --- | --- | --- | --- | --- | --- | --- | --- | --- | --- | --- | --- | --- | --- | --- | --- | --- |
| **No.** | **rs ID** | **Gene** | **Chr.** | **Position** | **GWAS catalog (body height trait (EFO_0004339))** | | | | | |  | **Training group (N = 67,452)** | | | | | | |
|  |  |  |  |  | **Mapped trait** | **PubMed id** | **Initial sample size** | **Reported risk allele** | **Reported beta** | **Reported *p*-value** |  | **Minor allele** | **Major allele** | **Risk allele** | **Beta** | **95% CI** | | ***p*-value** |
| 1 | rs2300092 | *MTOR* | 1 | 11206407 | body height | 30595370 | approximately 458,000 European ancestry individuals | ? | NA | ***8.00E-27*** |  | T | C | T | 0.027 | 0.014 | 0.039 | ***2.62E-05*** |
| 2 | rs3738814 | *ATP13A2* | 1 | 17005181 | body height | 25429064 | 36,227 East Asian ancestry individuals | A | 0.061 | ***1.00E-24*** |  | A | G | A | 0.040 | 0.029 | 0.051 | ***1.34E-12*** |
| 3 | rs2275085 | *CCDC17* | 1 | 45620180 | body height | 30595370 | approximately 458,000 European ancestry individuals | ? | NA | ***8.00E-25*** |  | C | T | C | 0.025 | 0.015 | 0.035 | ***9.26E-07*** |
| 4 | rs3014240 | *CCDC17* | 1 | 45623553 | body height | 31562340 | 159,095 Japanese ancestry individuals | ? | 0.023 | ***2.00E-13*** |  | C | G | C | 0.025 | 0.015 | 0.036 | ***9.06E-07*** |
| 5 | rs72904749 | *FAF1* | 1 | 50901748 | body height | 30595370 | approximately 458,000 European ancestry individuals | ? | NA | ***6.00E-60*** |  | T | C | T | 0.060 | 0.043 | 0.076 | ***5.76E-13*** |
| 6 | rs12855 | *CDKN2C* | 1 | 50974421 | body height | 31217584 | 17,286 African American individuals, 22,192 Hispanic/Latino individuals, 4,680 Asian ancestry individuals, 3,939 Native Hawaiian ancestry individuals, 647 Native American ancestry individuals, 1,052 individuals | ? | 0.036 | ***5.00E-06*** |  | T | C | T | 0.060 | 0.043 | 0.076 | ***5.29E-13*** |
| 7 | rs10888713 | *CDKN2C - MIR4421* | 1 | 50997752 | body height | 31562340 | 159,095 Japanese ancestry individuals | ? | 0.051 | ***3.00E-25*** |  | T | A | T | 0.060 | 0.043 | 0.076 | ***7.71E-13*** |
| 8 | rs2666504 | *PATJ* | 1 | 62169409 | body height | 30595370 | approximately 458,000 European ancestry individuals | ? | NA | ***3.00E-10*** |  | C | T | C | 0.024 | 0.013 | 0.034 | ***5.80E-06*** |
| 9 | rs6699417 | *PKN2-AS1* | 1 | 88657760 | body height | 20881960 | 133,653 European ancestry individuals | T | 0.021 | ***5.00E-09*** |  | C | T | T | 0.033 | 0.024 | 0.043 | ***2.07E-11*** |
| 10 | rs10922478 | *PKN2-AS1* | 1 | 88678370 | body height | 30595370 | approximately 458,000 European ancestry individuals | ? | NA | ***2.00E-60*** |  | A | G | G | 0.035 | 0.025 | 0.045 | ***3.57E-12*** |
| 11 | rs9428104 | *RNA5SP56 - PSMC1P12* | 1 | 118312964 | body height | 25282103 | 253,288 European ancestry individuals | A | 0.043 | ***3.00E-36*** |  | A | G | G | 0.045 | 0.035 | 0.055 | ***1.12E-17*** |
| 12 | rs17038164 | *RNA5SP56 - PSMC1P12* | 1 | 118320046 | body height | 30595370 | approximately 458,000 European ancestry individuals | ? | NA | ***5.00E-116*** |  | C | T | T | 0.045 | 0.035 | 0.055 | ***1.21E-17*** |
| 13 | rs7534091 | *RNA5SP56 - PSMC1P12* | 1 | 118321993 | body height | 23563607 | 8,097 European ancestry tall individuals, 8,099 European ancestry short individuals | A | 1.220 | ***2.00E-16*** |  | G | A | A | 0.045 | 0.034 | 0.055 | ***1.39E-17*** |
| 14 | rs12735613 | *RNA5SP56 - PSMC1P12* | 1 | 118341350 | body height | 18391952 | 13,665 European ancestry individuals | A | 0.080 | ***4.00E-11*** |  | A | G | G | 0.039 | 0.028 | 0.050 | ***5.16E-12*** |
| 15 | rs11205303 | *MTMR11* | 1 | 149934520 | body height | 23563607 | 8,097 European ancestry tall individuals, 8,099 European ancestry short individuals | C | 1.250 | ***4.00E-23*** |  | C | T | C | 0.057 | 0.047 | 0.067 | ***5.69E-28*** |
| 16 | rs3767627 | *OTUD7B* | 1 | 149966973 | body height | 25282103 | 253,288 European ancestry individuals | T | 0.036 | ***4.00E-19*** |  | C | T | T | 0.032 | 0.021 | 0.042 | ***5.48E-09*** |
| 17 | rs6587515 | *ENSA - GOLPH3L* | 1 | 150636412 | pericardial adipose tissue measurement, body weight, body height | 27918534 | 3,684 European ancestry women, 3,640 European ancestry men, 1,284 African American women, 877 African American men, 746 Hispanic Ancestry women, 699 Hispanic ancestry men, 390 Asian ancestry women, 378 Asian ancestry men, 294 Old Order Amish (founder/genetic isolate) women, 248 Old Order Amish (founder/genetic isolate) men. | A | 5.940 | ***3.00E-09*** |  | A | G | A | 0.026 | 0.014 | 0.037 | ***2.27E-05*** |
| 18 | rs678962 | *DNM3* | 1 | 172220749 | body height | 18391951 | 30,968 European ancestry individuals | G | 5.400 | ***3.00E-08*** |  | G | T | G | 0.040 | 0.029 | 0.051 | ***3.16E-13*** |
| 19 | rs12410416 | *DNM3* | 1 | 172224680 | body height | 25429064 | 36,227 East Asian ancestry individuals | T | 0.028 | ***2.00E-10*** |  | C | T | C | 0.041 | 0.030 | 0.051 | ***2.64E-13*** |
| 20 | rs2289635 | *DNM3* | 1 | 172239480 | body height | 30595370 | approximately 458,000 European ancestry individuals | ? | NA | ***4.00E-124*** |  | A | G | A | 0.043 | 0.032 | 0.054 | ***9.21E-14*** |
| 21 | rs12409899 | *DNM3* | 1 | 172257871 | body height | 28552196 | 1,249 whole genome sequenced European ancestry individuals, 3,541 whole genome sequenced individuals, 46,910 European ancestry individuals, 471 Carlantino (founder/genetic isolate) individuals, 1,197 Friuli Venezia Giulia (founder/genetic isolate) individuals, 1,043 Mylopotamos (founder/genetic isolate) individuals, 933 Pomak (founder/genetic isolate) individuals, 1,785 Val Borbera (founder/genetic isolate) individuals | C | 0.044 | ***1.00E-07*** |  | G | C | G | 0.042 | 0.031 | 0.054 | ***1.33E-13*** |
| 22 | rs1325596 | *PAPPA2* | 1 | 176824930 | body height | 31217584 | 17,286 African American individuals, 22,192 Hispanic/Latino individuals, 4,680 Asian ancestry individuals, 3,939 Native Hawaiian ancestry individuals, 647 Native American ancestry individuals, 1,052 individuals | ? | 0.029 | ***5.00E-06*** |  | G | A | A | 0.032 | 0.021 | 0.044 | ***6.10E-08*** |
| 23 | rs1044299 | *PAPPA2* | 1 | 176842737 | body height | 30595370 | approximately 458,000 European ancestry individuals | ? | NA | ***2.00E-69*** |  | C | T | T | 0.030 | 0.019 | 0.041 | ***1.14E-07*** |
| 24 | rs10797816 | *LAMC1* | 1 | 183029351 | body height | 30595370 | approximately 458,000 European ancestry individuals | ? | NA | ***5.00E-28*** |  | A | T | A | 0.024 | 0.013 | 0.034 | ***4.73E-06*** |
| 25 | rs10911212 | *LAMC1* | 1 | 183055334 | body height | 31562340 | 159,095 Japanese ancestry individuals | ? | 0.018 | ***1.00E-09*** |  | C | T | C | 0.024 | 0.014 | 0.034 | ***2.83E-06*** |
| 26 | rs756199 | *COLGALT2* | 1 | 184033740 | body height | 20189936 | 19,633 Japanese ancestry individuals | G | 0.050 | ***7.00E-06*** |  | G | A | G | 0.034 | 0.024 | 0.044 | ***1.01E-11*** |
| 27 | rs114661926 | *COLGALT2* | 1 | 184037415 | body height | 28552196 | 1,249 whole genome sequenced European ancestry individuals, 3,541 whole genome sequenced individuals, 46,910 European ancestry individuals, 471 Carlantino (founder/genetic isolate) individuals, 1,197 Friuli Venezia Giulia (founder/genetic isolate) individuals, 1,043 Mylopotamos (founder/genetic isolate) individuals, 933 Pomak (founder/genetic isolate) individuals, 1,785 Val Borbera (founder/genetic isolate) individuals | C | 0.042 | ***1.00E-08*** |  | G | C | G | 0.034 | 0.024 | 0.044 | ***8.20E-12*** |
| 28 | rs3814333 | *COLGALT2 - TSEN15* | 1 | 184037985 | body height | 25282103 | 253,288 European ancestry individuals | T | 0.049 | ***5.00E-51*** |  | T | C | T | 0.035 | 0.025 | 0.045 | ***4.03E-12*** |
| 29 | rs4472734 | *PTPN14* | 1 | 214444842 | body height | 25429064 | 36,227 East Asian ancestry individuals | T | 0.024 | ***1.00E-09*** |  | C | T | C | 0.028 | 0.018 | 0.037 | ***4.96E-08*** |
| 30 | rs12757404 | *PTPN14* | 1 | 214456273 | body height | 30595370 | approximately 458,000 European ancestry individuals | ? | NA | ***1.00E-39*** |  | A | G | A | 0.027 | 0.017 | 0.037 | ***1.69E-07*** |
| 31 | rs10482795 | *TGFB2* | 1 | 218432267 | body height | 30595370 | approximately 458,000 European ancestry individuals | ? | NA | ***3.00E-99*** |  | G | A | A | 0.038 | 0.027 | 0.049 | ***1.10E-11*** |
| 32 | rs6684205 | *TGFB2* | 1 | 218436360 | body height | 20881960 | 133,653 European ancestry individuals | A | 0.028 | ***2.00E-12*** |  | A | G | G | 0.039 | 0.028 | 0.050 | ***3.86E-12*** |
| 33 | rs10165255 | *CYS1* | 2 | 10059474 | body height | 30595370 | approximately 458,000 European ancestry individuals | ? | NA | ***1.00E-45*** |  | A | G | A | 0.030 | 0.016 | 0.044 | ***1.70E-05*** |
| 34 | rs6735681 | *RN7SL104P - AC010145.1* | 2 | 15983051 | body height | 31562340 | 159,095 Japanese ancestry individuals | ? | 0.015 | ***2.00E-07*** |  | T | C | T | 0.023 | 0.013 | 0.033 | ***5.34E-06*** |
| 35 | rs780094 | *GCKR* | 2 | 27518370 | body height | 25282103 | 253,288 European ancestry individuals | T | 0.021 | ***6.00E-12*** |  | T | C | C | 0.022 | 0.012 | 0.031 | ***1.69E-05*** |
| 36 | rs3769528 | *LTBP1* | 2 | 33246125 | body height | 25429064 | 36,227 East Asian ancestry individuals | A | 0.059 | ***7.00E-22*** |  | G | A | A | 0.042 | 0.029 | 0.055 | ***2.85E-10*** |
| 37 | rs3755206 | *CRIM1* | 2 | 36456285 | body height | 25429064 | 36,227 East Asian ancestry individuals | T | 0.048 | ***1.00E-12*** |  | G | T | T | 0.054 | 0.042 | 0.067 | ***7.94E-17*** |
| 38 | rs6544743 | *RNU6-566P - PDSS1P2* | 2 | 44163230 | body height | 30595370 | approximately 458,000 European ancestry individuals | ? | NA | ***4.00E-43*** |  | T | G | T | 0.024 | 0.013 | 0.035 | ***1.52E-05*** |
| 39 | rs58754091 | *PNPT1 - EFEMP1* | 2 | 55842539 | body height | 28552196 | 1,249 whole genome sequenced European ancestry individuals, 3,541 whole genome sequenced individuals, 46,910 European ancestry individuals, 471 Carlantino (founder/genetic isolate) individuals, 1,197 Friuli Venezia Giulia (founder/genetic isolate) individuals, 1,043 Mylopotamos (founder/genetic isolate) individuals, 933 Pomak (founder/genetic isolate) individuals, 1,785 Val Borbera (founder/genetic isolate) individuals | A | 0.049 | ***9.00E-09*** |  | G | A | G | 0.071 | 0.053 | 0.088 | ***3.80E-15*** |
| 40 | rs1367226 | *PNPT1 - EFEMP1* | 2 | 55862405 | body height | 20881960 | 133,653 European ancestry individuals | ? | NA | ***4.00E-08*** |  | A | G | A | 0.046 | 0.029 | 0.063 | ***7.55E-08*** |
| 41 | rs3791675 | *EFEMP1* | 2 | 55884174 | body height | 18391952 | 13,665 European ancestry individuals | C | 0.090 | ***2.00E-12*** |  | C | T | C | 0.075 | 0.064 | 0.087 | ***2.78E-37*** |
| 42 | rs1432559 | *AC011306.1* | 2 | 55962483 | body height | 30595370 | approximately 458,000 European ancestry individuals | ? | NA | ***9.00E-65*** |  | G | T | G | 0.055 | 0.030 | 0.080 | ***1.32E-05*** |
| 43 | rs6724998 | *MIR217HG, AC011306.1* | 2 | 55969288 | body height | 28552196 | 1,249 whole genome sequenced European ancestry individuals, 3,541 whole genome sequenced individuals, 46,910 European ancestry individuals, 471 Carlantino (founder/genetic isolate) individuals, 1,197 Friuli Venezia Giulia (founder/genetic isolate) individuals, 1,043 Mylopotamos (founder/genetic isolate) individuals, 933 Pomak (founder/genetic isolate) individuals, 1,785 Val Borbera (founder/genetic isolate) individuals | T | 0.035 | ***6.00E-08*** |  | C | T | C | 0.037 | 0.025 | 0.048 | ***7.28E-10*** |
| 44 | rs4241349 | *ANTXR1* | 2 | 69103152 | body height | 31562340 | 159,095 Japanese ancestry individuals | ? | 0.020 | ***3.00E-10*** |  | G | A | G | 0.026 | 0.014 | 0.037 | ***9.55E-06*** |
| 45 | rs12612930 | *ZNF638* | 2 | 71298655 | body height | 23456168 | 6,534 Han Chinese ancestry individuals | C | 0.100 | ***2.00E-10*** |  | T | C | C | 0.044 | 0.034 | 0.054 | ***3.39E-18*** |
| 46 | rs4852777 | *ZNF638* | 2 | 71307031 | body height | 30595370 | approximately 458,000 European ancestry individuals | ? | NA | ***1.00E-86*** |  | C | G | G | 0.044 | 0.034 | 0.055 | ***3.76E-16*** |
| 47 | rs11681299 | *EIF2AK3* | 2 | 88602214 | body height | 30595370 | approximately 458,000 European ancestry individuals | ? | NA | ***2.00E-68*** |  | T | C | T | 0.030 | 0.020 | 0.039 | ***3.16E-09*** |
| 48 | rs11684404 | *EIF2AK3* | 2 | 88625104 | body height | 25282103 | 253,288 European ancestry individuals | T | 0.032 | ***9.00E-25*** |  | T | C | C | 0.034 | 0.024 | 0.044 | ***7.95E-12*** |
| 49 | rs994532 | *DIRC3* | 2 | 217419487 | body height | 31562340 | 159,095 Japanese ancestry individuals | ? | 0.025 | ***2.00E-16*** |  | A | G | A | 0.033 | 0.022 | 0.044 | ***1.22E-08*** |
| 50 | rs994533 | *DIRC3* | 2 | 217419555 | body height | 25282103 | 253,288 European ancestry individuals | C | 0.027 | ***1.00E-17*** |  | G | C | G | 0.033 | 0.022 | 0.044 | ***1.25E-08*** |
| 51 | rs642950 | *USP37* | 2 | 218469913 | body height | 28552196 | 1,249 whole genome sequenced European ancestry individuals, 3,541 whole genome sequenced individuals, 46,910 European ancestry individuals, 471 Carlantino (founder/genetic isolate) individuals, 1,197 Friuli Venezia Giulia (founder/genetic isolate) individuals, 1,043 Mylopotamos (founder/genetic isolate) individuals, 933 Pomak (founder/genetic isolate) individuals, 1,785 Val Borbera (founder/genetic isolate) individuals | T | 0.036 | ***2.00E-07*** |  | C | T | T | 0.035 | 0.024 | 0.047 | ***2.56E-09*** |
| 52 | rs503188 | *CNOT9 - PLCD4* | 2 | 218599081 | body height | 30595370 | approximately 458,000 European ancestry individuals | ? | NA | ***5.00E-73*** |  | G | A | A | 0.036 | 0.024 | 0.047 | ***1.25E-09*** |
| 53 | rs611203 | *CNOT9 - PLCD4* | 2 | 218607602 | body height | 25429064 | 36,227 East Asian ancestry individuals | A | 0.037 | ***2.00E-10*** |  | G | A | A | 0.035 | 0.024 | 0.047 | ***2.03E-09*** |
| 54 | rs76709099 | *IHH* | 2 | 219055182 | body height | 31562340 | 159,095 Japanese ancestry individuals | ? | 0.045 | ***2.00E-14*** |  | A | C | C | 0.064 | 0.047 | 0.080 | ***8.72E-14*** |
| 55 | rs16859517 | *NHEJ1, AC068946.1* | 2 | 219084462 | body height | 25282103 | 253,288 European ancestry individuals | T | 0.067 | ***5.00E-17*** |  | T | C | T | 0.034 | 0.024 | 0.044 | ***1.36E-11*** |
| 56 | rs3103267 | *DIS3L2* | 2 | 232123872 | body height | 23563607 | 8,097 European ancestry tall individuals, 8,099 European ancestry short individuals | C | 1.160 | ***3.00E-08*** |  | C | A | C | 0.055 | 0.043 | 0.067 | ***2.02E-20*** |
| 57 | rs3116168 | *DIS3L2* | 2 | 232125121 | body height | 30595370 | approximately 458,000 European ancestry individuals | ? | NA | ***7.00E-104*** |  | C | T | C | 0.055 | 0.043 | 0.066 | ***3.49E-20*** |
| 58 | rs7571816 | *DIS3L2* | 2 | 232212354 | body height | 20189936 | 19,633 Japanese ancestry individuals | A | 0.060 | ***9.00E-09*** |  | G | A | A | 0.064 | 0.054 | 0.074 | ***7.58E-38*** |
| 59 | rs2343240 | *DIS3L2* | 2 | 232222773 | body height | 31217584 | 17,286 African American individuals, 22,192 Hispanic/Latino individuals, 4,680 Asian ancestry individuals, 3,939 Native Hawaiian ancestry individuals, 647 Native American ancestry individuals, 1,052 individuals | ? | 0.049 | ***2.00E-07*** |  | C | T | T | 0.058 | 0.048 | 0.068 | ***2.72E-29*** |
| 60 | rs6717918 | *DIS3L2* | 2 | 232290400 | body height | 19570815 | 10,074 European ancestry individuals | T | 0.440 | ***3.00E-09*** |  | C | T | T | 0.048 | 0.038 | 0.058 | ***7.19E-22*** |
| 61 | rs2564923 | *AC096887.1* | 3 | 53069246 | body height | 31562340 | 159,095 Japanese ancestry individuals | ? | 0.029 | ***1.00E-16*** |  | A | G | A | 0.027 | 0.016 | 0.038 | ***1.49E-06*** |
| 62 | rs2564921 | *AC096887.1, RFT1* | 3 | 53091569 | body height | 23563607 | 8,097 European ancestry tall individuals, 8,099 European ancestry short individuals | T | 1.150 | ***2.00E-12*** |  | T | C | T | 0.025 | 0.014 | 0.036 | ***8.11E-06*** |
| 63 | rs9863706 | *RYBP, RYBP* | 3 | 72388262 | body height | 20881960 | 133,653 European ancestry individuals | T | 0.031 | ***4.00E-13*** |  | T | C | C | 0.035 | 0.024 | 0.046 | ***3.06E-10*** |
| 64 | rs7428883 | *COPG1* | 3 | 129265010 | body height | 31562340 | 159,095 Japanese ancestry individuals | ? | 0.032 | ***7.00E-23*** |  | A | G | G | 0.038 | 0.027 | 0.048 | ***6.79E-12*** |
| 65 | rs7636293 | *H1FX-AS1 - AC137695.1* | 3 | 129327063 | body height | 25429064 | 36,227 East Asian ancestry individuals | T | 0.029 | ***4.00E-11*** |  | C | T | T | 0.035 | 0.025 | 0.045 | ***3.67E-11*** |
| 66 | rs9812461 | *RYK* | 3 | 134101845 | body height | 31562340 | 159,095 Japanese ancestry individuals | ? | 0.023 | ***5.00E-16*** |  | G | A | A | 0.026 | 0.016 | 0.036 | ***2.51E-07*** |
| 67 | rs9841212 | *HMGB3P13 - ANAPC13* | 3 | 134473096 | body height | 31562340 | 159,095 Japanese ancestry individuals | ? | 0.028 | ***9.00E-19*** |  | C | T | T | 0.024 | 0.013 | 0.036 | ***2.79E-05*** |
| 68 | rs6440003 | *ZBTB38* | 3 | 141375367 | body height | 18391952 | 13,665 European ancestry individuals | A | 0.070 | ***2.00E-24*** |  | A | G | A | 0.070 | 0.060 | 0.081 | ***7.45E-40*** |
| 69 | rs6763931 | *ZBTB38* | 3 | 141383991 | body height | 25429064 | 36,227 East Asian ancestry individuals | A | 0.055 | ***6.00E-34*** |  | A | G | A | 0.070 | 0.060 | 0.081 | ***4.83E-40*** |
| 70 | rs74888405 | *ZBTB38* | 3 | 141399390 | body height | 31562340 | 159,095 Japanese ancestry individuals | ? | 0.068 | ***4.00E-106*** |  | C | T | C | 0.073 | 0.062 | 0.084 | ***3.04E-39*** |
| 71 | rs1991431 | *ZBTB38* | 3 | 141414608 | body height | 23563607 | 8,097 European ancestry tall individuals, 8,099 European ancestry short individuals | A | 1.330 | ***4.00E-47*** |  | A | G | A | 0.052 | 0.040 | 0.064 | ***5.03E-18*** |
| 72 | rs9825379 | *ZBTB38* | 3 | 141418193 | body height | 20189936 | 19,633 Japanese ancestry individuals | A | 0.070 | ***6.00E-09*** |  | A | G | A | 0.068 | 0.056 | 0.081 | ***7.97E-26*** |
| 73 | rs10513137 | *ZBTB38* | 3 | 141424588 | body height | 19396169 | 8,842 Korean ancestry individuals | A | 0.460 | ***6.00E-12*** |  | A | G | A | 0.064 | 0.053 | 0.076 | ***1.09E-27*** |
| 74 | rs1055153 | *WWTR1, WWTR1-AS1* | 3 | 149657086 | body height | 30595370 | approximately 458,000 European ancestry individuals | ? | NA | ***4.00E-10*** |  | T | G | G | 0.046 | 0.030 | 0.062 | ***1.33E-08*** |
| 75 | rs116959924 | *WWTR1, WWTR1-AS1* | 3 | 149657805 | body height | 31562340 | 159,095 Japanese ancestry individuals | ? | 0.034 | ***1.00E-12*** |  | T | G | G | 0.045 | 0.030 | 0.061 | ***2.35E-08*** |
| 76 | rs2686544 | *AC079943.2* | 3 | 158066427 | body height | 31562340 | 159,095 Japanese ancestry individuals | ? | 0.024 | ***7.00E-17*** |  | T | C | C | 0.022 | 0.012 | 0.032 | ***1.37E-05*** |
| 77 | rs1730040 | *RSRC1* | 3 | 158313173 | body height | 30595370 | approximately 458,000 European ancestry individuals | ? | NA | ***1.00E-39*** |  | A | G | G | 0.028 | 0.018 | 0.039 | ***8.17E-08*** |
| 78 | rs7652177 | *FNDC3B* | 3 | 172251287 | body height | 25282103 | 253,288 European ancestry individuals | C | 0.038 | ***3.00E-39*** |  | C | G | G | 0.037 | 0.027 | 0.047 | ***1.84E-13*** |
| 79 | rs62281815 | *FNDC3B* | 3 | 172252520 | body height | 28552196 | 1,249 whole genome sequenced European ancestry individuals, 3,541 whole genome sequenced individuals, 46,910 European ancestry individuals, 471 Carlantino (founder/genetic isolate) individuals, 1,197 Friuli Venezia Giulia (founder/genetic isolate) individuals, 1,043 Mylopotamos (founder/genetic isolate) individuals, 933 Pomak (founder/genetic isolate) individuals, 1,785 Val Borbera (founder/genetic isolate) individuals | A | 0.050 | ***1.00E-13*** |  | T | A | A | 0.036 | 0.026 | 0.046 | ***7.81E-13*** |
| 80 | rs6774762 | *GHSR* | 3 | 172447200 | body height | 31562340 | 159,095 Japanese ancestry individuals | ? | 0.034 | ***2.00E-24*** |  | G | A | A | 0.037 | 0.024 | 0.050 | ***1.80E-08*** |
| 81 | rs61732778 | *AC072022.1, BCL6* | 3 | 187725526 | body height | 30595370 | approximately 458,000 European ancestry individuals | ? | NA | ***4.00E-55*** |  | A | G | A | 0.047 | 0.031 | 0.062 | ***5.82E-09*** |
| 82 | rs1056932 | *AC072022.1, BCL6* | 3 | 187729244 | body height | 31562340 | 159,095 Japanese ancestry individuals | ? | 0.032 | ***4.00E-16*** |  | G | A | G | 0.038 | 0.026 | 0.050 | ***8.87E-10*** |
| 83 | rs16895707 | *FAM184B* | 4 | 17779586 | body height | 28552196 | 1,249 whole genome sequenced European ancestry individuals, 3,541 whole genome sequenced individuals, 46,910 European ancestry individuals, 471 Carlantino (founder/genetic isolate) individuals, 1,197 Friuli Venezia Giulia (founder/genetic isolate) individuals, 1,043 Mylopotamos (founder/genetic isolate) individuals, 933 Pomak (founder/genetic isolate) individuals, 1,785 Val Borbera (founder/genetic isolate) individuals | T | 0.041 | ***9.00E-07*** |  | T | C | C | 0.052 | 0.041 | 0.062 | ***3.79E-22*** |
| 84 | rs13131350 | *LCORL* | 4 | 17875864 | body height | 25429064 | 36,227 East Asian ancestry individuals | A | 0.095 | ***1.00E-53*** |  | G | A | A | 0.101 | 0.090 | 0.112 | ***1.50E-75*** |
| 85 | rs16896068 | *LCORL* | 4 | 17943217 | body height | 18391952 | 13,665 European ancestry individuals | A | 0.070 | ***2.00E-13*** |  | A | G | G | 0.065 | 0.051 | 0.079 | ***5.65E-20*** |
| 86 | rs6824748 | *LCORL* | 4 | 17995443 | body height | 30595370 | approximately 458,000 European ancestry individuals | ? | NA | ***2.00E-268*** |  | A | G | G | 0.065 | 0.051 | 0.079 | ***3.98E-20*** |
| 87 | rs2061456 | *LCORL* | 4 | 17996803 | body height | 28552196 | 1,249 whole genome sequenced European ancestry individuals, 3,541 whole genome sequenced individuals, 46,910 European ancestry individuals, 471 Carlantino (founder/genetic isolate) individuals, 1,197 Friuli Venezia Giulia (founder/genetic isolate) individuals, 1,043 Mylopotamos (founder/genetic isolate) individuals, 933 Pomak (founder/genetic isolate) individuals, 1,785 Val Borbera (founder/genetic isolate) individuals | A | 0.066 | ***1.00E-19*** |  | A | C | A | 0.080 | 0.070 | 0.090 | ***9.15E-57*** |
| 88 | rs6853156 | *REST* | 4 | 56908677 | body height | 30595370 | approximately 458,000 European ancestry individuals | ? | NA | ***2.00E-62*** |  | T | C | T | 0.029 | 0.019 | 0.039 | ***2.28E-08*** |
| 89 | rs2227901 | *REST* | 4 | 56932023 | body height | 25429064 | 36,227 East Asian ancestry individuals | A | 0.025 | ***3.00E-09*** |  | A | G | A | 0.028 | 0.018 | 0.038 | ***4.36E-08*** |
| 90 | rs3733309 | *POLR2B* | 4 | 56991022 | body height | 25429064 | 36,227 East Asian ancestry individuals | A | 0.021 | ***2.00E-08*** |  | G | A | G | 0.024 | 0.014 | 0.034 | ***2.50E-06*** |
| 91 | rs4694504 | *ADAMTS3 - HNRNPA1P67* | 4 | 72630974 | body height | 28552196 | 1,249 whole genome sequenced European ancestry individuals, 3,541 whole genome sequenced individuals, 46,910 European ancestry individuals, 471 Carlantino (founder/genetic isolate) individuals, 1,197 Friuli Venezia Giulia (founder/genetic isolate) individuals, 1,043 Mylopotamos (founder/genetic isolate) individuals, 933 Pomak (founder/genetic isolate) individuals, 1,785 Val Borbera (founder/genetic isolate) individuals | A | 0.040 | ***3.00E-10*** |  | A | G | G | 0.036 | 0.027 | 0.046 | ***4.85E-13*** |
| 92 | rs7697556 | *ADAMTS3 - HNRNPA1P67* | 4 | 72649596 | body height | 20881960 | 133,653 European ancestry individuals | T | 0.028 | ***2.00E-14*** |  | C | T | T | 0.037 | 0.027 | 0.047 | ***1.77E-13*** |
| 93 | rs16848425 | *ADAMTS3 - HNRNPA1P67* | 4 | 72650108 | body height | 25429064 | 36,227 East Asian ancestry individuals | T | 0.034 | ***7.00E-12*** |  | T | C | T | 0.039 | 0.027 | 0.051 | ***5.88E-10*** |
| 94 | rs55854248 | *ADAMTS3 - HNRNPA1P67* | 4 | 72664035 | body height | 31562340 | 159,095 Japanese ancestry individuals | ? | 0.039 | ***3.00E-32*** |  | T | C | T | 0.037 | 0.025 | 0.050 | ***2.27E-09*** |
| 95 | rs710841 | *PRKG2 - RNU5A-2P* | 4 | 81228677 | body height | 18391951 | 30,968 European ancestry individuals | A | 5.000 | ***2.00E-06*** |  | T | C | T | 0.052 | 0.039 | 0.064 | ***3.95E-15*** |
| 96 | rs788867 | *PRKG2 - RNU5A-2P* | 4 | 81228852 | body height | 20881960 | 133,653 European ancestry individuals | T | 0.043 | ***9.00E-28*** |  | G | T | G | 0.051 | 0.039 | 0.064 | ***4.49E-15*** |
| 97 | rs17556750 | *PRKG2 - RNU5A-2P* | 4 | 81234414 | body height | 25282103 | 253,288 European ancestry individuals | A | 0.046 | ***8.00E-48*** |  | A | C | A | 0.052 | 0.039 | 0.065 | ***2.31E-15*** |
| 98 | rs1975474 | *PRKG2 - RNU5A-2P* | 4 | 81257783 | body height | 28552196 | 1,249 whole genome sequenced European ancestry individuals, 3,541 whole genome sequenced individuals, 46,910 European ancestry individuals, 471 Carlantino (founder/genetic isolate) individuals, 1,197 Friuli Venezia Giulia (founder/genetic isolate) individuals, 1,043 Mylopotamos (founder/genetic isolate) individuals, 933 Pomak (founder/genetic isolate) individuals, 1,785 Val Borbera (founder/genetic isolate) individuals | T | 0.060 | ***6.00E-18*** |  | G | T | G | 0.052 | 0.039 | 0.064 | ***2.97E-15*** |
| 99 | rs1878528 | *PRKG2 - RNU5A-2P* | 4 | 81263080 | body height | 23563607 | 8,097 European ancestry tall individuals, 8,099 European ancestry short individuals | G | 1.200 | ***4.00E-18*** |  | G | A | G | 0.050 | 0.038 | 0.063 | ***7.42E-15*** |
| 100 | rs4463061 | *PRKG2 - RNU5A-2P* | 4 | 81278988 | body height | 31562340 | 159,095 Japanese ancestry individuals | ? | 0.045 | ***3.00E-38*** |  | C | T | C | 0.050 | 0.037 | 0.063 | ***1.52E-14*** |
| 101 | rs17017911 | *AC107223.1, GUSBP5* | 4 | 143559481 | body height | 30595370 | approximately 458,000 European ancestry individuals | ? | NA | ***6.00E-34*** |  | G | A | A | 0.022 | 0.012 | 0.032 | ***2.19E-05*** |
| 102 | rs62346126 | *AC098588.3, AC098588.2* | 4 | 144639014 | body height | 30595370 | approximately 458,000 European ancestry individuals | ? | NA | ***1.00E-300*** |  | A | C | A | 0.042 | 0.032 | 0.052 | ***1.51E-16*** |
| 103 | rs6845999 | *AC098588.2, HHIP-AS1, AC098588.3* | 4 | 144644674 | body height | 25429064 | 36,227 East Asian ancestry individuals | T | 0.037 | ***4.00E-14*** |  | T | C | T | 0.058 | 0.046 | 0.070 | ***7.29E-22*** |
| 104 | rs13125694 | *AC098588.2, AC098588.3, HHIP-AS1* | 4 | 144645696 | body height | 31562340 | 159,095 Japanese ancestry individuals | ? | 0.045 | ***1.00E-29*** |  | T | C | T | 0.057 | 0.045 | 0.069 | ***4.76E-21*** |
| 105 | rs7689420 | *AC098588.2, AC098588.3, HHIP-AS1, HHIP* | 4 | 144647200 | body height | 23563607 | 8,097 European ancestry tall individuals, 8,099 European ancestry short individuals | C | 1.330 | ***4.00E-21*** |  | T | C | C | 0.037 | 0.027 | 0.046 | ***3.73E-13*** |
| 106 | rs1812175 | *HHIP-AS1, AC098588.3, HHIP, AC098588.2* | 4 | 144653692 | body height | 18391951 | 30,968 European ancestry individuals | C | 8.300 | ***1.00E-11*** |  | A | G | G | 0.036 | 0.026 | 0.046 | ***4.91E-13*** |
| 107 | rs4240326 | *ANAPC10, AC098588.2* | 4 | 144918112 | body height | 25282103 | 253,288 European ancestry individuals | A | 0.040 | ***3.00E-43*** |  | A | G | A | 0.028 | 0.017 | 0.039 | ***7.17E-07*** |
| 108 | rs301901 | *NIPBL* | 5 | 37046524 | body height | 25282103 | 253,288 European ancestry individuals | A | 0.024 | ***4.00E-16*** |  | A | G | A | 0.024 | 0.014 | 0.034 | ***2.04E-06*** |
| 109 | rs6180 | *GHR* | 5 | 42719137 | body height | 25429064 | 36,227 East Asian ancestry individuals | A | 0.018 | ***8.00E-06*** |  | A | C | A | 0.024 | 0.014 | 0.034 | ***2.45E-06*** |
| 110 | rs2972770 | *AC113368.1 - CCDC152* | 5 | 42731670 | body height | 31562340 | 159,095 Japanese ancestry individuals | ? | 0.029 | ***2.00E-24*** |  | G | A | G | 0.027 | 0.017 | 0.037 | ***1.26E-07*** |
| 111 | rs4865956 | *AK4P2 - SLC38A9* | 5 | 55586677 | body height | 30595370 | approximately 458,000 European ancestry individuals | ? | NA | ***9.00E-87*** |  | T | A | T | 0.024 | 0.014 | 0.035 | ***5.07E-06*** |
| 112 | rs6453383 | *SCAMP1* | 5 | 78406536 | body height | 30595370 | approximately 458,000 European ancestry individuals | ? | NA | ***2.00E-08*** |  | G | A | G | 0.032 | 0.021 | 0.043 | ***1.03E-08*** |
| 113 | rs6894139 | *MEF2C-AS1* | 5 | 89031965 | body height | 25282103 | 253,288 European ancestry individuals | T | 0.030 | ***6.00E-24*** |  | T | G | T | 0.031 | 0.021 | 0.041 | ***7.48E-10*** |
| 114 | rs2217257 | *MEF2C-AS1* | 5 | 89046775 | body height | 31562340 | 159,095 Japanese ancestry individuals | ? | 0.034 | ***2.00E-32*** |  | T | C | C | 0.033 | 0.024 | 0.043 | ***2.30E-11*** |
| 115 | rs9327294 | *CEP120* | 5 | 123381125 | body height | 31562340 | 159,095 Japanese ancestry individuals | ? | 0.022 | ***9.00E-15*** |  | C | A | C | 0.022 | 0.013 | 0.032 | ***9.04E-06*** |
| 116 | rs7706662 | *CEP120* | 5 | 123419868 | body height | 30595370 | approximately 458,000 European ancestry individuals | ? | NA | ***4.00E-45*** |  | T | C | C | 0.023 | 0.013 | 0.032 | ***7.16E-06*** |
| 117 | rs2908532 | *AC091825.3 - SPRY4* | 5 | 142242319 | body height | 31562340 | 159,095 Japanese ancestry individuals | ? | 0.018 | ***2.00E-07*** |  | A | C | A | 0.027 | 0.016 | 0.037 | ***1.23E-06*** |
| 118 | rs145613835 | *ARHGAP26* | 5 | 143084873 | body height | 31562340 | 159,095 Japanese ancestry individuals | ? | 0.034 | ***3.00E-09*** |  | T | C | C | 0.053 | 0.035 | 0.071 | ***1.13E-08*** |
| 119 | rs2974438 | *SLIT3* | 5 | 168823898 | body height | 25282103 | 253,288 European ancestry individuals | A | 0.037 | ***4.00E-24*** |  | A | G | G | 0.030 | 0.017 | 0.043 | ***3.53E-06*** |
| 120 | rs4282339 | *SLIT3* | 5 | 168829235 | body height | 20881960 | 133,653 European ancestry individuals | A | 0.036 | ***7.00E-16*** |  | A | G | G | 0.029 | 0.017 | 0.042 | ***5.28E-06*** |
| 121 | rs12153391 | *AC011410.1 - SMIM23* | 5 | 171776434 | body height | 20881960 | 133,653 European ancestry individuals | A | 0.030 | ***4.00E-12*** |  | A | C | C | 0.029 | 0.019 | 0.039 | ***1.22E-08*** |
| 122 | rs4868126 | *SMIM23 - FBXW11* | 5 | 171856465 | body height | 25282103 | 253,288 European ancestry individuals | T | 0.036 | ***3.00E-29*** |  | T | G | G | 0.031 | 0.020 | 0.041 | ***4.13E-09*** |
| 123 | rs722585 | *GMDS* | 6 | 1775629 | body height | 31562340 | 159,095 Japanese ancestry individuals | ? | 0.020 | ***6.00E-12*** |  | A | G | G | 0.023 | 0.013 | 0.034 | ***1.61E-05*** |
| 124 | rs806794 | *H2BC7* | 6 | 26200449 | body height | 25282103 | 253,288 European ancestry individuals | A | 0.060 | ***5.00E-74*** |  | A | G | A | 0.046 | 0.034 | 0.057 | ***8.34E-15*** |
| 125 | rs10946808 | *H2AC9P* | 6 | 26233159 | body height | 18391951 | 30,968 European ancestry individuals | A | 5.600 | ***6.00E-10*** |  | A | G | A | 0.047 | 0.035 | 0.058 | ***2.71E-15*** |
| 126 | rs766406 | *H4C8 - H3C9P* | 6 | 26319360 | body height | 28552196 | 1,249 whole genome sequenced European ancestry individuals, 3,541 whole genome sequenced individuals, 46,910 European ancestry individuals, 471 Carlantino (founder/genetic isolate) individuals, 1,197 Friuli Venezia Giulia (founder/genetic isolate) individuals, 1,043 Mylopotamos (founder/genetic isolate) individuals, 933 Pomak (founder/genetic isolate) individuals, 1,785 Val Borbera (founder/genetic isolate) individuals | T | 0.050 | ***2.00E-12*** |  | G | T | G | 0.057 | 0.040 | 0.074 | ***1.95E-11*** |
| 127 | rs78566116 | *TSBP1-AS1 - HLA-DRA* | 6 | 32428369 | body height | 29782485 | 253,288 European ancestry individuals | T | 0.012 | ***4.00E-12*** |  | T | G | G | 0.040 | 0.022 | 0.057 | ***5.70E-06*** |
| 128 | rs12214804 | *CYCSP55 - HMGA1* | 6 | 34221089 | body height | 25282103 | 253,288 European ancestry individuals | T | 0.084 | ***2.00E-49*** |  | C | T | C | 0.094 | 0.078 | 0.110 | ***4.15E-31*** |
| 129 | rs1759645 | *CYCSP55 - HMGA1* | 6 | 34227089 | body height | 23563607 | 8,097 European ancestry tall individuals, 8,099 European ancestry short individuals | C | 1.290 | ***5.00E-15*** |  | C | T | C | 0.090 | 0.074 | 0.105 | ***4.44E-30*** |
| 130 | rs2780226 | *CYCSP55 - HMGA1* | 6 | 34231315 | body weights and measures, body height | 30166351 | 4,988 Spanish ancestry individuals | C | 0.067 | ***7.00E-109*** |  | C | T | C | 0.095 | 0.079 | 0.110 | ***1.75E-32*** |
| 131 | rs57026767 | *AL354740.1* | 6 | 34251921 | body height | 28270201 | 19,965 British ancestry individuals from 6863 families. | T | 0.006 | ***5.00E-11*** |  | C | T | C | 0.091 | 0.076 | 0.107 | ***1.20E-30*** |
| 132 | rs6918981 | *AL354740.1* | 6 | 34270737 | body height | 19893584 | 8,842 Korean ancestry individuals | G | 0.550 | ***2.00E-08*** |  | G | A | G | 0.086 | 0.071 | 0.101 | ***1.65E-28*** |
| 133 | rs12209223 | *FILIP1* | 6 | 75454873 | body height | 25282103 | 253,288 European ancestry individuals | A | 0.051 | ***5.00E-25*** |  | A | C | A | 0.050 | 0.033 | 0.067 | ***1.02E-08*** |
| 134 | rs648831 | *BCKDHB* | 6 | 80246491 | body height | 25282103 | 253,288 European ancestry individuals | T | 0.031 | ***3.00E-26*** |  | C | T | T | 0.024 | 0.014 | 0.034 | ***1.53E-06*** |
| 135 | rs3805859 | *BCKDHB* | 6 | 80339229 | body height | 31562340 | 159,095 Japanese ancestry individuals | ? | 0.027 | ***1.00E-21*** |  | C | A | C | 0.025 | 0.015 | 0.035 | ***5.62E-07*** |
| 136 | rs9391253 | *LIN28B-AS1* | 6 | 104919741 | body height | 23563607 | 8,097 European ancestry tall individuals, 8,099 European ancestry short individuals | T | 1.160 | ***5.00E-12*** |  | T | A | T | 0.036 | 0.025 | 0.046 | ***1.60E-11*** |
| 137 | rs12207399 | *LIN28B, LIN28B-AS1* | 6 | 104937737 | body height | 31562340 | 159,095 Japanese ancestry individuals | ? | 0.038 | ***1.00E-33*** |  | G | A | G | 0.036 | 0.026 | 0.047 | ***7.09E-12*** |
| 138 | rs395962 | *LIN28B* | 6 | 104949543 | body height | 30595370 | approximately 458,000 European ancestry individuals | ? | NA | ***1.00E-175*** |  | T | G | T | 0.036 | 0.026 | 0.047 | ***7.14E-12*** |
| 139 | rs314276 | *LIN28B* | 6 | 104960124 | body height | 28552196 | 1,249 whole genome sequenced European ancestry individuals, 3,541 whole genome sequenced individuals, 46,910 European ancestry individuals, 471 Carlantino (founder/genetic isolate) individuals, 1,197 Friuli Venezia Giulia (founder/genetic isolate) individuals, 1,043 Mylopotamos (founder/genetic isolate) individuals, 933 Pomak (founder/genetic isolate) individuals, 1,785 Val Borbera (founder/genetic isolate) individuals | A | 0.055 | ***2.00E-14*** |  | A | C | A | 0.035 | 0.025 | 0.046 | ***3.26E-11*** |
| 140 | rs113898003 | *L3MBTL3* | 6 | 130020090 | body height | 28552196 | 1,249 whole genome sequenced European ancestry individuals, 3,541 whole genome sequenced individuals, 46,910 European ancestry individuals, 471 Carlantino (founder/genetic isolate) individuals, 1,197 Friuli Venezia Giulia (founder/genetic isolate) individuals, 1,043 Mylopotamos (founder/genetic isolate) individuals, 933 Pomak (founder/genetic isolate) individuals, 1,785 Val Borbera (founder/genetic isolate) individuals | T | 0.046 | ***9.00E-09*** |  | C | T | T | 0.042 | 0.032 | 0.052 | ***4.72E-16*** |
| 141 | rs1415701 | *L3MBTL3* | 6 | 130024690 | body height | 23563607 | 8,097 European ancestry tall individuals, 8,099 European ancestry short individuals | G | 1.190 | ***5.00E-13*** |  | A | G | G | 0.041 | 0.031 | 0.051 | ***1.19E-15*** |
| 142 | rs7740107 | *L3MBTL3* | 6 | 130053316 | body height | 25282103 | 253,288 European ancestry individuals | A | 0.042 | ***3.00E-36*** |  | T | A | T | 0.058 | 0.031 | 0.085 | ***2.29E-05*** |
| 143 | rs9321266 | *EPB41L2* | 6 | 130995318 | body height | 30595370 | approximately 458,000 European ancestry individuals | ? | NA | ***1.00E-62*** |  | A | G | G | 0.032 | 0.019 | 0.045 | ***1.82E-06*** |
| 144 | rs7765757 | *EPB41L2* | 6 | 131050608 | body height | 31562340 | 159,095 Japanese ancestry individuals | ? | 0.036 | ***2.00E-17*** |  | C | T | T | 0.038 | 0.022 | 0.053 | ***1.29E-06*** |
| 145 | rs7741741 | *ADGRG6* | 6 | 142334664 | body height | 23563607 | 8,097 European ancestry tall individuals, 8,099 European ancestry short individuals | T | 1.240 | ***1.00E-20*** |  | A | T | T | 0.043 | 0.033 | 0.053 | ***3.17E-17*** |
| 146 | rs6570507 | *ADGRG6* | 6 | 142358435 | body height | 20397748 | 11,536 European ancestry individuals | G | 0.080 | ***2.00E-07*** |  | A | G | G | 0.043 | 0.033 | 0.053 | ***2.39E-17*** |
| 147 | rs9496369 | *ADGRG6* | 6 | 142403781 | body height | 30595370 | approximately 458,000 European ancestry individuals | ? | NA | ***3.00E-217*** |  | T | C | C | 0.044 | 0.034 | 0.054 | ***1.67E-17*** |
| 148 | rs7753012 | *ADGRG6* | 6 | 142424746 | body height | 28270201 | 19,965 British ancestry individuals from 6863 families. | G | 0.005 | ***8.00E-14*** |  | T | G | T | 0.030 | 0.018 | 0.041 | ***9.00E-07*** |
| 149 | rs263179 | *AL359313.1* | 6 | 142542751 | body height | 28270201 | 19,965 British ancestry individuals from 6863 families. | C | 0.005 | ***8.00E-12*** |  | C | T | T | 0.035 | 0.025 | 0.045 | ***1.39E-11*** |
| 150 | rs2071454 | *ESR1* | 6 | 151805689 | body height | 31562340 | 159,095 Japanese ancestry individuals | ? | 0.043 | ***9.00E-46*** |  | G | T | G | 0.044 | 0.033 | 0.055 | ***4.36E-15*** |
| 151 | rs2982712 | *ESR1* | 6 | 152037044 | body height | 23563607 | 8,097 European ancestry tall individuals, 8,099 European ancestry short individuals | C | 1.170 | ***4.00E-10*** |  | T | C | C | 0.027 | 0.015 | 0.039 | ***1.31E-05*** |
| 152 | rs3020359 | *ESR1* | 6 | 152044128 | body height | 31562340 | 159,095 Japanese ancestry individuals | ? | 0.018 | ***1.00E-10*** |  | T | C | C | 0.023 | 0.013 | 0.033 | ***1.11E-05*** |
| 153 | rs73029259 | *AL078602.1* | 6 | 163690316 | body height | 29782485 | 253,288 European ancestry individuals | A | 0.014 | ***1.00E-15*** |  | A | T | A | 0.037 | 0.020 | 0.054 | ***1.99E-05*** |
| 154 | rs798557 | *AMZ1* | 7 | 2719348 | body height | 31562340 | 159,095 Japanese ancestry individuals | ? | 0.051 | ***3.00E-55*** |  | A | G | G | 0.041 | 0.028 | 0.053 | ***1.02E-10*** |
| 155 | rs798554 | *AMZ1* | 7 | 2720161 | body height | 23563607 | 8,097 European ancestry tall individuals, 8,099 European ancestry short individuals | C | 1.240 | ***4.00E-23*** |  | T | C | C | 0.041 | 0.028 | 0.053 | ***9.91E-11*** |
| 156 | rs798544 | *AMZ1* | 7 | 2723468 | body height | 18391951 | 30,968 European ancestry individuals | G | 5.900 | ***7.00E-15*** |  | T | C | C | 0.040 | 0.028 | 0.052 | ***2.22E-10*** |
| 157 | rs798491 | *GNA12, AMZ1* | 7 | 2760887 | body height | 30595370 | approximately 458,000 European ancestry individuals | ? | NA | ***2.00E-241*** |  | G | A | A | 0.040 | 0.028 | 0.052 | ***8.92E-11*** |
| 158 | rs7777484 | *GNA12, AMZ1* | 7 | 2774637 | body height | 25429064 | 36,227 East Asian ancestry individuals | A | 0.059 | ***4.00E-27*** |  | G | A | A | 0.040 | 0.028 | 0.052 | ***1.09E-10*** |
| 159 | rs1182188 | *GNA12* | 7 | 2830351 | body height | 31217584 | 17,286 African American individuals, 22,192 Hispanic/Latino individuals, 4,680 Asian ancestry individuals, 3,939 Native Hawaiian ancestry individuals, 647 Native American ancestry individuals, 1,052 individuals | ? | 0.044 | ***2.00E-11*** |  | C | T | T | 0.045 | 0.032 | 0.058 | ***8.65E-12*** |
| 160 | rs57246313 | *AC018706.1 - AC010719.1* | 7 | 25850077 | body height | 30595370 | approximately 458,000 European ancestry individuals | ? | NA | ***5.00E-34*** |  | A | G | A | 0.024 | 0.014 | 0.034 | ***3.01E-06*** |
| 161 | rs1007358 | *ZNF619P1 - AC023669.1* | 7 | 46161757 | body height | 25282103 | 253,288 European ancestry individuals | A | 0.021 | ***9.00E-10*** |  | G | A | G | 0.036 | 0.023 | 0.049 | ***1.12E-07*** |
| 162 | rs42377 | *CDK6* | 7 | 92614358 | body height | 28552196 | 1,249 whole genome sequenced European ancestry individuals, 3,541 whole genome sequenced individuals, 46,910 European ancestry individuals, 471 Carlantino (founder/genetic isolate) individuals, 1,197 Friuli Venezia Giulia (founder/genetic isolate) individuals, 1,043 Mylopotamos (founder/genetic isolate) individuals, 933 Pomak (founder/genetic isolate) individuals, 1,785 Val Borbera (founder/genetic isolate) individuals | A | 0.059 | ***4.00E-18*** |  | A | G | A | 0.035 | 0.019 | 0.052 | ***2.44E-05*** |
| 163 | rs445 | *CDK6* | 7 | 92779056 | body height | 31562340 | 159,095 Japanese ancestry individuals | ? | 0.035 | ***6.00E-30*** |  | T | C | C | 0.025 | 0.015 | 0.035 | ***1.86E-06*** |
| 164 | rs76364830 | *DLC1* | 8 | 13514611 | body height | 30595370 | approximately 458,000 European ancestry individuals | ? | NA | ***1.00E-43*** |  | A | G | G | 0.041 | 0.023 | 0.059 | ***9.41E-06*** |
| 165 | rs3929448 | *AC041040.1 - AC026904.1* | 8 | 48463007 | body height | 31562340 | 159,095 Japanese ancestry individuals | ? | 0.021 | ***6.00E-11*** |  | C | T | C | 0.032 | 0.021 | 0.043 | ***7.26E-09*** |
| 166 | rs10958476 | *PLAG1* | 8 | 56183249 | body height | 18391951 | 30,968 European ancestry individuals | C | 5.400 | ***7.00E-08*** |  | C | T | C | 0.030 | 0.017 | 0.042 | ***2.60E-06*** |
| 167 | rs13273123 | *PLAG1* | 8 | 56188232 | body height | 25429064 | 36,227 East Asian ancestry individuals | A | 0.099 | ***3.00E-29*** |  | G | A | A | 0.078 | 0.059 | 0.097 | ***3.43E-16*** |
| 168 | rs72656010 | *PLAG1* | 8 | 56209656 | body height | 30595370 | approximately 458,000 European ancestry individuals | ? | NA | ***4.00E-172*** |  | C | T | T | 0.079 | 0.060 | 0.098 | ***2.87E-16*** |
| 169 | rs36112366 | *AC107952.2 - AC107952.1* | 8 | 56226117 | body height | 31562340 | 159,095 Japanese ancestry individuals | ? | 0.078 | ***1.00E-53*** |  | G | T | T | 0.075 | 0.057 | 0.093 | ***5.41E-16*** |
| 170 | rs67742458 | *AC107952.1 - SDR16C5* | 8 | 56258088 | body height | 31562340 | 159,095 Japanese ancestry individuals | ? | 0.068 | ***2.00E-13*** |  | G | A | A | 0.072 | 0.052 | 0.092 | ***1.01E-12*** |
| 171 | rs7460090 | *AC107952.1 - SDR16C5* | 8 | 56281604 | body height | 20881960 | 133,653 European ancestry individuals | T | 0.058 | ***8.00E-27*** |  | C | T | T | 0.072 | 0.053 | 0.092 | ***9.19E-13*** |
| 172 | rs7815909 | *AC107952.1 - SDR16C5* | 8 | 56287803 | body height | 25429064 | 36,227 East Asian ancestry individuals | A | 0.102 | ***5.00E-25*** |  | G | A | A | 0.070 | 0.050 | 0.089 | ***5.84E-12*** |
| 173 | rs4075154 | *AC107952.1 - SDR16C5* | 8 | 56296687 | body height | 23563607 | 8,097 European ancestry tall individuals, 8,099 European ancestry short individuals | A | 1.290 | ***3.00E-09*** |  | G | A | A | 0.070 | 0.049 | 0.090 | ***6.87E-11*** |
| 174 | rs7842996 | *HIGD1AP18 - AC062004.1* | 8 | 77194904 | body height | 28552196 | 1,249 whole genome sequenced European ancestry individuals, 3,541 whole genome sequenced individuals, 46,910 European ancestry individuals, 471 Carlantino (founder/genetic isolate) individuals, 1,197 Friuli Venezia Giulia (founder/genetic isolate) individuals, 1,043 Mylopotamos (founder/genetic isolate) individuals, 933 Pomak (founder/genetic isolate) individuals, 1,785 Val Borbera (founder/genetic isolate) individuals | A | 0.051 | ***3.00E-13*** |  | A | T | A | 0.031 | 0.019 | 0.043 | ***6.50E-07*** |
| 175 | rs4735677 | *HIGD1AP18 - AC062004.1* | 8 | 77235955 | body height | 25282103 | 253,288 European ancestry individuals | A | 0.037 | ***6.00E-30*** |  | T | A | T | 0.030 | 0.018 | 0.042 | ***1.65E-06*** |
| 176 | rs7846385 | *HIGD1AP18 - AC062004.1* | 8 | 77247943 | body height | 18391951 | 30,968 European ancestry individuals | C | 5.000 | ***5.00E-08*** |  | C | T | C | 0.030 | 0.018 | 0.042 | ***1.61E-06*** |
| 177 | rs7817087 | *AC105177.1 - EIF3H* | 8 | 116552698 | body height | 31562340 | 159,095 Japanese ancestry individuals | ? | 0.019 | ***1.00E-10*** |  | A | G | G | 0.026 | 0.016 | 0.035 | ***4.15E-07*** |
| 178 | rs4876361 | *AC105177.1 - EIF3H* | 8 | 116554149 | body height | 30595370 | approximately 458,000 European ancestry individuals | ? | NA | ***1.00E-35*** |  | A | G | G | 0.025 | 0.016 | 0.035 | ***4.71E-07*** |
| 179 | rs6992491 | *PVT1* | 8 | 128185657 | body height | 31562340 | 159,095 Japanese ancestry individuals | ? | 0.022 | ***7.00E-14*** |  | G | C | G | 0.022 | 0.012 | 0.032 | ***1.68E-05*** |
| 180 | rs7815955 | *AC103718.1* | 8 | 129707321 | body height | 30595370 | approximately 458,000 European ancestry individuals | ? | NA | ***1.00E-132*** |  | A | T | A | 0.042 | 0.031 | 0.053 | ***1.31E-13*** |
| 181 | rs6470763 | *AC103718.1* | 8 | 129708400 | body height | 31562340 | 159,095 Japanese ancestry individuals | ? | 0.031 | ***7.00E-26*** |  | G | C | G | 0.042 | 0.031 | 0.053 | ***1.37E-13*** |
| 182 | rs4733724 | *AC103718.1* | 8 | 129711482 | body height | 25282103 | 253,288 European ancestry individuals | A | 0.050 | ***1.00E-41*** |  | A | G | A | 0.042 | 0.031 | 0.053 | ***8.75E-14*** |
| 183 | rs6470764 | *AC103718.1* | 8 | 129713419 | body height | 20881960 | 133,653 European ancestry individuals | T | 0.050 | ***2.00E-28*** |  | C | T | C | 0.042 | 0.031 | 0.052 | ***1.30E-13*** |
| 184 | rs28701981 | *PTCH1* | 9 | 95455299 | body height | 30595370 | approximately 458,000 European ancestry individuals | ? | NA | ***2.00E-140*** |  | C | T | C | 0.033 | 0.022 | 0.043 | ***4.29E-10*** |
| 185 | rs10512248 | *PTCH1* | 9 | 95497421 | body height | 18391952 | 13,665 European ancestry individuals | G | 0.050 | ***4.00E-11*** |  | G | T | G | 0.037 | 0.026 | 0.047 | ***3.65E-12*** |
| 186 | rs28778940 | *PTCH1 - AL392185.1* | 9 | 95536070 | body height | 31562340 | 159,095 Japanese ancestry individuals | ? | 0.039 | ***2.00E-27*** |  | A | G | A | 0.042 | 0.031 | 0.054 | ***2.81E-12*** |
| 187 | rs10120219 | *AL392185.1* | 9 | 95602265 | body height | 31562340 | 159,095 Japanese ancestry individuals | ? | 0.032 | ***9.00E-29*** |  | C | T | T | 0.036 | 0.026 | 0.046 | ***7.29E-13*** |
| 188 | rs62558978 | *AL354861.2 - LINC00476* | 9 | 95752752 | body height | 31562340 | 159,095 Japanese ancestry individuals | ? | 0.022 | ***5.00E-10*** |  | A | G | G | 0.032 | 0.020 | 0.044 | ***1.01E-07*** |
| 189 | rs34575265 | *LINC01505* | 9 | 106181520 | body height | 30595370 | approximately 458,000 European ancestry individuals | ? | NA | ***9.00E-50*** |  | T | C | C | 0.022 | 0.012 | 0.032 | ***2.66E-05*** |
| 190 | rs7858562 | *ZNF483, PTGR1* | 9 | 111562668 | body height | 31562340 | 159,095 Japanese ancestry individuals | ? | 0.018 | ***4.00E-07*** |  | G | A | A | 0.025 | 0.014 | 0.037 | ***2.33E-05*** |
| 191 | rs12344818 | *AL691420.1* | 9 | 115728289 | body height | 30595370 | approximately 458,000 European ancestry individuals | ? | NA | ***5.00E-33*** |  | T | C | C | 0.031 | 0.019 | 0.043 | ***1.73E-07*** |
| 192 | rs3789280 | *PAPPA* | 9 | 116191093 | body height | 30595370 | approximately 458,000 European ancestry individuals | ? | NA | ***1.00E-14*** |  | A | T | A | 0.036 | 0.020 | 0.053 | ***1.23E-05*** |
| 193 | rs803733 | *RABGAP1* | 9 | 123070600 | body height | 30595370 | approximately 458,000 European ancestry individuals | ? | NA | ***2.00E-17*** |  | C | T | T | 0.027 | 0.017 | 0.038 | ***5.72E-07*** |
| 194 | rs7466269 | *FUBP3* | 9 | 130588697 | body height | 25282103 | 253,288 European ancestry individuals | A | 0.033 | ***1.00E-27*** |  | A | G | A | 0.035 | 0.025 | 0.045 | ***3.71E-12*** |
| 195 | rs7861829 | *QSOX2* | 9 | 136228197 | body height | 28552196 | 1,249 whole genome sequenced European ancestry individuals, 3,541 whole genome sequenced individuals, 46,910 European ancestry individuals, 471 Carlantino (founder/genetic isolate) individuals, 1,197 Friuli Venezia Giulia (founder/genetic isolate) individuals, 1,043 Mylopotamos (founder/genetic isolate) individuals, 933 Pomak (founder/genetic isolate) individuals, 1,785 Val Borbera (founder/genetic isolate) individuals | C | 0.046 | ***4.00E-10*** |  | G | C | G | 0.039 | 0.027 | 0.051 | ***8.02E-11*** |
| 196 | rs12338076 | *QSOX2* | 9 | 136229894 | body height | 20189936 | 19,633 Japanese ancestry individuals | C | 0.060 | ***2.00E-08*** |  | C | A | C | 0.040 | 0.029 | 0.050 | ***3.63E-13*** |
| 197 | rs35092545 | *AL353586.1, CCDC3* | 10 | 12901108 | body height | 28552196 | 1,249 whole genome sequenced European ancestry individuals, 3,541 whole genome sequenced individuals, 46,910 European ancestry individuals, 471 Carlantino (founder/genetic isolate) individuals, 1,197 Friuli Venezia Giulia (founder/genetic isolate) individuals, 1,043 Mylopotamos (founder/genetic isolate) individuals, 933 Pomak (founder/genetic isolate) individuals, 1,785 Val Borbera (founder/genetic isolate) individuals | T | 0.038 | ***5.00E-07*** |  | T | G | G | 0.039 | 0.027 | 0.051 | ***1.79E-10*** |
| 198 | rs35954730 | *AL353586.1, CCDC3* | 10 | 12901111 | body height | 30595370 | approximately 458,000 European ancestry individuals | ? | NA | ***9.00E-71*** |  | A | G | G | 0.039 | 0.027 | 0.051 | ***1.79E-10*** |
| 199 | rs35741360 | *CCDC3, AL353586.1* | 10 | 12903180 | body height | 31562340 | 159,095 Japanese ancestry individuals | ? | 0.042 | ***3.00E-32*** |  | A | G | G | 0.046 | 0.033 | 0.059 | ***3.21E-12*** |
| 200 | rs779933 | *ZMIZ1* | 10 | 79158760 | body height | 25429064 | 36,227 East Asian ancestry individuals | A | 0.032 | ***8.00E-08*** |  | A | G | G | 0.028 | 0.017 | 0.039 | ***8.37E-07*** |
| 201 | rs1815314 | *ZMIZ1* | 10 | 79169036 | body height | 25282103 | 253,288 European ancestry individuals | A | 0.022 | ***5.00E-14*** |  | A | G | G | 0.032 | 0.018 | 0.046 | ***1.13E-05*** |
| 202 | rs780151 | *ZMIZ1* | 10 | 79171724 | body height | 23563607 | 8,097 European ancestry tall individuals, 8,099 European ancestry short individuals | G | 1.130 | ***2.00E-09*** |  | A | G | G | 0.031 | 0.017 | 0.046 | ***1.44E-05*** |
| 203 | rs7092536 | *PPIF - ZCCHC24* | 10 | 79371576 | body height | 31562340 | 159,095 Japanese ancestry individuals | ? | 0.026 | ***4.00E-18*** |  | A | C | A | 0.030 | 0.019 | 0.040 | ***1.82E-08*** |
| 204 | rs2450444 | *PCGF5* | 10 | 91250626 | body height | 32376654 | 453,169 European ancestry individuals | G | 0.008 | ***2.00E-08*** |  | A | G | A | 0.043 | 0.023 | 0.063 | ***2.48E-05*** |
| 205 | rs2648725 | *PCGF5* | 10 | 91255322 | body height | 30595370 | approximately 458,000 European ancestry individuals | ? | NA | ***5.00E-49*** |  | A | T | A | 0.044 | 0.024 | 0.064 | ***1.61E-05*** |
| 206 | rs35506085 | *AC132217.2, INS-IGF2, IGF2-AS* | 11 | 2144346 | body height | 30595370 | approximately 458,000 European ancestry individuals | ? | NA | ***8.00E-58*** |  | G | A | G | 0.036 | 0.026 | 0.046 | ***1.05E-12*** |
| 207 | rs79732015 | *COPB1* | 11 | 14449593 | body height | 31562340 | 159,095 Japanese ancestry individuals | ? | 0.077 | ***1.00E-47*** |  | G | T | T | 0.079 | 0.060 | 0.098 | ***8.17E-16*** |
| 208 | rs17790804 | *PSMC3* | 11 | 47425651 | body height | 31562340 | 159,095 Japanese ancestry individuals | ? | 0.033 | ***7.00E-30*** |  | C | T | C | 0.031 | 0.021 | 0.042 | ***8.88E-09*** |
| 209 | rs1938679 | *AP000439.1 - AP000439.3* | 11 | 69457328 | body height | 25429064 | 36,227 East Asian ancestry individuals | T | 0.032 | ***1.00E-11*** |  | T | C | C | 0.038 | 0.029 | 0.048 | ***2.98E-14*** |
| 210 | rs645935 | *SERPINH1* | 11 | 75568245 | body height | 31562340 | 159,095 Japanese ancestry individuals | ? | 0.032 | ***1.00E-29*** |  | C | T | T | 0.042 | 0.032 | 0.051 | ***9.90E-17*** |
| 211 | rs659418 | *SERPINH1 - AP001922.5* | 11 | 75573289 | body height | 28552196 | 1,249 whole genome sequenced European ancestry individuals, 3,541 whole genome sequenced individuals, 46,910 European ancestry individuals, 471 Carlantino (founder/genetic isolate) individuals, 1,197 Friuli Venezia Giulia (founder/genetic isolate) individuals, 1,043 Mylopotamos (founder/genetic isolate) individuals, 933 Pomak (founder/genetic isolate) individuals, 1,785 Val Borbera (founder/genetic isolate) individuals | T | 0.059 | ***3.00E-10*** |  | G | T | G | 0.041 | 0.031 | 0.051 | ***4.67E-16*** |
| 212 | rs12230367 | *AC008011.1 - PTHLH* | 12 | 27955755 | body height | 31562340 | 159,095 Japanese ancestry individuals | ? | 0.047 | ***7.00E-22*** |  | A | G | A | 0.047 | 0.033 | 0.062 | ***1.84E-10*** |
| 213 | rs10467109 | *ATF7-NPFF, AC023509.6, ATF7* | 12 | 53577542 | body height | 30595370 | approximately 458,000 European ancestry individuals | ? | NA | ***7.00E-17*** |  | C | T | C | 0.028 | 0.018 | 0.038 | ***4.37E-08*** |
| 214 | rs7970462 | *ATF7 - ATP5MC2* | 12 | 53628818 | body height | 31562340 | 159,095 Japanese ancestry individuals | ? | 0.029 | ***5.00E-22*** |  | C | T | C | 0.033 | 0.023 | 0.044 | ***1.19E-10*** |
| 215 | rs11170631 | *ATP5MC2* | 12 | 53647408 | body height | 20189936 | 19,633 Japanese ancestry individuals | C | 0.050 | ***9.00E-07*** |  | C | T | C | 0.034 | 0.024 | 0.044 | ***4.42E-11*** |
| 216 | rs59917308 | *ANKRD52 - COQ10A* | 12 | 56264924 | body height | 30595370 | approximately 458,000 European ancestry individuals | ? | NA | ***3.00E-32*** |  | T | C | T | 0.069 | 0.039 | 0.099 | ***5.37E-06*** |
| 217 | rs3816804 | *AC073896.1, CS* | 12 | 56286961 | body height | 23456168 | 6,534 Han Chinese ancestry individuals | C | 0.123 | ***3.00E-09*** |  | T | C | C | 0.112 | 0.099 | 0.126 | ***6.35E-63*** |
| 218 | rs7133285 | *AC073896.2, AC073896.1* | 12 | 56305645 | body height | 31562340 | 159,095 Japanese ancestry individuals | ? | 0.105 | ***1.00E-189*** |  | A | G | G | 0.111 | 0.097 | 0.124 | ***1.64E-60*** |
| 219 | rs3809128 | *AC073896.2* | 12 | 56316135 | body height | 25429064 | 36,227 East Asian ancestry individuals | T | 0.083 | ***7.00E-35*** |  | T | C | C | 0.112 | 0.098 | 0.125 | ***6.03E-62*** |
| 220 | rs2066808 | *STAT2* | 12 | 56344189 | body height | 25429064 | 36,227 East Asian ancestry individuals | A | 0.067 | ***9.00E-12*** |  | G | A | G | 0.068 | 0.038 | 0.098 | ***7.52E-06*** |
| 221 | rs2066807 | *STAT2* | 12 | 56346898 | body height | 20881960 | 133,653 European ancestry individuals | C | 0.054 | ***1.00E-13*** |  | G | C | G | 0.068 | 0.038 | 0.098 | ***8.63E-06*** |
| 222 | rs2277339 | *HSD17B6, PRIM1* | 12 | 56752285 | body height | 30595370 | approximately 458,000 European ancestry individuals | ? | NA | ***3.00E-24*** |  | G | T | T | 0.037 | 0.025 | 0.049 | ***1.81E-09*** |
| 223 | rs10747784 | *CTDSP2 - AC083805.1* | 12 | 57857579 | body height | 30595370 | approximately 458,000 European ancestry individuals | ? | NA | ***8.00E-38*** |  | G | A | G | 0.027 | 0.015 | 0.038 | ***4.71E-06*** |
| 224 | rs17179670 | *HMGA2* | 12 | 65956032 | body height | 31562340 | 159,095 Japanese ancestry individuals | ? | 0.080 | ***1.00E-33*** |  | G | A | A | 0.066 | 0.043 | 0.090 | ***3.27E-08*** |
| 225 | rs10748128 | *RPS26P45 - LINC02373* | 12 | 69433878 | body height | 25282103 | 253,288 European ancestry individuals | T | 0.038 | ***4.00E-29*** |  | G | T | T | 0.042 | 0.032 | 0.052 | ***8.47E-16*** |
| 226 | rs10878984 | *RPS26P45 - LINC02373* | 12 | 69434754 | body height | 28552196 | 1,249 whole genome sequenced European ancestry individuals, 3,541 whole genome sequenced individuals, 46,910 European ancestry individuals, 471 Carlantino (founder/genetic isolate) individuals, 1,197 Friuli Venezia Giulia (founder/genetic isolate) individuals, 1,043 Mylopotamos (founder/genetic isolate) individuals, 933 Pomak (founder/genetic isolate) individuals, 1,785 Val Borbera (founder/genetic isolate) individuals | T | 0.048 | ***4.00E-13*** |  | C | T | T | 0.044 | 0.034 | 0.054 | ***5.99E-17*** |
| 227 | rs11107116 | *SOCS2 - AC012085.1* | 12 | 93584728 | body height | 20881960 | 133,653 European ancestry individuals | T | 0.052 | ***1.00E-34*** |  | T | G | T | 0.043 | 0.032 | 0.053 | ***9.78E-16*** |
| 228 | rs3847787 | *CRADD* | 12 | 93813756 | body height | 30595370 | approximately 458,000 European ancestry individuals | ? | NA | ***7.00E-26*** |  | G | A | G | 0.022 | 0.012 | 0.033 | ***1.91E-05*** |
| 229 | rs2271266 | *NUP37* | 12 | 102112266 | body height | 25429064 | 36,227 East Asian ancestry individuals | T | 0.077 | ***4.00E-46*** |  | C | T | T | 0.058 | 0.047 | 0.069 | ***3.65E-26*** |
| 230 | rs2292303 | *NUP37* | 12 | 102119753 | body height | 19893584 | 8,842 Korean ancestry individuals | C | 0.510 | ***8.00E-06*** |  | C | G | G | 0.058 | 0.047 | 0.069 | ***1.64E-26*** |
| 231 | rs113701417 | *PARPBP* | 12 | 102157937 | body height | 31562340 | 159,095 Japanese ancestry individuals | ? | 0.064 | ***3.00E-97*** |  | A | G | G | 0.059 | 0.048 | 0.069 | ***4.95E-27*** |
| 232 | rs76157805 | *PARPBP* | 12 | 102178155 | body height | 30595370 | approximately 458,000 European ancestry individuals | ? | NA | ***2.00E-42*** |  | G | A | A | 0.059 | 0.048 | 0.070 | ***3.43E-27*** |
| 233 | rs7313075 | *HELLPAR* | 12 | 102236901 | body height | 25429064 | 36,227 East Asian ancestry individuals | A | 0.074 | ***4.00E-47*** |  | A | C | C | 0.059 | 0.048 | 0.069 | ***9.77E-27*** |
| 234 | rs1520223 | *LINC02456, HELLPAR* | 12 | 102332560 | body height | 19893584 | 8,842 Korean ancestry individuals | C | 0.560 | ***9.00E-07*** |  | C | T | T | 0.054 | 0.043 | 0.064 | ***2.41E-22*** |
| 235 | rs5742692 | *LINC02456, IGF1* | 12 | 102405820 | body height | 20189936 | 19,633 Japanese ancestry individuals | G | 0.070 | ***4.00E-08*** |  | G | A | A | 0.048 | 0.037 | 0.059 | ***2.16E-18*** |
| 236 | rs671 | *ALDH2* | 12 | 111803962 | body height | 31562340 | 159,095 Japanese ancestry individuals | ? | 0.030 | ***2.00E-14*** |  | A | G | G | 0.045 | 0.034 | 0.056 | ***6.09E-16*** |
| 237 | rs1254276 | *LINC02322 - C14orf39* | 14 | 60380283 | body height | 28552196 | 1,249 whole genome sequenced European ancestry individuals, 3,541 whole genome sequenced individuals, 46,910 European ancestry individuals, 471 Carlantino (founder/genetic isolate) individuals, 1,197 Friuli Venezia Giulia (founder/genetic isolate) individuals, 1,043 Mylopotamos (founder/genetic isolate) individuals, 933 Pomak (founder/genetic isolate) individuals, 1,785 Val Borbera (founder/genetic isolate) individuals | T | 0.049 | ***4.00E-14*** |  | C | T | T | 0.042 | 0.030 | 0.054 | ***1.31E-11*** |
| 238 | rs2093210 | *C14orf39* | 14 | 60490561 | body height | 23563607 | 8,097 European ancestry tall individuals, 8,099 European ancestry short individuals | C | 1.160 | ***8.00E-13*** |  | T | C | C | 0.043 | 0.031 | 0.055 | ***2.54E-12*** |
| 239 | rs175426 | *TMED10* | 14 | 75157431 | body height | 30595370 | approximately 458,000 European ancestry individuals | ? | NA | ***9.00E-10*** |  | T | C | T | 0.029 | 0.016 | 0.042 | ***2.44E-05*** |
| 240 | rs910316 | *TMED10* | 14 | 75159339 | body height | 19343178 | 12,611 European ancestry individuals | ? | 0.050 | ***1.00E-07*** |  | A | C | A | 0.029 | 0.016 | 0.042 | ***1.20E-05*** |
| 241 | rs10467747 | *TC2N, TC2N* | 14 | 91858114 | body height | 30595370 | approximately 458,000 European ancestry individuals | ? | NA | ***2.00E-31*** |  | C | T | C | 0.028 | 0.017 | 0.039 | ***9.10E-07*** |
| 242 | rs7154721 | *FBLN5 - TRIP11* | 14 | 91961004 | body height | 25282103 | 253,288 European ancestry individuals | T | 0.027 | ***5.00E-20*** |  | C | T | T | 0.049 | 0.038 | 0.060 | ***9.57E-18*** |
| 243 | rs10129429 | *FBLN5 - TRIP11* | 14 | 91964487 | body height | 30595370 | approximately 458,000 European ancestry individuals | ? | NA | ***4.00E-80*** |  | A | G | G | 0.048 | 0.037 | 0.060 | ***2.66E-17*** |
| 244 | rs1133441 | *TRIP11* | 14 | 91968721 | body height | 31562340 | 159,095 Japanese ancestry individuals | ? | 0.049 | ***5.00E-64*** |  | A | T | T | 0.051 | 0.041 | 0.062 | ***1.34E-21*** |
| 245 | rs8007661 | *TRIP11* | 14 | 91993614 | body height | 18391950 | 15,821 European ancestry individuals | T | 0.420 | ***6.00E-10*** |  | T | C | C | 0.045 | 0.035 | 0.055 | ***1.30E-18*** |
| 246 | rs7158300 | *TRIP11* | 14 | 92016604 | body height | 25429064 | 36,227 East Asian ancestry individuals | T | 0.056 | ***4.00E-27*** |  | T | C | C | 0.051 | 0.040 | 0.061 | ***1.48E-21*** |
| 247 | rs7156335 | *ITPK1, ITPK1* | 14 | 92939887 | body height | 30595370 | approximately 458,000 European ancestry individuals | ? | NA | ***2.00E-28*** |  | C | T | C | 0.049 | 0.028 | 0.070 | ***4.46E-06*** |
| 248 | rs4900575 | *MARK3* | 14 | 103432832 | body height | 30595370 | approximately 458,000 European ancestry individuals | ? | NA | ***2.00E-56*** |  | C | G | G | 0.027 | 0.017 | 0.037 | ***2.13E-07*** |
| 249 | rs12592845 | *DUT - FBN1* | 15 | 48392761 | body height | 30595370 | approximately 458,000 European ancestry individuals | ? | NA | ***3.00E-38*** |  | T | C | C | 0.028 | 0.015 | 0.041 | ***1.99E-05*** |
| 250 | rs12909519 | *MIR4713HG* | 15 | 51049670 | body height | 31562340 | 159,095 Japanese ancestry individuals | ? | 0.026 | ***8.00E-19*** |  | A | C | C | 0.028 | 0.018 | 0.038 | ***2.85E-08*** |
| 251 | rs28757157 | *MIR4713HG, CYP19A1* | 15 | 51253204 | body height | 30595370 | approximately 458,000 European ancestry individuals | ? | NA | ***5.00E-42*** |  | T | C | C | 0.044 | 0.032 | 0.055 | ***1.96E-13*** |
| 252 | rs2305707 | *CYP19A1, MIR4713HG* | 15 | 51277213 | body height | 20189936 | 19,633 Japanese ancestry individuals | G | 0.060 | ***7.00E-07*** |  | G | A | A | 0.050 | 0.039 | 0.061 | ***6.53E-20*** |
| 253 | rs10519302 | *CYP19A1* | 15 | 51307486 | body height | 25429064 | 36,227 East Asian ancestry individuals | A | 0.069 | ***3.00E-31*** |  | G | A | A | 0.053 | 0.042 | 0.064 | ***7.21E-22*** |
| 254 | rs731820 | *NPM1P47 - C2CD4B* | 15 | 62085937 | body height | 31562340 | 159,095 Japanese ancestry individuals | ? | 0.022 | ***2.00E-13*** |  | A | G | G | 0.036 | 0.026 | 0.046 | ***1.64E-12*** |
| 255 | rs7178424 | *NPM1P47 - C2CD4B* | 15 | 62088060 | body height | 20881960 | 133,653 European ancestry individuals | T | 0.021 | ***6.00E-09*** |  | T | C | C | 0.037 | 0.027 | 0.047 | ***5.89E-13*** |
| 256 | rs975210 | *TLE3* | 15 | 70072013 | body height | 25282103 | 253,288 European ancestry individuals | A | 0.035 | ***1.00E-17*** |  | A | G | A | 0.033 | 0.019 | 0.048 | ***7.70E-06*** |
| 257 | rs4777230 | *TLE3* | 15 | 70073423 | body height | 28552196 | 1,249 whole genome sequenced European ancestry individuals, 3,541 whole genome sequenced individuals, 46,910 European ancestry individuals, 471 Carlantino (founder/genetic isolate) individuals, 1,197 Friuli Venezia Giulia (founder/genetic isolate) individuals, 1,043 Mylopotamos (founder/genetic isolate) individuals, 933 Pomak (founder/genetic isolate) individuals, 1,785 Val Borbera (founder/genetic isolate) individuals | T | 0.063 | ***8.00E-12*** |  | C | T | C | 0.032 | 0.017 | 0.046 | ***2.19E-05*** |
| 258 | rs4886782 | *LOXL1* | 15 | 73936469 | body height | 30595370 | approximately 458,000 European ancestry individuals | ? | NA | ***6.00E-82*** |  | A | G | G | 0.046 | 0.030 | 0.062 | ***3.32E-08*** |
| 259 | rs750460 | *LOXL1* | 15 | 73949165 | body height | 20881960 | 133,653 European ancestry individuals | ? | NA | ***4.00E-12*** |  | A | G | G | 0.047 | 0.031 | 0.063 | ***1.16E-08*** |
| 260 | rs8025068 | *ARID3B* | 15 | 74577704 | body height | 31562340 | 159,095 Japanese ancestry individuals | ? | 0.023 | ***2.00E-15*** |  | G | T | G | 0.023 | 0.013 | 0.032 | ***6.66E-06*** |
| 261 | rs4886707 | *SIN3A - PTPN9* | 15 | 75463126 | body height | 20189936 | 19,633 Japanese ancestry individuals | T | 0.060 | ***8.00E-08*** |  | T | C | T | 0.027 | 0.017 | 0.038 | ***3.23E-07*** |
| 262 | rs11639183 | *PTPN9* | 15 | 75515351 | body height | 31562340 | 159,095 Japanese ancestry individuals | ? | 0.037 | ***4.00E-38*** |  | C | T | C | 0.028 | 0.017 | 0.038 | ***1.69E-07*** |
| 263 | rs7184046 | *AC105036.3, PTPN9* | 15 | 75573809 | body height | 25429064 | 36,227 East Asian ancestry individuals | C | 0.030 | ***2.00E-10*** |  | C | G | C | 0.028 | 0.017 | 0.038 | ***1.88E-07*** |
| 264 | rs2401171 | *ADAMTSL3* | 15 | 83888924 | body height | 25429064 | 36,227 East Asian ancestry individuals | T | 0.052 | ***4.00E-21*** |  | T | G | G | 0.043 | 0.032 | 0.055 | ***2.24E-14*** |
| 265 | rs7183263 | *ADAMTSL3* | 15 | 83904289 | body height | 20397748 | 11,536 European ancestry individuals | G | 0.070 | ***4.00E-07*** |  | T | G | G | 0.044 | 0.033 | 0.056 | ***5.88E-15*** |
| 266 | rs11259933 | *ADAMTSL3* | 15 | 83911404 | body height | 23563607 | 8,097 European ancestry tall individuals, 8,099 European ancestry short individuals | A | 1.190 | ***1.00E-19*** |  | G | A | A | 0.044 | 0.033 | 0.055 | ***1.02E-14*** |
| 267 | rs4842838 | *ADAMTSL3* | 15 | 83913372 | body height | 20189936 | 19,633 Japanese ancestry individuals | G | 0.060 | ***1.00E-07*** |  | G | T | T | 0.045 | 0.033 | 0.056 | ***4.55E-15*** |
| 268 | rs8024628 | *ADAMTSL3* | 15 | 83917711 | body height | 28552196 | 1,249 whole genome sequenced European ancestry individuals, 3,541 whole genome sequenced individuals, 46,910 European ancestry individuals, 471 Carlantino (founder/genetic isolate) individuals, 1,197 Friuli Venezia Giulia (founder/genetic isolate) individuals, 1,043 Mylopotamos (founder/genetic isolate) individuals, 933 Pomak (founder/genetic isolate) individuals, 1,785 Val Borbera (founder/genetic isolate) individuals | A | 0.056 | ***5.00E-19*** |  | A | G | G | 0.044 | 0.033 | 0.055 | ***9.71E-15*** |
| 269 | rs2135880 | *ADAMTSL3* | 15 | 83920861 | body height | 30595370 | approximately 458,000 European ancestry individuals | ? | NA | ***3.00E-222*** |  | T | G | G | 0.044 | 0.033 | 0.055 | ***8.61E-15*** |
| 270 | rs9672558 | *FAM169B - IRAIN* | 15 | 98636976 | body height | 31562340 | 159,095 Japanese ancestry individuals | ? | 0.101 | ***7.00E-63*** |  | C | T | T | 0.078 | 0.061 | 0.094 | ***4.34E-20*** |
| 271 | rs4369638 | *ADAMTS17* | 15 | 100254158 | body height | 30595370 | approximately 458,000 European ancestry individuals | ? | NA | ***1.00E-90*** |  | C | T | C | 0.031 | 0.017 | 0.046 | ***2.00E-05*** |
| 272 | rs4467054 | *ADAMTS17* | 15 | 100255167 | body height | 31562340 | 159,095 Japanese ancestry individuals | ? | 0.027 | ***1.00E-17*** |  | G | T | G | 0.029 | 0.018 | 0.039 | ***9.87E-08*** |
| 273 | rs6499255 | *WWP2* | 16 | 69796425 | body height | 31562340 | 159,095 Japanese ancestry individuals | ? | 0.028 | ***6.00E-20*** |  | G | A | A | 0.025 | 0.016 | 0.035 | ***4.70E-07*** |
| 274 | rs4985445 | *WWP2* | 16 | 69833932 | body height | 30595370 | approximately 458,000 European ancestry individuals | ? | NA | ***2.00E-47*** |  | A | G | A | 0.026 | 0.016 | 0.036 | ***3.49E-07*** |
| 275 | rs258324 | *CDK10* | 16 | 89687847 | body height | 25429064 | 36,227 East Asian ancestry individuals | T | 0.054 | ***4.00E-23*** |  | T | G | T | 0.043 | 0.032 | 0.054 | ***1.97E-15*** |
| 276 | rs7223535 | *ATAD5* | 17 | 30884649 | body height | 30595370 | approximately 458,000 European ancestry individuals | ? | NA | ***1.00E-222*** |  | A | G | G | 0.046 | 0.031 | 0.061 | ***3.12E-09*** |
| 277 | rs35958868 | *ADAP2* | 17 | 30909727 | body height | 28552196 | 1,249 whole genome sequenced European ancestry individuals, 3,541 whole genome sequenced individuals, 46,910 European ancestry individuals, 471 Carlantino (founder/genetic isolate) individuals, 1,197 Friuli Venezia Giulia (founder/genetic isolate) individuals, 1,043 Mylopotamos (founder/genetic isolate) individuals, 933 Pomak (founder/genetic isolate) individuals, 1,785 Val Borbera (founder/genetic isolate) individuals | A | 0.060 | ***9.00E-15*** |  | A | G | G | 0.047 | 0.032 | 0.063 | ***2.72E-09*** |
| 278 | rs12948439 | *SUMO2P17* | 17 | 48889762 | body height | 30595370 | approximately 458,000 European ancestry individuals | ? | NA | ***4.00E-74*** |  | G | A | G | 0.035 | 0.024 | 0.046 | ***1.44E-10*** |
| 279 | rs318095 | *SUMO2P17* | 17 | 48897372 | body height | 25282103 | 253,288 European ancestry individuals | T | 0.024 | ***2.00E-16*** |  | T | C | T | 0.035 | 0.024 | 0.046 | ***1.47E-10*** |
| 280 | rs1057902 | *UBE2Z* | 17 | 48928226 | body height | 31562340 | 159,095 Japanese ancestry individuals | ? | 0.025 | ***1.00E-14*** |  | C | T | C | 0.034 | 0.023 | 0.045 | ***4.34E-10*** |
| 281 | rs35587648 | *ZNF652* | 17 | 49340816 | body height | 30595370 | approximately 458,000 European ancestry individuals | ? | NA | ***7.00E-42*** |  | A | G | G | 0.030 | 0.020 | 0.041 | ***1.49E-08*** |
| 282 | rs2079795 | *LINC02875 - TBX4* | 17 | 61419288 | body height | 25282103 | 253,288 European ancestry individuals | T | 0.045 | ***2.00E-46*** |  | T | C | T | 0.049 | 0.039 | 0.060 | ***6.12E-21*** |
| 283 | rs2008018 | *CSH2 - GH2* | 17 | 63877111 | body height | 31562340 | 159,095 Japanese ancestry individuals | ? | 0.041 | ***4.00E-46*** |  | G | A | G | 0.041 | 0.031 | 0.051 | ***4.55E-16*** |
| 284 | rs2070776 | *CD79B, AC127029.3* | 17 | 63930138 | body height | 25282103 | 253,288 European ancestry individuals | A | 0.042 | ***6.00E-41*** |  | G | A | G | 0.046 | 0.036 | 0.056 | ***7.34E-20*** |
| 285 | rs8098316 | *RBBP8 - CABLES1* | 18 | 23092591 | body height | 25429064 | 36,227 East Asian ancestry individuals | T | 0.052 | ***7.00E-23*** |  | T | G | G | 0.056 | 0.043 | 0.070 | ***5.07E-17*** |
| 286 | rs8096254 | *RBBP8 - CABLES1* | 18 | 23133251 | body height | 28270201 | 19,965 British ancestry individuals from 6863 families. | A | 0.005 | ***4.00E-12*** |  | G | A | A | 0.055 | 0.043 | 0.068 | ***2.98E-18*** |
| 287 | rs11082304 | *CABLES1* | 18 | 23141009 | body height | 23563607 | 8,097 European ancestry tall individuals, 8,099 European ancestry short individuals | T | 1.180 | ***5.00E-15*** |  | G | T | T | 0.038 | 0.028 | 0.048 | ***1.86E-14*** |
| 288 | rs4800148 | *CABLES1* | 18 | 23144364 | body height | 28552196 | 1,249 whole genome sequenced European ancestry individuals, 3,541 whole genome sequenced individuals, 46,910 European ancestry individuals, 471 Carlantino (founder/genetic isolate) individuals, 1,197 Friuli Venezia Giulia (founder/genetic isolate) individuals, 1,043 Mylopotamos (founder/genetic isolate) individuals, 933 Pomak (founder/genetic isolate) individuals, 1,785 Val Borbera (founder/genetic isolate) individuals | A | 0.072 | ***2.00E-19*** |  | G | A | A | 0.060 | 0.049 | 0.072 | ***5.92E-25*** |
| 289 | rs1787200 | *DYM* | 18 | 49061284 | body height | 21998595 | 20,427 African ancestry individuals | A | 0.053 | ***1.00E-10*** |  | G | A | A | 0.035 | 0.025 | 0.045 | ***1.83E-11*** |
| 290 | rs2878902 | *DYM, AC016866.2* | 18 | 49124067 | body height | 28552196 | 1,249 whole genome sequenced European ancestry individuals, 3,541 whole genome sequenced individuals, 46,910 European ancestry individuals, 471 Carlantino (founder/genetic isolate) individuals, 1,197 Friuli Venezia Giulia (founder/genetic isolate) individuals, 1,043 Mylopotamos (founder/genetic isolate) individuals, 933 Pomak (founder/genetic isolate) individuals, 1,785 Val Borbera (founder/genetic isolate) individuals | T | 0.047 | ***3.00E-10*** |  | G | T | T | 0.037 | 0.026 | 0.047 | ***2.47E-12*** |
| 291 | rs12458127 | *DYM* | 18 | 49130988 | body height | 31562340 | 159,095 Japanese ancestry individuals | ? | 0.027 | ***1.00E-19*** |  | T | C | C | 0.043 | 0.031 | 0.054 | ***3.26E-13*** |
| 292 | rs74494415 | *GALR1* | 18 | 77260182 | body height | 31562340 | 159,095 Japanese ancestry individuals | ? | 0.031 | ***1.00E-09*** |  | T | C | C | 0.040 | 0.022 | 0.058 | ***8.97E-06*** |
| 293 | rs59951000 | *GALR1* | 18 | 77263872 | body height | 30595370 | approximately 458,000 European ancestry individuals | ? | NA | ***4.00E-14*** |  | T | C | C | 0.039 | 0.022 | 0.057 | ***1.16E-05*** |
| 294 | rs1560710 | *SLC44A2* | 19 | 10631767 | body height | 31562340 | 159,095 Japanese ancestry individuals | ? | 0.040 | ***2.00E-38*** |  | C | T | C | 0.038 | 0.028 | 0.049 | ***7.82E-13*** |
| 295 | rs7250071 | *ILF3* | 19 | 10655143 | body height | 25429064 | 36,227 East Asian ancestry individuals | T | 0.042 | ***2.00E-16*** |  | C | T | C | 0.036 | 0.026 | 0.047 | ***1.71E-12*** |
| 296 | rs12459943 | *DNM2* | 19 | 10748832 | body height | 25429064 | 36,227 East Asian ancestry individuals | A | 0.030 | ***5.00E-11*** |  | A | G | G | 0.033 | 0.023 | 0.044 | ***4.11E-10*** |
| 297 | rs7264113 | *AL356414.1 - SMOX* | 20 | 4110155 | body height | 31562340 | 159,095 Japanese ancestry individuals | ? | 0.038 | ***1.00E-16*** |  | A | G | A | 0.033 | 0.020 | 0.047 | ***1.87E-06*** |
| 298 | rs1741344 | *SMOX* | 20 | 4121153 | body height | 20881960 | 133,653 European ancestry individuals | T | 0.023 | ***3.00E-09*** |  | C | T | C | 0.032 | 0.019 | 0.044 | ***3.37E-07*** |
| 299 | rs967417 | *CASC20 - LINC01713* | 20 | 6640246 | body height | 18391951 | 30,968 European ancestry individuals | C | 4.300 | ***2.00E-08*** |  | G | A | G | 0.033 | 0.020 | 0.046 | ***1.10E-06*** |
| 300 | rs3213180 | *E2F1* | 20 | 33675818 | body height | 31562340 | 159,095 Japanese ancestry individuals | ? | 0.023 | ***5.00E-13*** |  | C | G | G | 0.032 | 0.022 | 0.043 | ***1.83E-09*** |
| 301 | rs6058227 | *UQCC1* | 20 | 35308144 | body height | 28552196 | 1,249 whole genome sequenced European ancestry individuals, 3,541 whole genome sequenced individuals, 46,910 European ancestry individuals, 471 Carlantino (founder/genetic isolate) individuals, 1,197 Friuli Venezia Giulia (founder/genetic isolate) individuals, 1,043 Mylopotamos (founder/genetic isolate) individuals, 933 Pomak (founder/genetic isolate) individuals, 1,785 Val Borbera (founder/genetic isolate) individuals | T | 0.066 | ***2.00E-09*** |  | T | C | T | 0.076 | 0.063 | 0.089 | ***9.43E-30*** |
| 302 | rs6060369 | *UQCC1* | 20 | 35319358 | body height | 18391950 | 15,821 European ancestry individuals | C | 0.440 | ***1.00E-16*** |  | C | T | C | 0.070 | 0.059 | 0.081 | ***4.42E-36*** |
| 303 | rs6088792 | *UQCC1* | 20 | 35321981 | body height | 18391951 | 30,968 European ancestry individuals | T | 4.700 | ***8.00E-07*** |  | T | C | T | 0.036 | 0.020 | 0.052 | ***8.18E-06*** |
| 304 | rs6060373 | *UQCC1* | 20 | 35326405 | body height | 18391952 | 13,665 European ancestry individuals | A | 0.080 | ***2.00E-17*** |  | G | A | G | 0.069 | 0.059 | 0.080 | ***5.99E-36*** |
| 305 | rs6088813 | *UQCC1* | 20 | 35387378 | body height | 19343178 | 12,611 European ancestry individuals | ? | 0.090 | ***1.00E-13*** |  | C | A | C | 0.072 | 0.061 | 0.083 | ***1.45E-38*** |
| 306 | rs224329 | *UQCC1 - GDF5-AS1* | 20 | 35431781 | body height | 25429064 | 36,227 East Asian ancestry individuals | T | 0.064 | ***2.00E-30*** |  | T | C | T | 0.074 | 0.063 | 0.085 | ***5.17E-40*** |
| 307 | rs224333 | *GDF5* | 20 | 35436182 | body height | 23563607 | 8,097 European ancestry tall individuals, 8,099 European ancestry short individuals | A | 1.310 | ***8.00E-37*** |  | A | G | A | 0.074 | 0.063 | 0.084 | ***7.96E-40*** |
| 308 | rs143384 | *GDF5* | 20 | 35437976 | body weights and measures, body height | 30166351 | 4,988 Spanish ancestry individuals | G | 0.064 | ***9.00E-292*** |  | G | A | G | 0.074 | 0.063 | 0.085 | ***3.61E-40*** |
| 309 | rs57545942 | *FER1L4* | 20 | 35595557 | body height | 28552196 | 1,249 whole genome sequenced European ancestry individuals, 3,541 whole genome sequenced individuals, 46,910 European ancestry individuals, 471 Carlantino (founder/genetic isolate) individuals, 1,197 Friuli Venezia Giulia (founder/genetic isolate) individuals, 1,043 Mylopotamos (founder/genetic isolate) individuals, 933 Pomak (founder/genetic isolate) individuals, 1,785 Val Borbera (founder/genetic isolate) individuals | T | 0.051 | ***8.00E-09*** |  | T | C | T | 0.062 | 0.048 | 0.077 | ***5.58E-18*** |
| 310 | rs2235363 | *ZHX3, PLCG1* | 20 | 41179129 | body height | 31562340 | 159,095 Japanese ancestry individuals | ? | 0.018 | ***5.00E-10*** |  | G | A | G | 0.023 | 0.013 | 0.032 | ***7.22E-06*** |
| 311 | rs11537645 | *UBE2C* | 20 | 45812764 | body height | 31562340 | 159,095 Japanese ancestry individuals | ? | 0.031 | ***4.00E-12*** |  | G | C | C | 0.044 | 0.026 | 0.061 | ***1.45E-06*** |
| 312 | rs4821083 | *SYN3* | 22 | 32660355 | body height | 20881960 | 133,653 European ancestry individuals | T | 0.031 | ***3.00E-10*** |  | T | C | T | 0.053 | 0.042 | 0.064 | ***4.91E-21*** |
| 313 | rs2413143 | *SYN3* | 22 | 32660873 | body height | 25282103 | 253,288 European ancestry individuals | T | 0.034 | ***2.00E-17*** |  | C | T | C | 0.053 | 0.042 | 0.064 | ***9.70E-21*** |
| NHGRI, National Human Genome Research Institute (https://www.genome.gov/). | | | | | | | | | | | | | | | | | | |
| *P*-value (*p* < 0.05/1722 SNPs) was highlighted in bold italic. | | | | | | | | | | | | | | | | | | |
| Abbreviations: SNP, single nucleotide polymorphism; GWAS, genome-wide association study; No., number; Chr., chromosome; 95% CI, 95% confidence interval; NA, not available. | | | | | | | | | | | | | | | | | | |
