# Supplementary figures and images for: Your height affects your health: genetic determinants and health-related outcomes in Taiwan
Source: BMC Med. 2022 Jul 13;20:250. doi: 10.1186/s12916-022-02450-w (PMC9281111; doi:10.1186/s12916-022-02450-w)

**Additional file 4: Fig. S1**


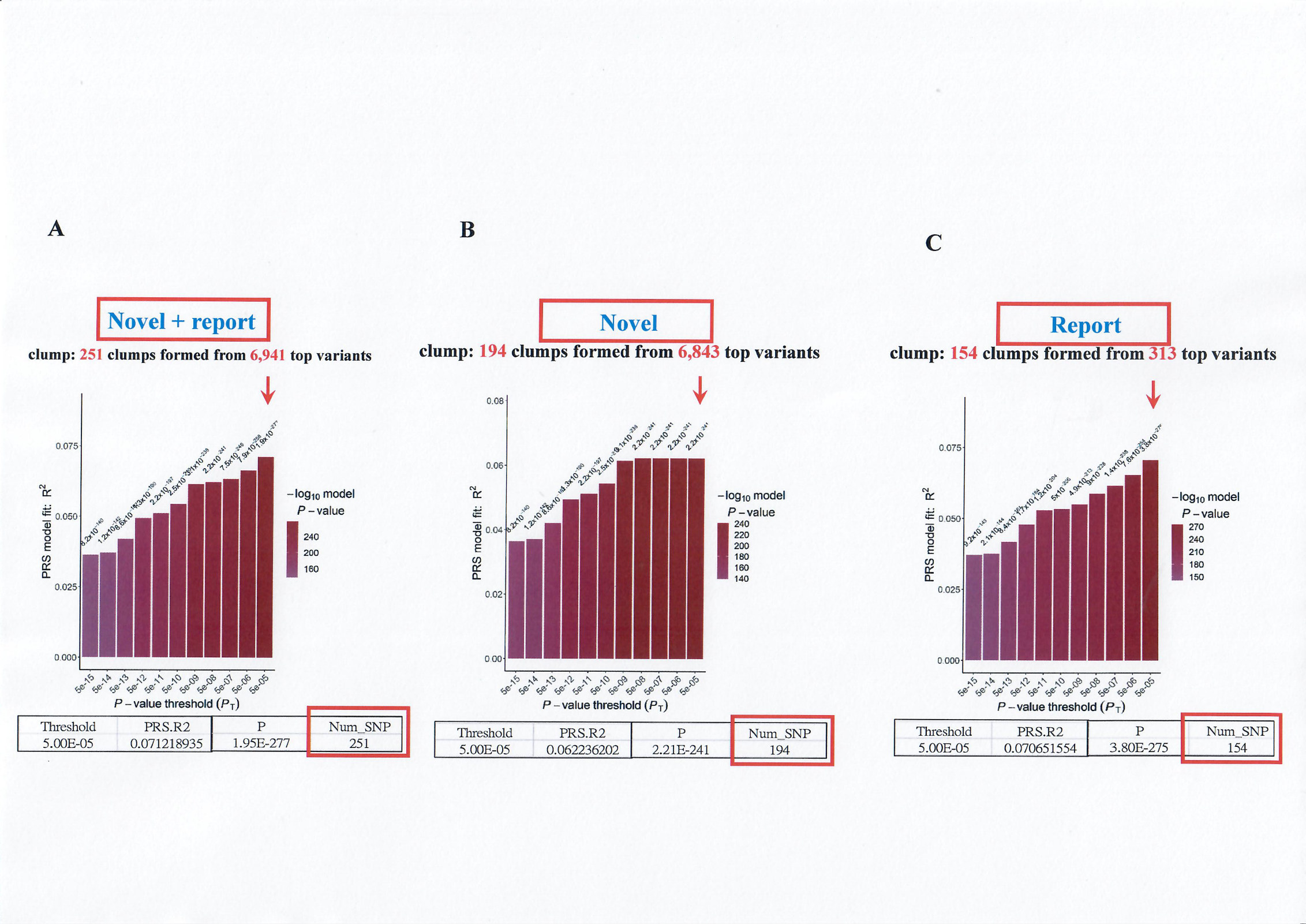


**Additional file 4: Fig. S2**


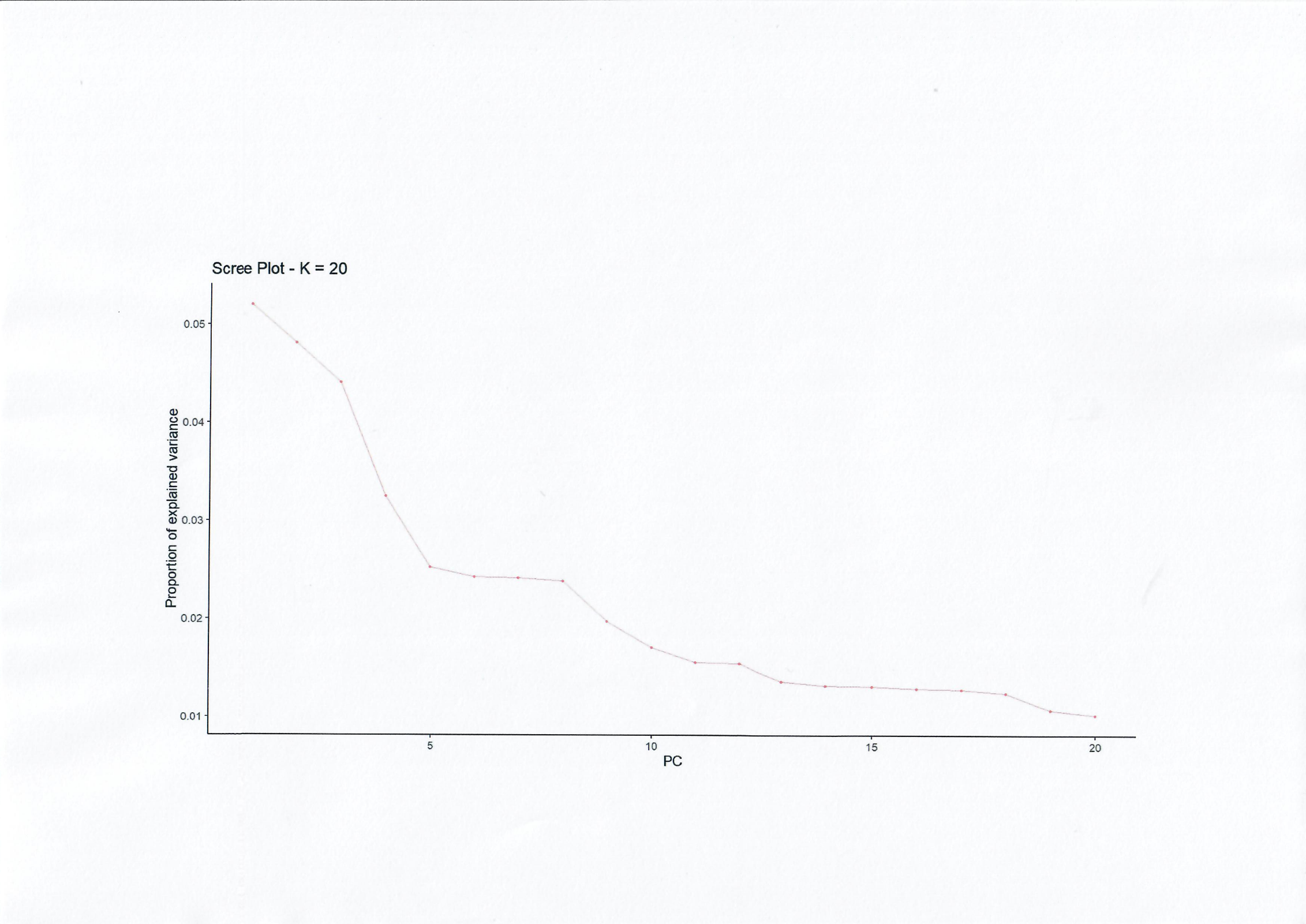


**Additional file 4: Fig. S3**

**
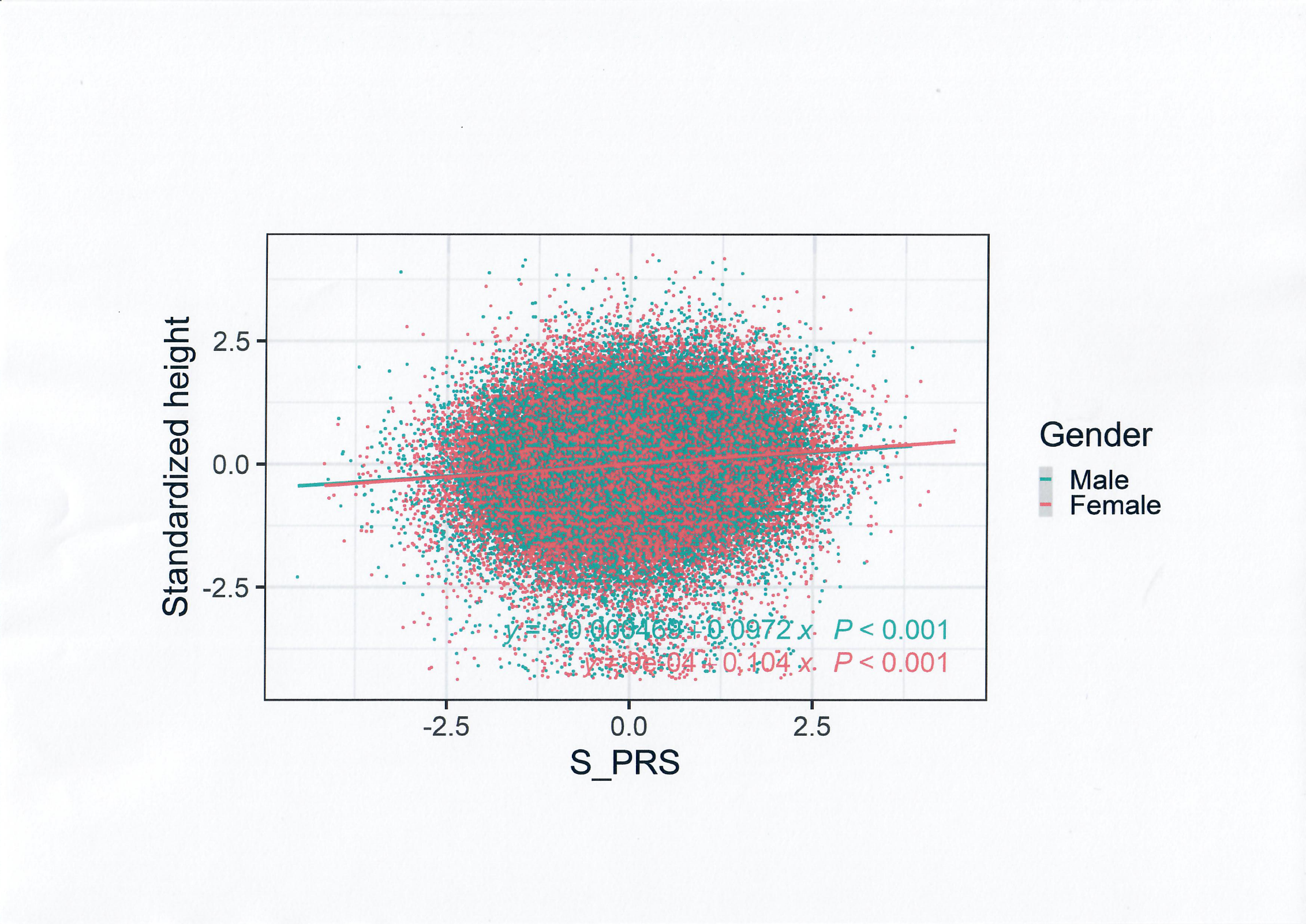
**

Supplement: Supplementary file 4 — Additional file 4: Figure S1. The clumping and p-value threshold method identifies the “best-fit” SNP number for the polygenic risk score (PRS) calculation, according to the largest explainable phenotype correlation r2 using only PRS (PRS r2 and SNP number). The x-axis shows the p-value thresholds from the height of GWAS results. The y-axis represents the explainable phenotypic correlation r2 using only the PRS (PRS r2). The p-values above the bars show the statistical significance of the associations between genetically determined height (PRS) and measured height (phenotype). (A) 251 SNPs were obtained from 6,941 SNPs (novel and reported SNPs; PRS r2 = 0.0712, SNP number = 251). (B) 194 SNPs were obtained from 6,843 SNPs (novel SNPs; PRS r2 = 0.0622, SNP number = 194). (C) 154 SNPs were obtained from 313 SNPs (reported SNPs; PRS r2 = 0.0706, SNP number = 154). Figure S2. Scree plot identifying the number of principal component analyses (PCA) needed for the correction of population structure in the height GWAS study, using pcadapt (an R package used to determine the number of principal components). Figure S3. Association between genetically determined height (PRS237) and measured height (phenotype) in an independent cohort of the Big Data Center in China Medical University Hospital in Taiwan. The measured height (cm) and calculated polygenic risk score (PRS) for height were stratified by sex, mean-centered, and normalized to one standard deviation (SD; males, N = 46,310; females, N = 54,728). The normalized measured height is represented on the y-axis, and normalized genetically determined height (PRS237) is represented on the x-axis. [file 12916_2022_2450_MOESM4_ESM.docx]
