# Supplementary material for: Your height affects your health: genetic determinants and health-related outcomes in Taiwan
Source: BMC Med. 2022 Jul 13;20:250. doi: 10.1186/s12916-022-02450-w (PMC9281111; doi:10.1186/s12916-022-02450-w)
Supplement: Supplementary file 5 — Additional file 5: Table S4. Characteristics of 237 out of the 251 SNPs associated with height in the independent cohort of the Big Data Center at China Medical University Hospital in Taiwan. [file 12916_2022_2450_MOESM5_ESM.docx]

| **Additional file 5: Table S4** Characteristics of 237 out of the 251 SNPs associated with height in the independent cohort of the Big Data Center at China Medical University Hospital in Taiwan | | | | | | | | | | | |
| --- | --- | --- | --- | --- | --- | --- | --- | --- | --- | --- | --- |
| **No.** | **rs ID** | **Gene** | **Chr.** | **Position** | **Minor allele** | **Major allele** | **Risk allele** | **Independent cohort of the Big Data Center in China Medical University Hospital in Taiwan (N = 101,038)** | | | |
|  |  |  |  |  |  |  |  | **Beta** | **95% CI** | | ***P*-value** |
| 1 | rs2300092 | *MTOR* | 1 | 11206407 | T | C | T | -0.019 | -0.029 | -0.009 | 1.47E-04 |
| 2 | rs56265117 | *MFAP2* | 1 | 16980428 | C | T | C | -0.032 | -0.040 | -0.024 | 3.32E-15 |
| 3 | rs3014240 | *CCDC17* | 1 | 45623553 | C | G | C | -0.016 | -0.025 | -0.008 | 7.40E-05 |
| 4 | rs76910682 |  | 1 | 50408548 | A | G | A | 0.024 | 0.014 | 0.034 | 3.48E-06 |
| 5 | rs12098132 | *FAF1* | 1 | 50661852 | A | C | A | 0.049 | 0.035 | 0.062 | 4.57E-13 |
| 6 | rs61115731 | *FAF1* | 1 | 50932429 | C | G | C | 0.050 | 0.037 | 0.063 | 2.56E-14 |
| 7 | rs140830175 | *LINC01562* | 1 | 51197120 | T | C | T | 0.043 | 0.029 | 0.056 | 6.29E-10 |
| 8 | rs4926705 |  | 1 | 55953858 | A | G | A | 0.033 | 0.022 | 0.044 | 1.18E-08 |
| 9 | rs2666504 |  | 1 | 62169409 | C | T | C | 0.012 | 0.004 | 0.021 | 2.56E-03 |
| 10 | rs3806340 | *PKN2-AS1* | 1 | 88683110 | G | T | T | -0.015 | -0.023 | -0.007 | 2.42E-04 |
| 11 | rs7530513 | *KYAT3* | 1 | 88944228 | A | G | G | 0.007 | -0.001 | 0.015 | 8.98E-02 |
| 12 | rs7513580 |  | 1 | 118307286 | A | G | G | 0.040 | 0.032 | 0.048 | 3.18E-21 |
| 13 | rs11205303 | *MTMR11* | 1 | 149934520 | C | T | C | 0.041 | 0.033 | 0.049 | 6.67E-23 |
| 14 | rs6587515 |  | 1 | 150636412 | A | G | A | 0.011 | 0.001 | 0.020 | 2.40E-02 |
| 15 | rs10489289 | *DNM3* | 1 | 172254949 | C | T | C | 0.027 | 0.018 | 0.036 | 9.00E-09 |
| 16 | rs1325596 | *PAPPA2* | 1 | 176824930 | G | A | A | -0.021 | -0.030 | -0.012 | 1.17E-05 |
| 17 | rs10911212 | *LAMC1* | 1 | 183055334 | C | T | C | -0.004 | -0.012 | 0.004 | 3.47E-01 |
| 18 | rs12047271 |  | 1 | 184044357 | C | T | C | 0.033 | 0.025 | 0.041 | 4.82E-16 |
| 19 | rs4472734 | *PTPN14* | 1 | 214444842 | C | T | C | -0.027 | -0.035 | -0.019 | 1.64E-11 |
| 20 | rs1046017 | *TGFB2, TGFB2-OT1* | 1 | 218443793 | C | G | G | -0.030 | -0.039 | -0.022 | 1.52E-11 |
| 21 | rs7538503 |  | 1 | 219615188 | G | A | G | 0.013 | 0.004 | 0.023 | 5.87E-03 |
| 22 | rs10165255 | *CYS1* | 2 | 10059474 | A | G | A | -0.019 | -0.030 | -0.008 | 8.88E-04 |
| 23 | rs6735681 |  | 2 | 15983051 | T | C | T | -0.007 | -0.015 | 0.001 | 9.25E-02 |
| 24 | rs780094 | *GCKR* | 2 | 27518370 | T | C | C | -0.013 | -0.021 | -0.005 | 1.26E-03 |
| 25 | rs2367623 | *LTBP1* | 2 | 33202983 | A | C | A | 0.015 | 0.007 | 0.023 | 2.68E-04 |
| 26 | rs143098957 | *LTBP1* | 2 | 33263857 | T | G | G | 0.042 | 0.031 | 0.052 | 3.79E-15 |
| 27 | rs3755206 | *CRIM1* | 2 | 36456285 | G | T | T | 0.031 | 0.021 | 0.041 | 2.60E-09 |
| 28 | rs17019115 | *FEZ2* | 2 | 36575874 | C | G | G | 0.014 | 0.006 | 0.022 | 5.01E-04 |
| 29 | rs4670703 |  | 2 | 37385957 | C | A | C | -0.016 | -0.024 | -0.008 | 7.54E-05 |
| 30 | rs6544743 | *LOC102723904* | 2 | 44163230 | T | G | T | -0.016 | -0.025 | -0.008 | 2.08E-04 |
| 31 | rs79121675 |  | 2 | 55724807 | A | C | A | 0.033 | 0.010 | 0.056 | 4.17E-03 |
| 32 | rs146446706 | *LOC112268416, EFEMP1* | 2 | 55870880 | T | C | T | 0.093 | 0.060 | 0.127 | 4.72E-08 |
| 33 | rs3791675 | *EFEMP1* | 2 | 55884174 | C | T | C | -0.060 | -0.069 | -0.051 | 6.17E-37 |
| 34 | rs1432559 | *LOC105374690* | 2 | 55962483 | G | T | G | 0.035 | 0.016 | 0.053 | 2.87E-04 |
| 35 | rs4241349 | *ANTXR1* | 2 | 69103152 | G | A | G | -0.020 | -0.029 | -0.011 | 1.13E-05 |
| 36 | rs1824305 |  | 2 | 71179325 | T | C | C | -0.030 | -0.038 | -0.022 | 3.21E-13 |
| 37 | rs57092473 |  | 2 | 71440419 | A | G | G | -0.035 | -0.043 | -0.027 | 4.41E-18 |
| 38 | rs1913671 | *EIF2AK3* | 2 | 88600365 | T | C | C | -0.022 | -0.030 | -0.014 | 7.03E-08 |
| 39 | rs1118150 | *DIRC3* | 2 | 217415545 | A | C | A | 0.026 | 0.017 | 0.035 | 3.11E-08 |
| 40 | rs484085 | *USP37* | 2 | 218531961 | C | T | T | -0.026 | -0.035 | -0.016 | 7.66E-08 |
| 41 | rs422702 | *CFAP65* | 2 | 219037931 | C | T | C | 0.038 | 0.031 | 0.046 | 8.41E-22 |
| 42 | rs76709099 | *IHH* | 2 | 219055182 | A | C | C | 0.052 | 0.037 | 0.067 | 4.68E-12 |
| 43 | rs374935766 |  | 2 | 231850100 | C | G | G | 0.084 | 0.038 | 0.130 | 3.73E-04 |
| 44 | rs33994242 |  | 2 | 231915948 | G | A | A | 0.036 | 0.024 | 0.048 | 3.93E-09 |
| 45 | rs76803230 | *DIS3L2* | 2 | 232063990 | G | T | T | 0.054 | 0.047 | 0.062 | 7.52E-42 |
| 46 | rs146229392 | *DIS3L2* | 2 | 232064573 | C | G | G | 0.054 | 0.030 | 0.079 | 1.52E-05 |
| 47 | rs3748967 | *DIS3L2* | 2 | 232333663 | A | G | G | 0.043 | 0.035 | 0.051 | 1.19E-26 |
| 48 | rs894857163 | *GIGYF2* | 2 | 232726985 | T | C | C |  |  |  |  |
| 49 | rs11130111 | *CCDC12* | 3 | 46968315 | C | T | C | -0.021 | -0.029 | -0.014 | 1.00E-07 |
| 50 | rs12495173 | *KIF9-AS1, KIF9* | 3 | 47241409 | T | C | T | 0.025 | 0.014 | 0.036 | 8.77E-06 |
| 51 | rs1209842003 |  | 3 | 52186981 | T | G | G |  |  |  |  |
| 52 | rs754871503 | *NT5DC2* | 3 | 52525012 | C | T | T |  |  |  |  |
| 53 | rs1328122506 | *SFMBT1* | 3 | 52913981 | A | C | C |  |  |  |  |
| 54 | rs2564923 |  | 3 | 53069246 | A | G | A | -0.026 | -0.035 | -0.017 | 6.48E-09 |
| 55 | rs13086339 | *RYBP* | 3 | 72428668 | C | A | C | 0.024 | 0.016 | 0.032 | 5.19E-09 |
| 56 | rs11710894 | *BOC* | 3 | 113273112 | T | C | C | 0.012 | 0.004 | 0.020 | 2.54E-03 |
| 57 | rs4073154 | *H1FX-AS1* | 3 | 129316642 | A | G | G | -0.028 | -0.036 | -0.019 | 1.25E-10 |
| 58 | rs7632556 |  | 3 | 134087102 | A | G | G | 0.022 | 0.013 | 0.030 | 3.92E-07 |
| 59 | rs9841212 |  | 3 | 134473096 | C | T | T | -0.024 | -0.034 | -0.015 | 1.33E-07 |
| 60 | rs57345461 | *ZBTB38* | 3 | 141407983 | T | A | T | 0.054 | 0.046 | 0.062 | 3.48E-37 |
| 61 | rs1055153 | *WWTR1* | 3 | 149657086 | T | G | G | 0.024 | 0.012 | 0.036 | 1.04E-04 |
| 62 | rs1104288 | *RSRC1* | 3 | 158249730 | A | C | C | -0.019 | -0.027 | -0.011 | 7.46E-06 |
| 63 | rs12639337 | *FNDC3B* | 3 | 172279149 | G | C | C | -0.027 | -0.035 | -0.019 | 3.93E-11 |
| 64 | rs6774762 | *GHSR* | 3 | 172447200 | G | A | A | 0.030 | 0.020 | 0.040 | 1.25E-08 |
| 65 | rs9790124 | *RTP2, LOC100131635* | 3 | 187712899 | G | A | G | -0.027 | -0.036 | -0.017 | 1.86E-08 |
| 66 | rs116972792 | *FAM184B* | 4 | 17648794 | A | G | G | 0.058 | 0.036 | 0.081 | 4.86E-07 |
| 67 | rs16895971 | *LCORL* | 4 | 17883363 | C | T | T | 0.066 | 0.058 | 0.075 | 1.27E-51 |
| 68 | rs16896140 | *LCORL* | 4 | 17957655 | C | T | C | 0.053 | 0.034 | 0.073 | 9.97E-08 |
| 69 | rs2724485 | *LCORL* | 4 | 17968075 | C | T | T | 0.019 | 0.011 | 0.027 | 5.31E-06 |
| 70 | rs148309730 |  | 4 | 18085973 | G | A | A | 0.052 | 0.029 | 0.075 | 1.15E-05 |
| 71 | rs76924442 |  | 4 | 18118006 | A | G | G | 0.045 | 0.031 | 0.060 | 1.16E-09 |
| 72 | rs4698216 |  | 4 | 18128100 | T | C | T | 0.021 | 0.013 | 0.029 | 3.22E-07 |
| 73 | rs56281640 |  | 4 | 56899650 | A | G | A | 0.026 | 0.018 | 0.034 | 2.79E-10 |
| 74 | rs10027494 | *ADAMTS3* | 4 | 72541929 | A | T | A | 0.025 | 0.015 | 0.034 | 2.04E-07 |
| 75 | rs7697556 |  | 4 | 72649596 | C | T | T | 0.035 | 0.027 | 0.043 | 4.68E-18 |
| 76 | rs1662840 |  | 4 | 81235255 | T | C | T | 0.043 | 0.032 | 0.053 | 5.06E-16 |
| 77 | rs17017911 | *GUSBP5* | 4 | 143559481 | G | A | A | -0.020 | -0.028 | -0.012 | 8.76E-07 |
| 78 | rs6845999 | *HHIP-AS1* | 4 | 144644674 | T | C | T | 0.051 | 0.041 | 0.060 | 6.33E-26 |
| 79 | rs117072351 | *HHIP* | 4 | 144676679 | T | C | T | 0.028 | -0.002 | 0.058 | 6.60E-02 |
| 80 | rs4240326 |  | 4 | 144918112 | A | G | A | -0.022 | -0.031 | -0.014 | 7.24E-07 |
| 81 | rs301901 | *NIPBL* | 5 | 37046524 | A | G | A | -0.014 | -0.022 | -0.006 | 4.75E-04 |
| 82 | rs12654242 |  | 5 | 42365278 | G | A | G | -0.035 | -0.048 | -0.023 | 6.78E-08 |
| 83 | rs4273617 | *GHR* | 5 | 42695369 | G | A | G | -0.036 | -0.047 | -0.025 | 4.54E-11 |
| 84 | rs4865956 |  | 5 | 55586677 | T | A | T | -0.016 | -0.024 | -0.008 | 1.31E-04 |
| 85 | rs6453386 | *SCAMP1* | 5 | 78408512 | C | G | C | -0.021 | -0.030 | -0.012 | 2.45E-06 |
| 86 | rs985296 | *MEF2C-AS1* | 5 | 89081827 | A | G | G | 0.019 | 0.011 | 0.027 | 2.58E-06 |
| 87 | rs7706662 | *CEP120* | 5 | 123419868 | T | C | C | 0.014 | 0.006 | 0.022 | 4.85E-04 |
| 88 | rs2908532 |  | 5 | 142242319 | A | C | A | 0.019 | 0.010 | 0.027 | 1.67E-05 |
| 89 | rs184923695 | *ARHGAP26* | 5 | 143097754 | A | G | G | 0.018 | 0.003 | 0.032 | 1.74E-02 |
| 90 | rs2974438 | *SLIT3* | 5 | 168823898 | A | G | G | 0.018 | 0.008 | 0.029 | 4.00E-04 |
| 91 | rs12153391 | *SMIM23* | 5 | 171776434 | A | C | C | 0.031 | 0.023 | 0.039 | 3.49E-14 |
| 92 | rs4868126 |  | 5 | 171856465 | T | G | G | -0.025 | -0.033 | -0.017 | 1.58E-09 |
| 93 | rs722585 | *GMDS, HCG17* | 6 | 1775629 | A | G | G | 0.012 | 0.003 | 0.020 | 7.51E-03 |
| 94 | rs186405009 | *SLC17A1* | 6 | 25823049 | A | G | A | 0.048 | 0.003 | 0.093 | 3.64E-02 |
| 95 | rs811041 |  | 6 | 26225804 | C | G | C | -0.028 | -0.037 | -0.019 | 2.21E-09 |
| 96 | rs181680390 | *BTN3A3* | 6 | 26452046 | A | T | A | 0.070 | 0.032 | 0.108 | 3.29E-04 |
| 97 | rs185780403 |  | 6 | 26745946 | A | C | A |  |  |  |  |
| 98 | rs192632187 |  | 6 | 27113326 | C | T | C |  |  |  |  |
| 99 | rs183108303 | *ZNF204P* | 6 | 27370053 | T | G | T | 0.075 | 0.037 | 0.113 | 1.16E-04 |
| 100 | rs182819650 |  | 6 | 27823957 | C | T | C |  |  |  |  |
| 101 | rs182706663 |  | 6 | 28116420 | T | C | T |  |  |  |  |
| 102 | rs78566116 |  | 6 | 32428369 | T | G | G | 0.024 | 0.011 | 0.038 | 4.37E-04 |
| 103 | rs2780226 |  | 6 | 34231315 | C | T | C | -0.078 | -0.091 | -0.066 | 4.24E-34 |
| 104 | rs2299870 | *PPARD* | 6 | 35417160 | G | C | G | 0.019 | 0.001 | 0.037 | 4.23E-02 |
| 105 | rs1564926 | *CD2AP* | 6 | 47502410 | C | T | C | 0.012 | 0.003 | 0.021 | 6.61E-03 |
| 106 | rs12209223 | *FILIP1, LOC101928540* | 6 | 75454873 | A | C | A | 0.039 | 0.025 | 0.053 | 2.29E-08 |
| 107 | rs648831 | *BCKDHB* | 6 | 80246491 | C | T | T | -0.017 | -0.025 | -0.009 | 2.11E-05 |
| 108 | rs3805859 | *BCKDHB* | 6 | 80339229 | C | A | C | -0.012 | -0.019 | -0.004 | 4.19E-03 |
| 109 | rs145101575 | *BCKDHB* | 6 | 80344040 | A | G | A | 0.041 | 0.024 | 0.057 | 9.13E-07 |
| 110 | rs62424499 |  | 6 | 80678133 | T | A | A | 0.020 | 0.011 | 0.029 | 5.48E-06 |
| 111 | rs1145861 |  | 6 | 80940193 | C | G | G | 0.021 | 0.012 | 0.030 | 5.57E-06 |
| 112 | rs13197753 |  | 6 | 104924810 | G | C | G | -0.032 | -0.040 | -0.023 | 6.71E-14 |
| 113 | rs78638402 |  | 6 | 129995451 | G | C | C | 0.046 | 0.030 | 0.061 | 1.88E-08 |
| 114 | rs113898003 | *L3MBTL3* | 6 | 130020090 | C | T | T | 0.030 | 0.022 | 0.038 | 5.02E-13 |
| 115 | rs6926186 | *L3MBTL3* | 6 | 130029149 | G | A | G | -0.037 | -0.052 | -0.022 | 9.94E-07 |
| 116 | rs7765757 | *EPB41L2* | 6 | 131050608 | C | T | T | -0.027 | -0.040 | -0.015 | 1.43E-05 |
| 117 | rs1040525 | *ADGRG6* | 6 | 142382532 | T | C | C | 0.030 | 0.022 | 0.038 | 4.61E-13 |
| 118 | rs73780873 | *ESR1* | 6 | 151829789 | A | G | A | 0.039 | 0.030 | 0.048 | 1.08E-17 |
| 119 | rs3020359 | *ESR1* | 6 | 152044128 | T | C | C | -0.021 | -0.029 | -0.013 | 3.85E-07 |
| 120 | rs73029259 | *LOC107986666* | 6 | 163690316 | A | T | A | 0.016 | 0.003 | 0.030 | 1.72E-02 |
| 121 | rs1182176 | *GNA12* | 7 | 2834967 | G | A | A | 0.032 | 0.022 | 0.043 | 7.97E-10 |
| 122 | rs57246313 |  | 7 | 25850077 | A | G | A | 0.011 | 0.003 | 0.019 | 8.06E-03 |
| 123 | rs185053690 | *KBTBD2* | 7 | 32868716 | T | G | G | 0.108 | 0.071 | 0.145 | 1.32E-08 |
| 124 | rs2960429 | *LOC102723446, LOC105375264* | 7 | 46008459 | G | C | C | -0.026 | -0.033 | -0.018 | 2.24E-10 |
| 125 | rs1007358 |  | 7 | 46161757 | G | A | G | 0.025 | 0.015 | 0.036 | 3.29E-06 |
| 126 | rs62452707 |  | 7 | 46530573 | T | A | T | -0.020 | -0.028 | -0.012 | 4.13E-07 |
| 127 | rs42377 | *CDK6* | 7 | 92614358 | A | G | A | 0.023 | 0.010 | 0.036 | 5.34E-04 |
| 128 | rs445 | *CDK6* | 7 | 92779056 | T | C | C | 0.026 | 0.018 | 0.034 | 7.40E-10 |
| 129 | rs76364830 | *DLC1* | 8 | 13514611 | A | G | G | 0.027 | 0.013 | 0.042 | 2.81E-04 |
| 130 | rs6557667 | *LOXL2* | 8 | 23390501 | C | T | C | 0.012 | 0.003 | 0.021 | 1.12E-02 |
| 131 | rs74476179 | *EXTL3* | 8 | 28740152 | A | G | G | 0.075 | 0.048 | 0.101 | 2.77E-08 |
| 132 | rs10957084 | *LOC105375821* | 8 | 48444248 | G | A | G | 0.014 | 0.005 | 0.023 | 1.75E-03 |
| 133 | rs10958476 | *PLAG1* | 8 | 56183249 | C | T | C | 0.023 | 0.013 | 0.032 | 6.41E-06 |
| 134 | rs181231559 | *PLAG1* | 8 | 56188663 | C | T | T | 0.091 | 0.041 | 0.140 | 3.50E-04 |
| 135 | rs6984782 |  | 8 | 56223330 | C | T | T | 0.060 | 0.045 | 0.075 | 1.96E-15 |
| 136 | rs112083368 |  | 8 | 56438778 | G | C | C | 0.024 | 0.009 | 0.040 | 2.50E-03 |
| 137 | rs7842996 |  | 8 | 77194904 | A | T | A | 0.033 | 0.023 | 0.043 | 5.11E-11 |
| 138 | rs7817087 |  | 8 | 116552698 | A | G | G | 0.024 | 0.016 | 0.032 | 2.69E-09 |
| 139 | rs6992491 |  | 8 | 128185657 | G | C | G | 0.022 | 0.014 | 0.030 | 7.16E-08 |
| 140 | rs3886938 | *GSDMC* | 8 | 129725300 | T | G | T | -0.026 | -0.034 | -0.017 | 1.17E-08 |
| 141 | rs566313810 |  | 8 | 134043298 | T | C | T |  |  |  |  |
| 142 | rs1213791479 |  | 8 | 134354346 | C | T | C |  |  |  |  |
| 143 | rs368372931 | *ZFAT* | 8 | 134609000 | G | A | G | 0.076 | 0.044 | 0.109 | 3.48E-06 |
| 144 | rs1246647183 | *ZFAT* | 8 | 134627377 | A | T | A |  |  |  |  |
| 145 | rs12541381 | *ZFAT* | 8 | 134637605 | A | G | G | 0.026 | 0.017 | 0.034 | 3.81E-09 |
| 146 | rs56119276 |  | 9 | 95518374 | C | T | C | 0.018 | 0.009 | 0.026 | 2.70E-05 |
| 147 | rs10120219 | *LOC105376158* | 9 | 95602265 | C | T | T | -0.022 | -0.030 | -0.014 | 5.16E-08 |
| 148 | rs4743291 |  | 9 | 95758524 | T | A | A | 0.016 | 0.007 | 0.026 | 8.08E-04 |
| 149 | rs34575265 |  | 9 | 106181520 | T | C | C | 0.019 | 0.011 | 0.027 | 5.44E-06 |
| 150 | rs7858562 | *ZNF483, PTGR1* | 9 | 111562668 | G | A | A | 0.015 | 0.006 | 0.024 | 1.27E-03 |
| 151 | rs12344818 |  | 9 | 115728289 | T | C | C | 0.021 | 0.012 | 0.030 | 1.00E-05 |
| 152 | rs3789280 | *PAPPA* | 9 | 116191093 | A | T | A | 0.017 | 0.004 | 0.030 | 1.03E-02 |
| 153 | rs10985794 |  | 9 | 122844338 | C | T | T | 0.031 | 0.020 | 0.041 | 3.97E-09 |
| 154 | rs10901208 | *FUBP3* | 9 | 130587253 | T | C | T | -0.027 | -0.035 | -0.020 | 6.93E-12 |
| 155 | rs12338076 | *QSOX2* | 9 | 136229894 | C | A | C | 0.035 | 0.027 | 0.044 | 1.10E-15 |
| 156 | rs35859988 | *CCDC3* | 10 | 12902646 | T | C | C | 0.025 | 0.015 | 0.036 | 1.90E-06 |
| 157 | rs10998375 | *TET1* | 10 | 68665362 | A | G | G | 0.021 | 0.009 | 0.032 | 5.81E-04 |
| 158 | rs779933 | *ZMIZ1* | 10 | 79158760 | A | G | G | 0.024 | 0.015 | 0.033 | 1.18E-07 |
| 159 | rs4979861 |  | 10 | 79371839 | A | G | A | -0.026 | -0.034 | -0.018 | 1.10E-09 |
| 160 | rs2648725 | *PCGF5* | 10 | 91255322 | A | T | A | 0.026 | 0.010 | 0.042 | 1.58E-03 |
| 161 | rs291979 | *GRK5* | 10 | 119370285 | A | G | A | 0.012 | 0.003 | 0.022 | 1.01E-02 |
| 162 | rs1003484 | *IGF2, INS-IGF2* | 11 | 2146388 | G | A | G | 0.031 | 0.023 | 0.039 | 4.26E-14 |
| 163 | rs78899385 | *PSMA1* | 11 | 14538479 | T | C | C | 0.072 | 0.056 | 0.088 | 2.30E-18 |
| 164 | rs76778262 | *PDE3B* | 11 | 14815707 | C | A | A | 0.072 | 0.052 | 0.093 | 7.63E-12 |
| 165 | rs4752839 | *CELF1* | 11 | 47473650 | A | G | A | 0.025 | 0.016 | 0.034 | 1.30E-08 |
| 166 | rs763648441 | *LTBP3* | 11 | 65546523 | C | A | C |  |  |  |  |
| 167 | rs1938679 |  | 11 | 69457328 | T | C | C | 0.030 | 0.022 | 0.038 | 1.67E-13 |
| 168 | rs645935 | *SERPINH1* | 11 | 75568245 | C | T | T | 0.025 | 0.018 | 0.033 | 2.57E-10 |
| 169 | rs594318 | *FOXRED1* | 11 | 126277818 | C | G | C | -0.010 | -0.018 | -0.002 | 1.79E-02 |
| 170 | rs4763719 | *ETV6* | 12 | 11724486 | G | A | A | 0.025 | 0.017 | 0.033 | 9.26E-10 |
| 171 | rs57454081 |  | 12 | 27950661 | T | C | T | 0.031 | 0.019 | 0.042 | 1.44E-07 |
| 172 | rs76467375 |  | 12 | 46655632 | T | C | T | 0.020 | 0.009 | 0.031 | 4.95E-04 |
| 173 | rs10444558 |  | 12 | 53661701 | T | A | T | 0.020 | 0.011 | 0.029 | 1.14E-05 |
| 174 | rs139121417 | *RAB5B* | 12 | 55986276 | T | C | C | 0.044 | 0.033 | 0.055 | 2.02E-14 |
| 175 | rs59917308 |  | 12 | 56264924 | T | C | T | 0.039 | 0.014 | 0.063 | 1.95E-03 |
| 176 | rs3816804 | *CS* | 12 | 56286961 | T | C | C | 0.085 | 0.075 | 0.096 | 3.58E-56 |
| 177 | rs2277339 | *PRIM1* | 12 | 56752285 | G | T | T | 0.027 | 0.018 | 0.037 | 3.88E-08 |
| 178 | rs10747784 |  | 12 | 57857579 | G | A | G | -0.023 | -0.032 | -0.014 | 2.76E-07 |
| 179 | rs11834895 | *HMGA2, HMGA2-AS1* | 12 | 65853230 | G | C | C | 0.029 | 0.020 | 0.038 | 1.42E-09 |
| 180 | rs151174669 |  | 12 | 65980466 | T | C | C | 0.040 | 0.021 | 0.059 | 3.48E-05 |
| 181 | rs10878984 |  | 12 | 69434754 | C | T | T | -0.026 | -0.035 | -0.018 | 3.12E-10 |
| 182 | rs7971647 | *SOCS2* | 12 | 93590078 | C | T | C | 0.031 | 0.022 | 0.039 | 2.61E-13 |
| 183 | rs3847787 | *CRADD* | 12 | 93813756 | G | A | G | -0.009 | -0.018 | -0.001 | 2.37E-02 |
| 184 | rs80328976 | *WASHC3* | 12 | 102022590 | C | G | G | 0.053 | 0.044 | 0.062 | 4.20E-33 |
| 185 | rs1986854 | *WASHC3* | 12 | 102023697 | C | T | C | 0.036 | 0.024 | 0.048 | 4.39E-09 |
| 186 | rs12424129 | *LINC02456* | 12 | 102281461 | C | T | T | 0.054 | 0.046 | 0.063 | 8.41E-35 |
| 187 | rs12228148 | *LINC02456* | 12 | 102313697 | A | G | A | 0.040 | 0.029 | 0.051 | 2.09E-13 |
| 188 | rs17032833 | *LOC105369944* | 12 | 102561342 | C | T | T | 0.029 | 0.021 | 0.037 | 6.93E-13 |
| 189 | rs117988169 | *HVCN1* | 12 | 110672222 | T | G | G | 0.040 | 0.025 | 0.054 | 5.98E-08 |
| 190 | rs3782886 | *BRAP* | 12 | 111672685 | C | T | T | 0.046 | 0.037 | 0.055 | 4.09E-25 |
| 191 | rs116873087 | *NAA25* | 12 | 112074109 | C | G | G | 0.045 | 0.036 | 0.054 | 7.40E-24 |
| 192 | rs11066359 | *RPH3A* | 12 | 112607850 | T | C | C | 0.033 | 0.025 | 0.041 | 1.55E-15 |
| 193 | rs2072134 | *OAS3* | 12 | 112971371 | A | G | G | 0.036 | 0.025 | 0.046 | 1.64E-11 |
| 194 | rs2093210 | *C14orf39* | 14 | 60490561 | T | C | C | 0.034 | 0.024 | 0.044 | 5.65E-12 |
| 195 | rs910316 | *TMED10* | 14 | 75159339 | A | C | A | -0.010 | -0.020 | 0.001 | 6.31E-02 |
| 196 | rs12590263 | *TC2N* | 14 | 91852154 | A | G | A | 0.023 | 0.015 | 0.031 | 7.06E-09 |
| 197 | rs7143616 | *ATXN3* | 14 | 92065114 | C | A | A | 0.037 | 0.028 | 0.045 | 3.90E-18 |
| 198 | rs7156335 | *ITPK1* | 14 | 92939887 | C | T | C | 0.020 | 0.004 | 0.036 | 1.40E-02 |
| 199 | rs3759556 |  | 14 | 100725962 | G | A | A | 0.047 | 0.029 | 0.065 | 2.33E-07 |
| 200 | rs35443927 |  | 14 | 103383378 | T | C | C | 0.025 | 0.016 | 0.033 | 4.27E-09 |
| 201 | rs12592845 |  | 15 | 48392761 | T | C | C | 0.021 | 0.010 | 0.031 | 9.39E-05 |
| 202 | rs2663534 |  | 15 | 50883261 | C | T | T | 0.024 | 0.016 | 0.032 | 2.29E-09 |
| 203 | rs8041967 | *MIR4713HG, CYP19A1* | 15 | 51252127 | A | G | A | 0.032 | 0.024 | 0.040 | 1.58E-15 |
| 204 | rs28723025 | *CYP19A1* | 15 | 51304114 | A | C | C | 0.028 | 0.019 | 0.037 | 3.27E-10 |
| 205 | rs2162062 | *VPS13C* | 15 | 61987738 | A | G | G | 0.020 | 0.012 | 0.028 | 2.17E-06 |
| 206 | rs975210 | *TLE3* | 15 | 70072013 | A | G | A | 0.034 | 0.022 | 0.045 | 1.26E-08 |
| 207 | rs2415130 | *MYO9A* | 15 | 71950213 | A | G | A | 0.009 | 0.001 | 0.017 | 2.39E-02 |
| 208 | rs55763892 | *PARP6* | 15 | 72247839 | T | C | C | 0.010 | 0.002 | 0.019 | 1.62E-02 |
| 209 | rs750460 | *LOXL1* | 15 | 73949165 | A | G | G | 0.030 | 0.017 | 0.043 | 5.24E-06 |
| 210 | rs8025068 | *ARID3B* | 15 | 74577704 | G | T | G | -0.023 | -0.030 | -0.015 | 1.66E-08 |
| 211 | rs6495171 | *SIN3A* | 15 | 75373282 | A | G | A | -0.030 | -0.039 | -0.021 | 4.45E-11 |
| 212 | rs1526080 | *ADAMTSL3* | 15 | 83921168 | G | A | A | -0.036 | -0.045 | -0.027 | 2.33E-15 |
| 213 | rs938608 | *ACAN* | 15 | 88855374 | T | G | G | 0.035 | 0.025 | 0.045 | 1.51E-12 |
| 214 | rs138351276 | *ACAN* | 15 | 88859943 | G | A | A | 0.110 | 0.083 | 0.138 | 5.03E-15 |
| 215 | rs28456063 |  | 15 | 98637993 | T | C | C | 0.075 | 0.062 | 0.089 | 1.34E-28 |
| 216 | rs897377828 | *IGF1R, IRAIN* | 15 | 98649099 | A | G | G |  |  |  |  |
| 217 | rs2573650 | *ADAMTS17* | 15 | 99973892 | G | A | A | -0.020 | -0.028 | -0.012 | 4.91E-07 |
| 218 | rs4467054 | *ADAMTS17* | 15 | 100255167 | G | T | G | -0.019 | -0.028 | -0.011 | 7.19E-06 |
| 219 | rs4619391 | *WWP2* | 16 | 69780860 | T | A | T | -0.012 | -0.020 | -0.004 | 4.19E-03 |
| 220 | rs114509338 | *SF3B3* | 16 | 70574733 | C | T | T | 0.018 | 0.008 | 0.027 | 2.20E-04 |
| 221 | rs258324 | *CDK10* | 16 | 89687847 | T | G | T | -0.025 | -0.033 | -0.016 | 7.70E-09 |
| 222 | rs116560331 | *POLR2A* | 17 | 7488366 | A | G | G | 0.044 | 0.027 | 0.061 | 2.34E-07 |
| 223 | rs113934718 | *ATAD5* | 17 | 30887862 | A | C | C | 0.033 | 0.021 | 0.045 | 7.34E-08 |
| 224 | rs67474242 | *LRRC37A2, WNT3* | 17 | 46777685 | A | G | G | -0.016 | -0.024 | -0.008 | 7.31E-05 |
| 225 | rs2411374 |  | 17 | 48945636 | C | T | C | -0.022 | -0.030 | -0.013 | 4.54E-07 |
| 226 | rs6504608 | *ZNF652* | 17 | 49347319 | A | C | C | 0.020 | 0.012 | 0.028 | 2.42E-06 |
| 227 | rs9905385 |  | 17 | 61420889 | A | G | A | -0.029 | -0.037 | -0.020 | 1.60E-10 |
| 228 | rs2320125 | *CD79B* | 17 | 63930958 | C | T | C | 0.037 | 0.030 | 0.045 | 1.14E-20 |
| 229 | rs11651289 |  | 17 | 63936114 | T | C | T | 0.055 | 0.036 | 0.074 | 7.12E-09 |
| 230 | rs4239437 | *CABLES1* | 18 | 23152260 | T | C | C | -0.059 | -0.070 | -0.049 | 4.97E-28 |
| 231 | rs9807648 | *TMEM241* | 18 | 23344958 | G | A | G | 0.018 | 0.008 | 0.027 | 1.87E-04 |
| 232 | rs4349223 | *FHOD3* | 18 | 36541412 | A | C | C | 0.011 | 0.004 | 0.019 | 4.20E-03 |
| 233 | rs12606199 | *DYM, LOC100129878* | 18 | 49045546 | A | G | G | 0.024 | 0.015 | 0.033 | 2.76E-07 |
| 234 | rs201707253 | *DYM* | 18 | 49342419 | T | G | G | 0.022 | 0.014 | 0.031 | 4.54E-07 |
| 235 | rs74494415 | *GALR1* | 18 | 77260182 | T | C | C | 0.025 | 0.011 | 0.039 | 4.42E-04 |
| 236 | rs3843750 | *SLC44A2* | 19 | 10637397 | C | G | C | -0.028 | -0.036 | -0.019 | 9.90E-11 |
| 237 | rs1741344 |  | 20 | 4121153 | C | T | C | -0.022 | -0.032 | -0.012 | 8.05E-06 |
| 238 | rs967417 |  | 20 | 6640246 | G | A | G | -0.028 | -0.039 | -0.017 | 2.19E-07 |
| 239 | rs754332 | *KIZ, KIZ-AS1* | 20 | 21197259 | A | G | A | 0.027 | 0.019 | 0.035 | 3.19E-11 |
| 240 | rs3213180 | *E2F1* | 20 | 33675818 | C | G | G | 0.022 | 0.014 | 0.031 | 1.89E-07 |
| 241 | rs143384 | *GDF5* | 20 | 35437976 | G | A | G | 0.062 | 0.053 | 0.071 | 2.65E-44 |
| 242 | rs61016611 |  | 20 | 35624229 | A | G | A | 0.038 | 0.024 | 0.053 | 2.20E-07 |
| 243 | rs3827030 | *PHF20* | 20 | 35887025 | G | A | G | 0.052 | 0.041 | 0.062 | 3.71E-22 |
| 244 | rs8183892 |  | 20 | 36980155 | T | C | T | -0.029 | -0.037 | -0.021 | 3.57E-13 |
| 245 | rs4608 | *RPN2* | 20 | 37236651 | T | C | T | 0.028 | 0.020 | 0.036 | 3.33E-12 |
| 246 | rs2235363 | *ZHX3* | 20 | 41179129 | G | A | G | 0.009 | 0.001 | 0.017 | 2.14E-02 |
| 247 | rs11537645 | *UBE2C* | 20 | 45812764 | G | C | C | 0.020 | 0.006 | 0.034 | 6.01E-03 |
| 248 | rs8121252 | *GNAS* | 20 | 58901754 | T | C | C | -0.029 | -0.038 | -0.021 | 6.73E-11 |
| 249 | rs5754190 | *SYN3, LOC105373002* | 22 | 32654480 | C | T | C | -0.034 | -0.042 | -0.025 | 8.76E-15 |
| 250 | rs4821086 | *SYN3* | 22 | 32687867 | A | C | A |  |  |  |  |
| 251 | rs7290267 | *MIRLET7BHG* | 22 | 46088855 | G | A | G | 0.026 | 0.014 | 0.038 | 3.11E-05 |
| Abbreviations: SNP, single nucleotide polymorphism; No., number; Chr., chromosome; 95% CI, 95% confidence interval; GWAS, genome-wide association study. | | | | | | | | | | | |
| This analysis was under the additive inheritance model and these SNPs were ordered by the chromosome and position. The positions were based on the NCBI GRCh38 version. Gene was identified based on the gene containing the SNP or the nearest gene (within 100 kb up- or downstream) to the SNP. | | | | | | | | | | | |
| The measured height (cm) (phenotype) were stratified by sex, mean-centered, and normalized to one standard deviation (SD) before the height GWAS analysis. | | | | | | | | | | | |
| Beta-value calculation was conducted according to the defined risk alleles. | | | | | | | | | | | |
